# Computational analysis of Ayurvedic metabolites for potential treatment of drug-resistant *Candida auris*

**DOI:** 10.3389/fcimb.2025.1537872

**Published:** 2025-03-13

**Authors:** Mohibullah Shah, Mahnoor Zia, Iqra Ahmad, Muhammad Umer Khan, Hasan Ejaz, Maqsood Alam, Shahid Aziz, Umar Nishan, Hanna Dib, Riaz Ullah, Suvash Chandra Ojha

**Affiliations:** ^1^ Department of Biochemistry, Bahauddin Zakariya University, Multan, Pakistan; ^2^ Institute of Molecular Biology and Biotechnology, The University of Lahore, Lahore, Pakistan; ^3^ Department of Clinical Laboratory Sciences, College of Applied Medical Sciences, Jouf University, Sakaka, Saudi Arabia; ^4^ Department of Biochemistry and Molecular Biology, Federal University of Ceara, Fortaleza, Brazil; ^5^ Department of Chemistry, Kohat University of Science and Technology, Kohat, Pakistan; ^6^ College of Engineering and Technology, American University of the Middle East, Egaila 54200, Kuwait; ^7^ Department of Pharmacognosy, College of Pharmacy, King Saud University, Riyadh, Saudi Arabia; ^8^ Department of Infectious Diseases, The Affiliated Hospital of Southwest Medical University, Luzhou, China

**Keywords:** fungal infections, *Candida auris*, computational chemistry, plants, molecular docking

## Abstract

This study explored the effectiveness of secondary metabolites of referred traditional Ayurvedic plants in treating fungal infections, particularly targeting *Candida auris*. Recognized as a global health threat, this fungus is notorious for its resistance to several antifungal treatments. The inhibition of lanosterol 14α-demethylase causes the depletion of ergosterol, ultimately resulting in the inhibition of fungal cell growth. A total of 469 metabolites, including alkaloids, flavonoids, and tannins from Ayurvedic plants, were screened against CYP51 (PDB ID: 4UYL) using molecular docking. Key active site residues, namely HIS461, CYS463, and TYR122, were targeted to inhibit the ergosterol synthesis, with VNI employed to benchmark the findings. Shortlisted metabolites underwent physicochemical analysis, ADMET analyses, and the principles of medicinal chemistry, which were confirmed through pharmacokinetic simulations. Further, this study investigated the molecular dynamics (MD) of co-crystalized VNI, trans-p-coumaric acid, and MCPHB [(r)-n-(1’-methoxycarbonyl-2’-phenylethyl)-4-hydroxybenzamide] to evaluate RMSD, RMSF, Rg, SASA, cross-correlation of residue motions, PCA, and free energy decomposition. The top compounds demonstrated favorable drug-like criteria. They exhibited good absorption potential with high gastrointestinal uptake. Distribution and metabolism were manageable with low risks of drug-drug interactions. Excretion profiles indicated proper clearance, and toxicity assessments showed low potential for cardiovascular issues. The results showed stable interactions for trans-p-coumaric acid and MCPHB, suggesting that all the ligands maintain stable binding interactions with the protein, which preserves structural integrity across all systems. This comprehensive approach suggests that these natural metabolites from Ayurvedic medicine could potentially serve as primary agents against fungal diseases, pending further validation through controlled *in vitro* and *in vivo* clinical trials.

## Introduction

Fungal infections are emerging as a serious healthcare issue in humans and animals. Such infections in humans have been historically neglected, despite the death of about 1.5 million people annually from fungal diseases. In the last ten years, understanding of the underlying mechanisms of these infections has been significantly improved. This progress is achieved by gaining an important understanding of the elements related to both the host and the pathogen, which have a role in determining the characteristics and seriousness of these diseases (Brown et al., 2024). Epidemiological studies have suggested that fungus can cause various superficial and systematic infections, and patients with weakened immune systems are more susceptible to infection (De Pauw et al., 2008; Sagatova et al., 2018). Most of the infections, including fungal asthma, chronic pulmonary aspergillosis, fungal keratitis, invasive candidiasis, fungal pneumonia, invasive aspergillosis, cryptococcal meningitis in HIV/AIDS patients, and disseminated histoplasmosis, are linked with fungal diseases (Denning, 2015; Bongomin et al., 2017; Rajasingham et al., 2017).

A novel Candida species, isolated from a patient’s external auditory canal, is a deadly, multiple-drug-resistant fungal, pathogenic yeast first reported in Japan in 2009 (Satoh et al., 2009). The azoles, i.e., fluconazole, itraconazole, voriconazole, and posaconazole, as well as polyenes and echinocandins classes, are used as the first-line treatment and prevention of fungal infections, including fungemia caused by *C. auris* (Perfect, 2017; Shaban et al., 2020). However, the extensive and prolonged use of these antifungal drugs has caused drug resistance in these pathogens (Wickes, 2020). Due to the resistance of antifungal drugs, there is a need to develop new therapeutic approaches against pathogenic fungi in treating various diseases.

Lanosterol 14α-demethylase (CYP51) enzyme is a potential target for antifungal drug discovery. It belongs to the superfamily of cytochrome P450 (Aoyama et al., 1996). Ergosterol is the fungal-specific sterol involved in maintaining the integrity, fluidity, and permeability of the cell membrane structure (Monk and Keniya, 2021). Targeting the CYP51 enzyme causes the depletion of ergosterol and the accumulation of lanosterol with other methyl sterols, ultimately resulting in the inhibition of fungal cell growth (Aoyama et al., 1996). Moreover, the lack of ergosterol by CYP51 inhibition results in the weakening of biofilm in *C. auris* (Harikrishnan et al., 2022). Biofilm is the most significant pathogenicity feature of *C. auris*, which makes it a “superbug”, as biofilm formation causes increased resistance to antifungal agents. Hence, inhibiting CYP51 in *C. auris* is a promising strategy for reducing biofilm formation and combating this multidrug-resistant pathogen.

Natural bioactive metabolites from medicinal plants have gained importance in the recent era of antifungal drug discovery (Qadri et al., 2013). In the Ayurvedic medicinal system, about 7500 plants are used for their antipyretic, antifungal, antibacterial, and anti-diabetic properties (Joshi and Dhawan, 2005; Saini et al., 2015; Nahar and Sarker, 2019). Currently, computer-aided drug discovery (CADD) is being popularly used for the discovery of new and potent drugs by screening a large number of plant-derived molecules and evaluating their toxicity. Its major benefit is the reduced cost and time (Macalino et al., 2015). Various CADD studies have been done to explore the new bioactive metabolites from natural plants that contain antifungal activity. The metabolites of Caesalpinia bonduc seeds and *Piper crocatum* (red betel leaf) inhibit ergosterol synthesis by targeting the CYP51 enzyme (Sasidharan et al., 2023; Siswina et al., 2023). Other studies by Shalini A. Shinde et al. and Sakyiamah et al (Sakyiamah et al., 2022; Shinde et al., 2024) have also reported promising antifungal potential of plant-derived metabolites by inhibiting the CYP51 enzyme.

The increasing prevalence of antifungal resistance, particularly for *C. auris*, poses a significant challenge to public health, creating an urgent need for novel inhibitors by targeting the CYP51 enzyme. Targeting this enzyme causes the depletion of ergosterol, contributing a potential pathway for novel anti-fungal therapeutics. This study particularly aims to identify potent bioactive metabolites from Ayurvedic medicinal plants, namely *Swertia chirayita* (Roxb*.)* H.Karst. [Gentianaceae] (Jauhari et al., 2017), *Abutilon indicum* (L.) Sweet [Malvaceae] (Kuo et al., 2008; Gomaa et al., 2018), *Phyllanthus emblica* L. [Phyllanthaceae] (Gaire and Subedi, 2014), *Nelumbo nucifera Gaertn*. [Nelumbonaceae] (Chen et al., 2007; Rho and Yoon, 2017), *Alangium salviifolium* (L.f.) Wangerin [Cornaceae] (Venkateshwarlu et al., 2011; Meenakshi and Rajesh, 2015), *Rauvolfia serpentina* (L.) Benth. exKurz [Apocynaceae] (Kumari et al., 2013), *Asparagus racemosus Willd*. [Asparagaceae] (Singaravadivel, 2014), and *Abroma augustum* (L.) L.f. [Malvaceae] (Rakesh et al., 2023). They have been reported for their medicinal activity, including antiviral, antibacterial, anti-inflammatory, and particularly antifungal activity. Our research hypothesizes that phytochemicals from these plants can effectively inhibit CYP51, thereby combating antifungal resistance. We employed molecular docking, drug-likeness, and ADME analyses, molecular dynamics simulations, and pharmacophore modeling. The significance of this study lies in its potential to discover new therapeutic agents that could be developed into drugs to address antifungal drug resistance and notable benefits over current treatments.

## Materials and methods

### Ligand selection

A total of 469 metabolites from Ayurvedic medicinal plants were retrieved in the meticulous literature study. These include 118 metabolites from *S. chirayita* (Roxb.) H.Karst. [Gentianaceae], 50 metabolites from *A. indicum* (L.) Sweet [Malvaceae], 73 metabolites from *P. emblica* L. [Phyllanthaceae], 122 metabolites from *N. nucifera Gaertn*. [Nelumbonaceae], 37 metabolites from *A. salviifolium* (L.f.) Wangerin [Cornaceae], 14 metabolites from *R. serpentina* (L.) Benth. ex Kurz [Apocynaceae], 47 metabolites from *A. racemosus Willd*. [Asparagaceae], and 8 metabolites from *A. augustum* (L.) L.f. [Malvaceae]. The structures of these metabolites were obtained from the PubChem database in mol format. For those structures that were unavailable in PubChem, we employed the ChemDraw 12.0 program and Canonical SMILES for drawing and then saving them in mol files.

VNI [(R)-N-(1-(2,4-dichlorophenyl)-2-(1H-imidazol-1-yl)ethyl)-4-(5-phenyl-1,3,4-oxadiazol-2-yl)benzamide)], the native ligand of 4UYL, was selected as a reference in our study (Shamsuddin et al., 2021). VNI was chosen as the standard compound for our study due to its proven efficacy against pathogens, favorable pharmacokinetics, and low toxicity. Its superior effectiveness against A. fumigatus makes it a suitable model for evaluating plant metabolites that target the CYP51 enzyme in treating *C. auris* infections (Friggeri et al., 2018).

### Ligand preparation

A database was prepared in which all the ligands were converted into their 3D structures with proper tautomers, ionization states, chiralities, and bond orders and used as input for molecular docking and saved in MDL Molfile (mol). All the ligands were stabilized by energy minimization and further processed by using the default parameters in the molecular operating environment software (Molecular Operating Environment (MOE), 2022.02 Chemical Computing Group ULC, 1010 Sherbooke St. West, Suite #910, Montreal, QC, Canada, H3A 2R7, MOE2022.v11.18.1). This precision ensures accurate modeling of ligand behavior in biological systems, improves prediction of ligand-protein interactions, and enhances the reliability of computational methods.

### Target protein selection

The three-dimensional crystal structure of cytochrome P450 sterol 14α-demethylase was obtained from the RCSB protein data bank (PDB ID: 4UYL). The protein PDB ID was selected based on the presence of a co-crystallized ligand (VNI) in the active site, a resolution of 2.81 Å, and its crystallization through the X-ray crystallography technique.

### Target protein preparation

Protein preparation is an important step for molecular docking analysis (Shah et al., 2021). The protein structure was prepared through MOE software. All the unwanted chains were deleted; only unique chain A was retained for further analysis. All the attached water molecules and ligands with the protein structure were removed in the refinement process to avoid interaction during the optimization and minimization processes. Then the protonation was done to ensure that all the hydrogen atoms were properly assigned for the proper molecular interactions. Afterwards, the energy minimization was performed to ensure that the protein structure is stable and there are no hindrances or strains during interactions (Yamin et al., 2024).

### Active site determination

The active site of the target protein was determined through MOE software by using the SiteFinder tool in MOE. This tool predicted various active sites of different sizes. Among them, the active site was chosen that aligned with the location of the co-crystallized ligand (VNI), which also served as the standard in our study. This active site contained specific residues that have been previously highlighted in literature for their essential role in protein activity (Shamsuddin et al., 2021).

### Docking validation

For the validation of docking within the active site of the target protein, the retrieved PDB complex (PDB 4UYL) and the docked complex of VNI (prepared through MOE) were superimposed through PyMOL software (Molecular Graphics System, Version 3.0 Schrödinger, LLC). The root mean square deviation (RMSD) was calculated. The RMSD value less than 2 Å was considered optimal as it justified the accuracy of docking parameters (Khalid et al., 2024).

### Molecular docking

Molecular docking is an important computational technique, and its key aim is to determine the potential binding geometries of a ligand of a known three-dimensional structure with a target protein (Muhammad et al., 2020; Kumari et al., 2023). The selected 469 metabolites, alongside the standard VNI, were docked with the CYP51 target protein to identify the best ergosterol synthesis inhibitor. The docking was achieved through an induced fit model of MOE 2022.02 software.

The docking process was conducted in multiple phases to ensure the most favorable interactions between the ligands and the target proteins. The Triangle Matcher Algorithm was initially employed to establish ligand positioning. In the initial rescoring step, the London dG correction was implemented with a retention value of 10. After the initial scoring, a refinement step was carried out using the forcefield approach to adjust the placement of the ligands in the active structures. For the second rescoring phase, the GBVI/WSA dG method was used, with a retention value of 5. For the final analysis, our method guaranteed that only the most energetically favorable ligand positions were considered. Duplicates were eliminated at every stage of the procedure.

The highest-scored pose of each ligand based on the “S” score and binding energy was considered. The scores of ligands were compared with those of VNI as the reference inhibitor. Ligands with a higher docking score than the reference metabolite were selected for further investigations (Kumari et al., 2023).

### Drug likeness and physicochemical analysis

The drug-likeness and physicochemical analysis of selected metabolites were analyzed using the SwissADME online tool (http://www.swissadme.ch/index.php). This analysis provides guidelines that increase the probability of the chemical passing clinical trials (Ursu et al., 2011). SwissADME software comprises five rules of drug likeness, namely, Lipinski (Lipinski et al., 1997), Ghose (Ghose et al., 1999), Veber (Veber et al., 2002), Egan (Egan et al., 2000a), and Muegge (Muegge et al., 2001). If the selected metabolites are unable to follow the maximum rules of drug-likeness, they may not be considered orally active.

### Protein-ligand interaction

Protein-ligand interaction was evaluated to assess how the target protein interacts with the ligand using BIOVIA Discovery Studio software. This tool is used to simulate, analyze, and visualize chemical and biological systems, including drawing 2D and 3D structures of target protein complexes.

### Molecular dynamics simulation

MD simulations were conducted to explore the dynamic interactions of the top 2 metabolites, namely trans-p-coumaric acid and MCPHB, with the CYP51 protein, alongside the co-crystallized ligand VNI. The selected top-hit complexes with CYP51 receptors were obtained from docked models. These selected complexes underwent 100 ns MD simulations for re-refining as well as stabilization. Following prior practice (Khalid et al., 2024), partial atomic charges for the ligands were assigned using the Antechamber module of the AMBER20 package (Arantes et al., 2021). The ligands and receptors were subjected to the leap module to fill in the missing hydrogens, neutralize the system, solvate the complexes, and finally generate the parameter files and coordinates for the simulation system. The dynamic parameters of the ligands and receptors were characterized under the generalized Amber force field (GAFF) and ff14SB, respectively. The counter ions such as Cl or Naþ were used to neutralize the proton-containing protein, followed by the solvation of the complex using an octahedral TIP3P box with dimensions of 10.0 x 10.0 x 10.0 Å. The resultant solvated complexes were saved in PDB format; subsequently, the parameter and coordinate files were created at the final step of the Leap module. Afterward, the steric clashes were removed by minimizing the complexes in three sequential steps. Initially, both receptors and ligands were optimized using ions and a solvated water system. In the next step, the optimization of residues of the pocket with backbone amino acids and proteins was optimized. Finally, the optimization of the whole system was performed to relax the complexes with the proteins. Notably, all steps of minimization were carried out with 2500 steps of steepest descent and 5000 steps of conjugate gradient. At the end of minimization, the whole system was heated by progressively raising the temperature from 0 to 300 K. An additional equilibration step was executed at 300 K to further stabilize the system. The Langevin dynamics was used to execute the equilibration process by keeping the collision frequency at 1 ps^-1^ and maintaining the force constant at 10 kcal (mol Å^2^)^-1^ (Raza et al., 2023).

After stabilizing the system, MD simulations were conducted for 100 ns by applying 300 K of NPT ensemble and 1 atm of atmospheric pressure. Finally, at the end of simulations for all selected complexes, the CPPTRAJ module of AMBER20 was used to analyze the RMSD, RMSF, and SASA. Moreover, the 2D-RMSD, radius of gyration (Rg), principal component analysis (PCA), and cross-correlation (DCCM) for the trajectories from the last 50 ns of MD simulations were performed by following the methodology developed by Arantes et al (Arantes et al., 2021). Additionally, to gain knowledge about the dynamic stability and sampling patterns, the RMSD and 2D-RMSD were computed for the selected ligands as well as for the apoprotein complex.

### Binding free energy calculations by MMGBSA/MMPBSA and Per-residue-free energy decomposition analysis

The binding free energy (BFE) was calculated using molecular mechanics-based analysis consisting of molecular mechanics (MMPBSA/MMGBSA) modules integrated into the AMBER17 software to examine the structural and energetic properties of the selected complexes. MM/GBSA computations were done for each complex system using 1000 snapshots from the last 2 ns MD trajectories. The binding free energy was calculated as the difference between the total free energy of the ligand-protein complex (Gcom) and the sum of the free energies of individual proteins (Gpro) and ligands (Glig) (Equation 1) (Chohan et al., 2020):


(1) $$\text{Δ}{\text{G}}_{\text{bind}}=\text{ΔH}–\text{TΔS }=\text{Δ}{\text{G}}_{\text{com}}–\;\left(\text{Δ}{\text{G}}_{\text{CYP}51}+\text{Δ}{\text{G}}_{\text{Iig}}\right)$$

Subsequently, the free energy of the ligand-protein complex, protein, and ligand was also calculated (Equation 2):


(2) $$\text{ΔG }=\text{Δ}{\text{E}}_{\text{MM}}–\text{TΔS }+\text{Δ}{\text{G}}_{\text{sol}}$$

The molecular mechanics were further investigated by fragmenting it into Van der Waals energies (EvdW), non-bonded electrostatic energies (Eele), and solvation-free energy (G_sol_), which was further subdivided into polar and non-polar solvation energies.

For more insight into the BFE, per-residue energy decomposition was estimated through vdW (G_vdW_), electrostatic (G_ele_), polar (G_ele, sol_), and nonpolar (G_nonpol, sol_). The decomposition parameters were evaluated based on the binding free energy snapshots. The analysis of per-residue free energy decomposition offers an advantage over binding free energy by allowing a more comprehensive analysis of the binding affinity as well as the selectivity of each inhibitor. Thereby, helping the researchers to design more effective inhibitors targeting the specific interactions or regions (Asif et al., 2022; Khalid et al., 2022).

### Generation of essential pharmacophores

A high-quality pharmacophore model was developed with the Pharmacophore Query Editor in MOE software as reported earlier (Ahmad et al., 2024; Khalid et al., 2024). This tool can generate a variety of predefined pharmacophore features, such as hydrogen bond donor (Don), hydrogen bond acceptor (Acc), aromatic center (Aro), Pi ring center (PiR), aromatic ring or Pi ring normal (PiN), hydrophobic (Hyd), anionic atom (Ani), and cationic atom (Cat). After a comprehensive analysis of protein-ligand interactions among the predominant metabolites, unique interactions were identified and labeled using a single structure framework, resulting in the development of a comprehensive pharmacophore. The radius of the detected pharmacophore features was standardized to 1.0 Å, and the distances between the features were measured (Ahmad et al., 2024).

### Bioavailability score analysis

The bioavailability score analysis of metabolites was evaluated through the SwissADME online tool. It predicts the bioavailability of metabolites through different physicochemical properties, including lipophilicity (XLOGP3), molecular size (MW), polarity (TPSA), solubility (log S), unsaturation, and flexibility. The optimal range for each physicochemical property includes lipophilicity: XLOGP3 between −0.7 and +5.0, size: MW between 150 and 500 g/mol, polarity: TPSA between 20 and 130 Å^2^, solubility: log S not higher than 6, saturation: fraction of carbons in the sp^3^ hybridization not less than 0.25, and flexibility: no more than 9 rotatable bonds (Daina et al., 2017).

### Medicinal chemistry

The medicinal chemistry of the metabolites was evaluated through the SwissADME tool. The important parameters of medicinal chemistry include PAINS (pan-assay interference compounds), Brenk (structure alerts), lead likeness, and synthetic accessibility. The PAINS alert identifies the metabolites by finding false positive results. PAINS predicts the substructure of the metabolite that exhibits biological activity. The lead likeness of metabolites can also be predicted to have a probability of being a lead metabolite in drug discovery, and it increases the chances of chemicals in clinical trials. Synthetic accessibility describes the synthetic process, and its scoring ranges from 1 to 10. The lower value predicts the simple synthetic process, and higher values predict the complex synthetic process (Bakchi et al., 2022).

### Pharmacokinetics analysis

ADMET (Absorption, Distribution, Metabolism, Excretion, and Toxicity) analysis of drugs plays a significant role in drug development (Jia et al., 2020). The ADMET properties of selected metabolites were explained through ADMETlab 2.0 software (https://admetmesh.scbdd.com/). For a drug to have appropriate and safe therapeutic effects, it must be easily absorbed, distributed, and metabolized within the body. That drug should pass out from the body within the expected duration (Uzzaman et al., 2023). There are several parameters involved in ADMET analysis. The factors include human intestinal absorption, permeability of the blood-brain barrier, inhibition of the cytochrome P450 enzyme, cardiotoxicity, and cytotoxicity.

### Pharmacokinetic simulation

The pharmacokinetic simulation was aimed at predicting the human plasma concentration-time profile of the hit metabolites by using their ADMET profile as input. Physiologically-based pharmacokinetic (PBPK) modeling was performed using PK-Sim software to predict the plasma concentration-time profiles of the metabolites (Willmann et al., 2012; Frechen et al., 2021).

The plasma was selected as the target compartment with a focus on achieving adequate drug concentrations for the effective treatment of *C. auris* infections, including bloodstream infections (candidemia) and deep-seated infections. A virtual population of older adults (>65 years) was simulated, representing the target demographic for *C. auris* treatment, as this age group is more susceptible to such infections due to underlying medical conditions, immunocompromised states, and frequent hospitalizations.

The administration protocol was based on the typical administration of echinocandins, which are often the first-line treatment for invasive *C. auris* infections. Intravenous (IV) infusion was selected as the route of administration with an infusion time set to 60 minutes.

The simulations were performed using a standard model for small molecules in plasma (unbound). The parameters of the simulation include the physicochemical properties of metabolites, including MW, lipophilicity, plasma protein binding, and solubility, which were taken as input into the model. The unbound fraction of the drug in the plasma compartment was selected for analysis.

### Target prediction analysis

An important analysis of the top metabolites was performed through the Swiss Target Prediction Server (http://www.swisstargetprediction.ch) to evaluate their interaction with human targets. For this purpose, SMILES of top metabolites were generated and used as input files. The results were generated for the most likely target of the query molecule.

## Results and discussion

### Potential active site

The selected active site contained residues, i.e., LEU109, TYR122, THR126, PHE130, VAL135, TYR136, LEU143, GLN146, LYS147, VAL150, LEU154, LEU205, PHE229, ASP276, MET300, ALA303, LEU304, MET306, ALA307, GLY308, HIS310, SER311, SER312, ILE373, HIS374, SER375, ILE376, ILE377, ARG378, ARG460, HIS461, ARG462, CYS463, ILE464, GLY465, PHE468, ALA469, GLN472, LEU503, and PHE504. This active site contains specific residues that have been previously highlighted in literature for their essential role in protein activity (Hargrove et al., 2015; Tiwari et al., 2018).

**Figure 1:**
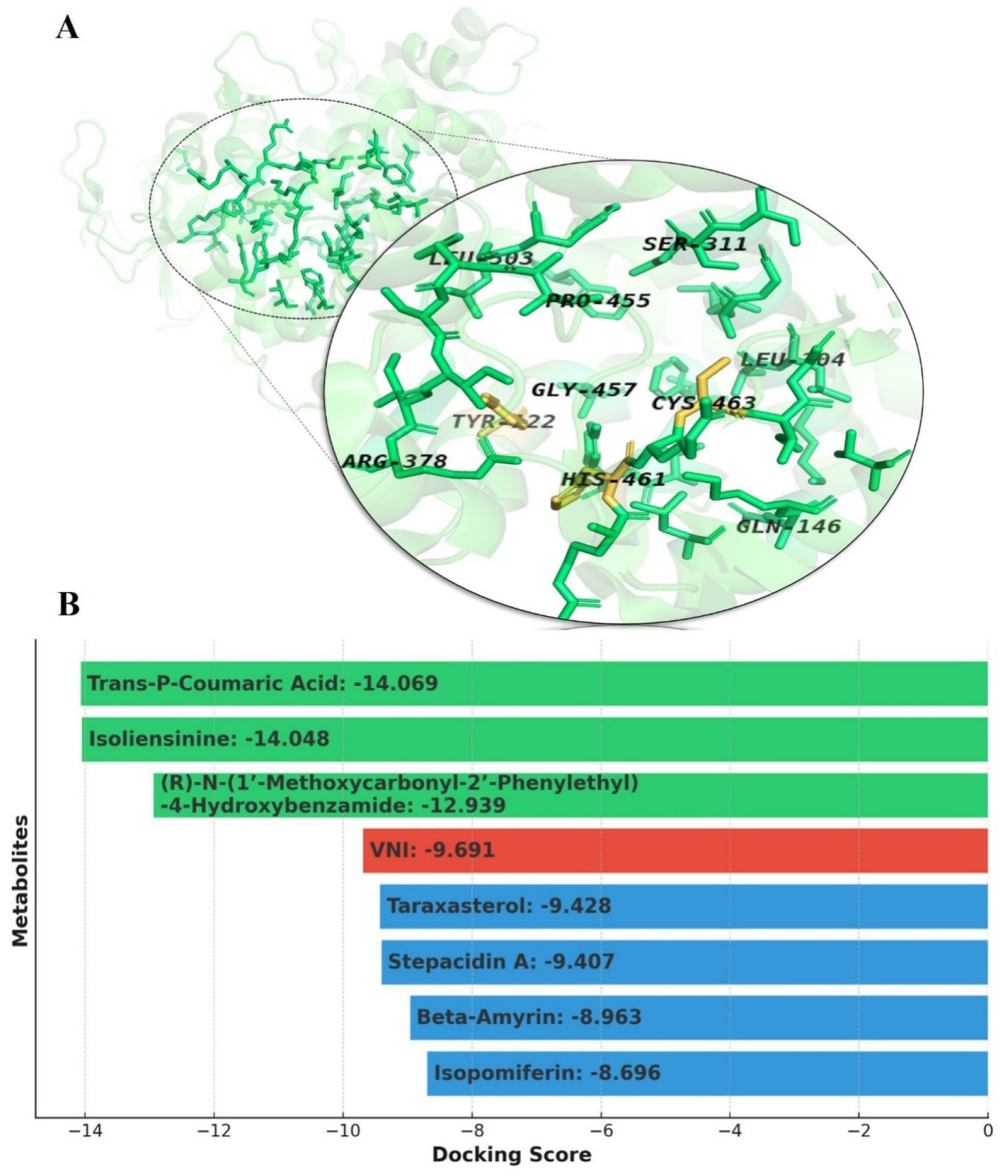
Panel **(A)** represents active site residues (green) of the target protein Lanosterol 14α demethylase (CYP51). Orange-highlighted residues, namely HIS461, CYS463, and TRY122, are essential for the catalytic role of CYP51 in ergosterol production. HIS461 facilitates proton transport and acts as an acidic/basic catalyst, while CYS463 serves as the heme iron ligand essential for catalytic activity. Tyr122 stabilizes the protein structure by forming a hydrogen bond with the heme ring. Panel **(B)** represents the comparison of docking scores of our top 3 metabolites (green) against the standard inhibitor VNI (red) and other known inhibitors (blue), demonstrating the superior binding affinity of our top metabolites to the target protein.

The enzyme CYP51 has conserved residues essential for its catalytic role in ergosterol production (**Figure 1A**). HIS461 facilitates catalytic function and depends on proton transport to the active site (Hargrove et al., 2015). Therefore, it displays a significant role as an acidic and basic catalyst (Tiwari et al., 2018). CYS463 acts as the heme iron proximal ligand, which is essential to the enzyme’s catalytic activity. Tyr122 is invariant across the whole CYP51 family of target proteins; it forms the hydrogen bond with the heme ring. A propionate is important for the stability of protein structure, but the binding of strong inhibitors often disrupts this hydrogen bond. TYR122, ARG378, HIS461, and CYS463 residues are conserved with target CYP51 family members, which underscore their vital involvement in catalytic enzymatic function (Hargrove et al., 2015). Furthermore, it has been observed that HIS461 and LEU503 are involved in bond formation in standard (VNI) interaction analysis (Shamsuddin et al., 2021). Understanding the particular roles of these residues helps us understand CYP51’s mechanistic features and provides a deep understanding of antifungal drug development targeting this vital enzyme.

### Molecular docking

In this docking analysis, binding energies of all metabolites were calculated, which shows sixteen metabolites, namely, Gammacer-16-en-3-β-ol, Swerta-7,9(11)-dien-3-β-ol, Pichierenol, Methyl (3β,13α,14β,20α)-3-hydroxy-13-methyl-26-norolean-8-en-29-oate, Olean-12-ene-18αH-3-one-9α-ol from *S. chirayita* (Roxb.) H.Karst. [Gentianaceae], mallonin, putranjivain A, camphor, borneol, methyl-4-hydroxybenzoate from *P. emblica* L. [Phyllanthaceae], 3S,5R-Dihydroxy-6S,7-megastigmadien-9-one, (6R,6aR) roemerine-N(β)-oxide, Dihydrophaseic acid 3’-O-β-D-glucopyranoside from *N. nucifera Gaertn.* [Nelumbonaceae], Rauwolfine, Ajmaline from *R. serpentina* (L.) *Benth.* ex Kurz [Apocynaceae], Shatavarin-V from *A. racemosus Willd*. [Asparagaceae], and Abromasterol from *A. augustum* (L.) L.f. [Malvaceae] did not show any interaction with the CYP51 target protein (**Supplementary Table S1**). This could be due to the structural complexity and diversity of these metabolites, which can cause hindrance in the generation of a suitable conformation for docking. Hence, it is clear that these metabolites are not effective as potential inhibitors for the CYP51 target protein.

The docking score of the reference ligand was observed as 9.69 kcal/mol (**Supplementary Table S1**). It was found that 169 metabolites exhibited a higher docking score than the reference metabolite. Among them, the top 30 metabolites whose docking scores ranged from -18.3 kcal/mol to -12.8 kcal/mol (**Supplementary Table S1**). These top 30 metabolites were studied for their drug-likeness and physicochemical properties before proceeding with the interaction analysis. This approach ensured the selection of not only strong inhibitors but also potentially safe compounds.

The docking analysis indicates that the three most prominent metabolites identified in our study, namely trans-p-coumaric acid (-14.069), isoliensinine (-14.048), and MCPHB [(r)-n-(1’-methoxycarbonyl-2’-phenylethyl)-4-hydroxybenzamide](-12.939), exhibit considerably higher binding affinities to the target protein in comparison to the standard, VNI (-9.691 kcal/mol) (**Figure 1B**). Significantly, these metabolites exhibit greater performance compared to other broadly investigated inhibitors of CYP51, such as taraxasterol (-9.428), stepacidin A (-9.407), beta-amyrin (-8.963), and isopomiferin (-8.696), suggesting a stronger interaction and potential effectiveness. Therefore, the results emphasize the potential of our leading compounds in producing superior inhibitors, strengthening their ability to generate more favorable therapeutic results compared to other identified inhibitors of CYP51.

### Docking protocol validation

For the docking validation process, the complexes of docked and PDB reference compounds were prepared through MOE software. The docked complex was superimposed on the reference co-crystallized complex using PyMOL, and the root mean square deviation (RMSD) was calculated. During the alignment, initially, 470 residues participated from both complexes with a MatchAlign score of 2491.000. Afterwards, 7 residues were rejected during cycle 1 (RMSD=1.04) and 3 during cycle 2 (RMSD=0.99). Finally, the executive RMSD of 0.980 (460 to 460 atoms) was obtained. The observed RMSD for our docked complex is 0.980 A° within the optimal range, which signifies a high degree of alignment that represents the accuracy and reliability of our docking protocol with the reference PDB complex. The consistency in docking results of our metabolite database using the same active site reinforces the validity of our computational approach in ligand docking studies (**Supplementary Figure S1**).

### Drug likeness and physicochemical analysis

Based on their binding energy, the top 30 metabolites were subjected to drug-likeness analysis through Swiss ADME (**Table 1**). Among them, the top ten metabolites were selected based on their adherence to the maximum drug-likeness rules, which can be explained by their optimal physicochemical profiles (**Supplementary Table S2**). These selected metabolites: 4-hydroxybenzoate follows (3 rules, namely Lipinski’s rule, Egan’s rule, and Veber’s rule); methylcoumarate, 2,6-dihydroxy-4-methoxyacetophenone (4 rules, Lipinski’s rule, Ghose’s rule, Veber’s rule, and Egan’s rule); trans-p-coumaric acid (4 rules, namely Lipinski’s rule, Ghose’s rule, Veber’s rule, and Egan’s rule); these three metabolites, isoliensinine, neferine, and liensinine, follow (3 rules, namely Lipinski’s rule, Veber’s rule, and Egan’s rule). Another three metabolites, eudesmic acid, scoparone, and MCPHB, followed all rules of drug-likeness (5 rules: Lipinski’s rule, Veber’s rule, Egan’s rule, Ghose’s rule, and Muegge’s rule). These metabolites followed all requirements of drug-likeness analysis, which plays a significant role in drug development. These metabolites are probable lead metabolites in drug discovery, and they increase the chances of chemicals in clinical trials.

**Table 1:** Drug likeness analysis of top metabolites by SwissADME webserver.

Sr. No.	Metabolites			DRUG LIKENESS				
		Lipinski	Ghose	Veber	Egan	Muegge	Comments	Bioavailability Score
**1. **	**Aristophyll-C**	–	–	–	–	–	Not accepted in SwissADME due to character length exceeding 200	–
**2. **	**Pheophytin-a**	–	–	–	–	–	Not accepted in SwissADME due to character length exceeding 200	–
**3. **	**Alamandine**	No; MW>500, NorO>10, NHorOH>5	No; MW>480, WLOGP<-0.4, MR>130, #atoms>70	No; Rotors>10, TPSA>140	No; TPSA>131.6	No; MW>600, XLOGP3<-2, TPSA>150, Rotors>15, H acc>10, H-don>5	Followed zero rules	0.17
**4. **	**1-Hentetracontanol**	No; MW>500, MLOGP>4.15	No; MW>480, WLOGP>5.6, MR>130, #atoms>70	No; Rotors>10	No; WLOGP>5.88	No; XLOGP3>5, Heteroatoms<2Rotors>15	Followed zero rules	0.17
**5. **	**Racemoside B**	No; MW>500, NorO>10, NHorOH>5	No; MW>480, MR>130, #atoms>70	No; TPSA>140	No; TPSA>131.6	No; MW>600, TPSA>150, #rings>7, H-acc>10, H-don>5	Followed zero rules	0.17
**6. **	**Nelumboroside B**	No; MW>500, NorO>10, NHorOH>5	No; MW>480, WLOGP<-0.4, MR>130, #atoms>70	No; TPSA>140	No; TPSA>131.6	No; MW>600, XLOGP3<-2, TPSA>150, H-acc>10, H-don>5	Followed zero rules	0.17
**7. **	**4-Hydroxybenzoate**	Yes	No MW<160, MR<40, #atoms<20	Yes	Yes	No; MW<200	Followed three rules	0.85
**8. **	**Methylcoumarate**	Yes	Yes	Yes	Yes	No; MW<200	Followed four rules	0.55
**9. **	**2,6-Dihydroxy-4-methoxyacetophenone**	Yes	Yes	Yes	Yes	No; MW<200	Followed four rules	0.55
**10. **	**trans-p-coumaric acid**	Yes	Yes	Yes	Yes	No; MW<200	Followed four rules	0.85
**11. **	**Isoliensinine**	Yes; MW>500	No; MW>480, MR>130, #atoms>70	Yes	Yes	No; MW>600, XLOGP3>5	Followed three rules	0.55
**12. **	**Tritriacontane-9,10-diol**	Yes; MLOGP>4.15	No; MW>480, WLOGP>5.6, MR>130, #atoms>70	No; Rotors>10	No; WLOGP>5.88	No; XLOGP3>5, Rotors>15	Followed one rules	0.55
**13. **	**Shatavarin-VII**	No; MW>500, NorO>10, NHorOH>5	No; MW>480, MR>130, #atoms>70	No; TPSA>140	No; TPSA>131.6	No; MW>600, TPSA>150, #rings>7, H-acc>10, H-don>5	Followed zero rules	0.17
**14. **	**Rescinnamidine**	No; MW>500, NorO>10	No; MW>480, MR>130, #atoms>70	No; Rotors>10	Yes	No; MW>600	Followed one rules	0.17
**15. **	**Hentriacontane-12,15-diol**	Yes; MLOGP>4.15	No; WLOGP>5.6, MR>130, #atoms>70	No; Rotors>10	No; WLOGP>5.88	No; XLOGP3>5, Rotors>15	Followed one rules	0.55
**16. **	**Neferine**	Yes; MW>500	No; MW>480, MR>130, #atoms>70	Yes	Yes	No; MW>600, XLOGP3>5	Followed three rules	0.55
**17. **	**Nonacosane-10,13-diol**	Yes; MLOGP>4.15	No; WLOGP>5.6, MR>130, #atoms>70	No; Rotors>10	No; WLOGP>5.88	No; XLOGP3>5, Rotors>15	Followed one rules	0.55
**18. **	**Rescinnamine**	No; MW>500, NorO>10	No; MW>480, MR>130, #atoms>70	No; Rotors>10	Yes	No; MW>600	Followed one rules	0.17
**19. **	**Shatavarin VI**	No; MW>500, NorO>10, NHorOH>5	No; MW>480, MR>130, #atoms>70	No; TPSA>140	No; TPSA>131.6	No; MW>600, TPSA>150, #rings>7, H-acc>10, H-don>5	Followed zero rules	0.17
**20. **	**Eudesmic acid**	Yes	Yes	Yes	Yes	Yes	Followed all five rules	0.85
**21. **	**Liensinine**	Yes; MW>500	No; MW>480, MR>130, #atoms>70	Yes	Yes	No; MW>600, XLOGP3>5	Followed three rules	0.55
**22. **	**Scoparone**	Yes	Yes	Yes	Yes	Yes	Followed all five rules	0.55
**23. **	**Racemoside c**	No; MW>500, NorO>10, NHorOH>5	No; MW>480, MR>130, #atoms>70	No; TPSA>140	No; TPSA>131.6	No; MW>600, TPSA>150, #rings>7, H-acc>10, H-don>5	Followed zero rules	0.17
**24. **	**Glycine,N-[(3.alpha.,5.beta.,12.alpha)**	No; MW>500, MLOGP>4.15	No; MW>480, WLOGP>5.6, MR>130, #atoms>70	No; Rotors>10	No; WLOGP>5.88	No; MW>600, XLOGP3>5	Followed zero rules	0.17
**25. **	**Shatavarin - X**	No; MW>500, NorO>10, NHorOH>5	No; MW>480, MR>130, #atoms>70	No; Rotors>10, TPSA>140	No; TPSA>131.6	No; MW>600, TPSA>150, #rings>7, H-acc>10, H-don>5	Followed zero rules	0.17
**26. **	**Shatavarin-IV/Asparanin B**	No; MW>500, NorO>10, NHorOH>5	No; MW>480, MR>130, #atoms>70	No; TPSA>140	No; TPSA>131.6	No; MW>600, TPSA>150, #rings>7, H-acc>10, H-don>5	Followed zero rules	0.17
**27. **	**Reserpine**	No; MW>500, NorO>10	No; MW>480, MR>130, #atoms>70	Yes	Yes	No; MW>600	Followed two rules	0.17
**28. **	**Shatavarin-I**	No; MW>500, NorO>10, NHorOH>5	No; MW>480, WLOGP<-0.4, MR>130, #atoms>70	No; Rotors>10, TPSA>140	No; TPSA>131.6	No; MW>600, TPSA>150, #rings>7, H-acc>10, H-don>5	Followed zero rules	0.17
**29. **	**(R)-N-(1’-methoxycarbonyl-2’-phenylethyl)-4-hydroxybenzamide**	Yes	Yes	Yes	Yes	Yes	Followed all five rules	0.55
**30. **	**Asparanin-A**	No; MW>500, NorO>10, NHorOH>5	No; MW>480, MR>130, #atoms>70	No; TPSA>140	No; TPSA>131.6	No; MW>600, TPSA>150, #rings>7, H-acc>10, H-don>5	Followed zero rules	0.17

Names of the thirty metabolites selected for druglikeness analysis are in bold.

### Protein-ligand interactions

The identification and evaluation of binding site interaction of the ligands with the active pocket of the target receptor plays an important role, as it involves ligand modification during the lead optimization stage of drug discovery development (Abdul-Hammed et al., 2022). Based on the drug-likeness analysis, the top ten metabolites were selected for further interaction analysis with receptor proteins. These metabolites include 4-hydroxybenzoate, methylcoumarate, 2,6-dihydroxy-4-methoxyacetophenone, trans-p-coumaric acid, isoliensinine, neferine, eudesmic acid, lisinine, scoparone, and MCPHB (**Table 2**).

**Table 2:** Docking score, bonding interaction, and bond distance (Å) of the top selected metabolites.

Sr. No.	Metabolites	Docking score kcal/mol	Bond category	Bond type	Residues	Bond distance
**1**	**4-Hydroxybenzoate**	-14.726	Hydrophobic	Pi-Alkyl	ALA307	4.5
**2**	**Methylcoumarate**	-14.377	Hydrogen Bond	Conventional Hydrogen Bond	LEU503	3
			Hydrophobic	Alkyl	LEU304	5.2
			Hydrophobic	Pi-Alkyl	ALA307	4.9
**3**	**2,6-Dihydroxy-4-methoxyacetophenone**	-14.145	Hydrogen Bond	Carbon Hydrogen Bond	CYS463	3
			Hydrophobic	Pi-Alkyl	ALA307	5.1
**4**	**Trans-p-coumaric acid**	-14.069	Hydrogen Bond	Conventional Hydrogen Bond	HIS461	2
			Hydrogen Bond	Carbon Hydrogen Bond	CYS463	2.8
**5**	**Isoliensinine**	-14.048	Hydrogen Bond	Conventional Hydrogen Bond	ARG378	2.1
			Hydrogen Bond	Carbon Hydrogen Bond	HIS461	2.8
					GLN146	2.8
					HIS461	2.9
					ARG378	4.9
			Electrostatic	Pi-Cation	CYS463	4
			other	Pi-Sulfur	TYR122	4.5
			Hydrophobic	Pi-Pi Stacked	LEU304	5.2
					ALA307	4.9
			Hydrophobic	Alkyl	ILE464	5.3
					LEU109	4.7
			Hydrophobic	Pi-Alkyl	ILE376	4
					VAL135	4.7
					VAL150	5.1
					MET300	3.4
					PRO372	4.6
					ILE373	5.2
					ALA307	4.7
					ILE373	5.1
					ILE376	5.2
					ALA303	5.2
					LEU304	5
					ALA307	5.4
**6**	**Neferine**	-13.749	Hydrogen Bond	Conventional Hydrogen Bond	HIS461	2.5
			Hydrogen Bond	Carbon Hydrogen Bond	GLY457	2.7
					LEU503	2.9
					PRO455	2.9
					PRO455	2.9
			Hydrophobic	Pi-Sigma	ILE373	2.6
			Other	Pi-Sulfur	CYS463	3.2
			Hydrophobic	Alkyl	ALA307	3.7
					ILE373	4.3
					ILE376	5
					CYS463	5
					VAL135	3.5
					LEU143	4.5
					LYS147	4.5
					ILE373	4.5
					ILE376	4.5
			Hydrophobic	Pi-Alkyl	ILE373	4
					CYS463	4.1
					ALA307	3.5
					VAL135	4.8
					ALA303	4.7
					LEU304	4.9
					ILE376	4.8
**7**	**Eudesmic acid/3,4,5-Trimethoxybenzoic acid**	-13.255	Hydrophobic	Alkyl	ALA307	4
**8**	**Liensinine**	-13.243	Hydrogen Bond	Conventional Hydrogen Bond	PRO455	1.9
			Hydrogen Bond	Carbon Hydrogen Bond	LEU304	3
					SER311	2.6
			Other	Pi-Sulfur	CYS463	5.2
			Hydrophobic	Alkyl	ALA307	4.9
					ILE373	4.8
					CYS463	4.7
					ILE464	5.2
					ALA469	4.3
					LEU205	4.7
					LEU304	4.3
					CYS463	4.9
			Hydrophobic	Pi-Alkyl	TYR122	4.7
					PHE229	4.8
					HIS310	4.7
					PHE468	4.4
					ILE373	4.8
					ILE376	5.4
					ALA307	3.6
					LEU304	5.4
					ALA307	4.3
					CYS463	4.8
**9**	**Scoparone**	-13.205	Hydrogen Bond	Carbon Hydrogen Bond	CYS463	2.8
					LEU503	2.8
			Unfavorable	Unfavorable Acceptor-	HIS461	2.8
**10**	**(R)-N-(1’-methoxycarbonyl-2’-phenylethyl)-4-hydroxybenzamide**	-12.939	Hydrogen Bond	Carbon Hydrogen Bond	CYS463	2.4
			Hydrophobic	Alkyl	LEU304	5.2
			Hydrophobic	Pi-Alkyl	ALA307	4
					ILE373	5.4
**11**	**VNI standard**	-9.691	Hydrogen Bond	Carbon Hydrogen Bond	HIS461	2.7
			Hydrophobic	Amide-Pi Stacked	ILE464	5.5
			Hydrophobic	Pi-Alkyl	PHE229	4.3
					HIS310	4.3
					CYS463	5
					ILE464	4.7
					PHE504	4.9
					LEU304	5.4
					ALA307	4.4
					ILE373	5.1
					ILE376	4.4

Names of the top ten drug-like metabolites with the standard compound (VNI) are in bold.

The standard metabolite VNI (**Supplementary Figures S2A–C**) formed one carbon-hydrogen bond with HIS461, one amino-pi-stacked bond with ILE464, and nine pi-alkyl bonds with PHE229, HIS310, CYS463, ILE464, PHE504, LEU304, ALA307, ILE373, and ILE376. This binding is favorable for CYP51 inhibition as it hinders the activity of HIS461, which acts as an acidic and basic catalyst in the active site (Tiwari et al., 2018).

**Figure 2:**
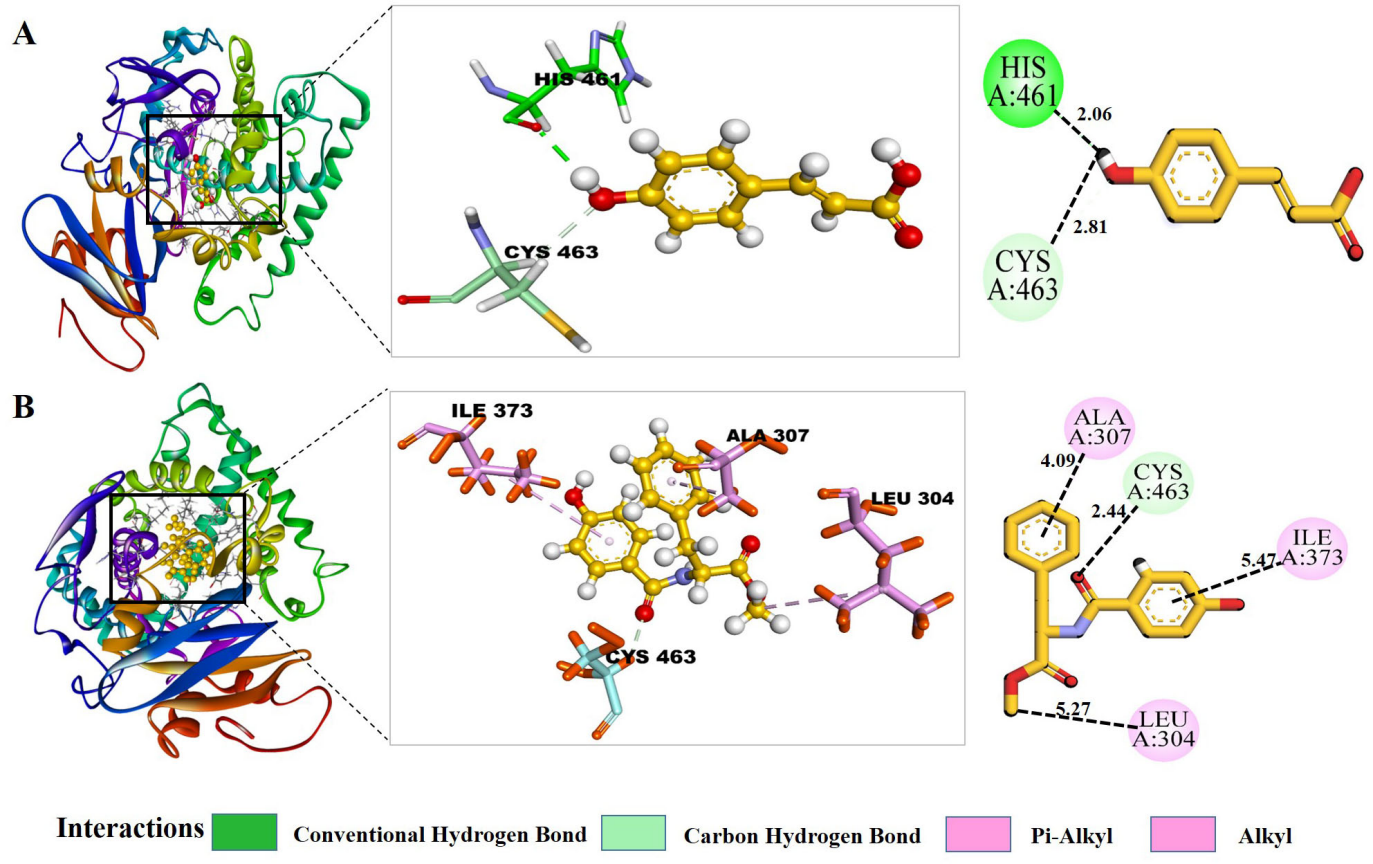
Protein-Ligand Interaction analysis of trans-p-coumaric acid and MCPHB; **(A)** 2D and 3D interactions of trans-p-coumaric acid and **(B)** 2D and 3D interactions of MCPHB in the binding pocket of target protein CYP51.

Trans-p-coumaric acid (**Figure 2A**) formed two hydrogen bond interactions with residues HIS461 and CYS463 with a bond distance of 2.0Å and 2.8Å. These two important residues are highly conserved in the active site of the target protein and involve catalytic enzymatic activity. Disturbing this activity results in the inhibition of ergosterol synthesis that leads to stopping fungal cell growth. This metabolite has previously been identified to exhibit antimicrobial, antioxidant, antitumor, and anti-inflammatory properties. This provides a strong foundation for further investigation into its predicted antifungal potential (Tungmunnithum et al., 2018).

Surprisingly, the three bisbenzylisoquinoline alkaloids (Neferine, Liensinine, and Isoliensinine) have formerly been shortlisted as leads in our previous publication (Shah et al., 2024). In the previous study, they were isolated from *N. nucifera Gaertn* and presented dual-active behavior against the main protein and spike glycoprotein of COVID-19. In the current study, these metabolites formed various hydrogen bonds and hydrophobic bonds with different bond lengths with our target protein CYP51 (**Table 2**). Isoliensinine formed one conventional hydrogen bond with ARG378 (2.1°C) and four carbon-hydrogen bonds with HIS461 (2.8°C), GLN146 (2.8°C), HIS461 (2.9°C), and ARG378 (4.9°C) residues. It also forms one pi-cation bond with CYS463 (4.0 Å), one pi sulfur bond with TYR122 (4.5 Å), two pi-pi stacked bonds with LEU304 (5.2 Å) and ALA307 (4.9 Å), eight alkyl bonds with ILE464 (5.3 Å), LEU109 (4.7 Å), ILE376 (4.0 Å), VAL135 (4.7 Å), VAL150 (5.1 Å), MET300 (3.4 Å), PRO372 (4.6 Å) and ILE373 (5.2 Å), and six pi-alkyl bonds with ALA307 (4.7 Å), ILE373 (5.1 Å), ILE376 (5.2 Å), ALA303 (5.2 Å), LEU304 (5.0 Å), and ALA307 (5.4 Å) (**Supplementary Figures S3A–C**). Tyr122 residues in the CYP51 family of target proteins form hydrogen bonds with the heme ring, which is important for the stability of protein structure, but binding with isoliensinine inhibitors often disrupts this hydrogen bond formation in structure, resulting in the death of fungi (Hargrove et al., 2015).

Liensinine formed a conventional hydrogen bond with the PRO455 residue at a shortest bond distance of 1.9Å that indicated a strong binding with the target protein. Additionally, it formed two carbon-hydrogen bonds with LEU304 (3.0Å) and SER311 (2.6Å). Liensinine also established other interactions, including one pi-sulfur bond with CYS463 (5.2Å), seven alkyl bonds with ALA307 (4.9Å), ILE373 (4.8Å), CYS463 (4.7Å), ILE464 (5.2Å), ALA469 (4.3Å), LEU205 (4.7Å), and LEU304 (4.3Å), and eleven pi-alkyl bonds with CYS463 (4.9Å), TYR122 (4.7Å), PHE229 (4.8Å), HIS310 (4.7Å), PHE468 (4.4Å), ILE373 (4.8Å), ILE376 (5.4Å), ALA307 (3.6Å), LEU304 (5.4Å), ALA307 (4.3Å), and CYS463 (4.8Å) (**Supplementary Figures S4A–C**).

Neferine forms one conventional hydrogen bond with HIS461 (2.5Å) and four carbon-hydrogen bonds with GLY457 (2.7Å), LEU503 (2.9Å), and PRO455 (two at 2.9Å). It also forms one pi-sigma bond with ILE373 (2.6Å) and one pi-sulfur bond with CYS463 (3.2Å). Additionally, Neferine forms nine alkyl bonds with ALA307 (3.7Å), ILE373 (4.3Å), ILE376 (5.0Å), CYS463 (5.0Å), VAL135 (3.5Å), LEU143 (4.5Å), LYS147 (4.5Å), and two more with ILE373 (4.5Å) and ILE376 (4.5Å). It also forms seven pi-alkyl bonds with ILE373 (4.0Å), CYS463 (4.1Å), ALA307 (3.5Å), VAL135 (4.8Å), ALA303 (4.7Å), LEU304 (4.9Å), and LEU376 (4.8Å) (**Supplementary Figures S5A–C**).

These three metabolites belong to the class of bisbenzylisoquinoline alkaloids, which are significant due to their highest binding affinities with the target protein. The strong affinity of these metabolites for the active site of the target protein is evidenced by the extensive number of bonds formed with various residues, namely ILE376, LEU304, CYS463, ALA307, HIS461, and ILE464. Consequently, these bisbenzylisoquinoline alkaloids show potential as effective inhibitors for the target protein. Previous studies have demonstrated that bisbenzylisoquinoline alkaloids possess a diverse range of pharmacological effects. According to Zhu et al. (2016), these metabolites exhibit significant anti-depressant, anti-arrhythmic, anti-pulmonary edema, and anti-HIV properties (Zhu et al., 2016). Additionally, research by Liu et al. (2021) has identified potent anti-cancer activity among these alkaloids (Liu et al., 2021). This broad spectrum of pharmacological actions represents the potential therapeutic applications of bisbenzylisoquinoline alkaloids across various medical conditions.

**Figure 3:**
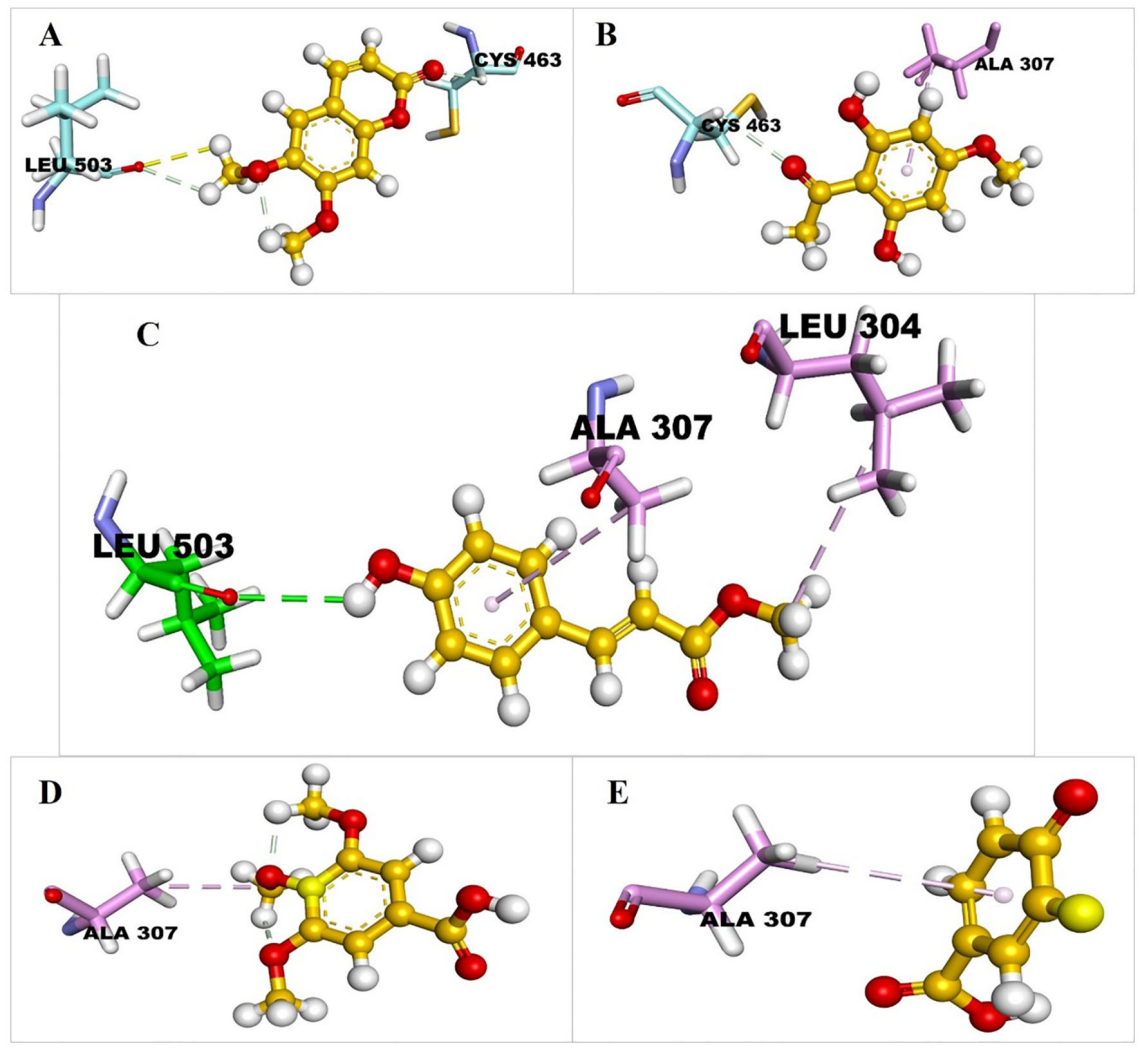
3D interaction profiles of **(A)** 1 Scoparone, **(B)** 3 2,6-Dihydroxy-4-methoxyacetophenone, **(C)** 2 Methylcoumarate, **(D)** 4 Eudesmic acid, and **(E)** 5 4 Hydroxybenzoate in the binding pocket of CYP51.

MCPHB (**Figure 2B**) and Scoparone (**Figure 3A**) metabolites formed one carbon-hydrogen bond with the same residue, CYS463, but with different bond lengths of 2.44 Å and 2.80 Å, respectively. 2,6-Dihydroxy-4-methoxyacetophenone (**Figure 3B**) and methylcoumarate (**Figure 3C**) metabolites formed one hydrogen bond with LEU503 and CYS463 with a bond distance of 3.05Å and 3.07Å, respectively (**Table 2**). Eudesmic acid (3,4,5-trimethoxybenzoic acid) (**Figure 3D**) formed one alkyl bond with ALA307. It showed the highest antibacterial activity (Bisignano et al., 2000) and antioxidant properties. It possesses various biological and pharmacological effects, such as triggering the self-destruction of cancer cells, neutralizing free radicals, and disrupting the signaling processes that involve calcium ions and reactive oxygen species (Atewolara-Odule et al., 2020). 4-hydroxybenzoate (**Figure 3E**) has not formed any interactions with the H bonds, but it shows interactions with the pi-alkyl bond with the residue of ALA307.

Catalytically important active site residues LEU503, HIS461, ARG378, and PRO455 that involve the formation of conventional hydrogen bonds in protein-ligand interaction, along with the residues that formcarbon-hydrogen bonds, i.e, CYS463, HIS461, GLN146, ARG378, GLY457, LEU503, PRO455, LEU304, and SER311, were prominent in the interaction profiles of the studied metabolites. The residues involved in hydrophobic interactions with ligands are ALA307, LEU304, ILE464, LEU109, ILE376, VAL135, VAL150, MET300, PRO372, ILE373, ALA303, CYS463, VAL135, LEU143, LYS147, ALA307, ALA469, LEU205, TYR122, PHE229, HIS310, PHE468, HIS461, and PHE504. The maximum number of interactions with the hydrophobic residues was observed with isoliensinine, neferine, and liensinine metabolites. Hydrophobic interaction was prominent in the interaction profile of most of the inhibitors, highlighting the significance of hydrophobic residues in the active site of the CYP51 target protein (**Table 2**). The most favorable profiles were observed with trans-p-coumaric acid and MCPHB, which were selected for detailed analysis in the further sections. In **Table 2**, docking scores were reported with three decimal places for precision, while bond lengths were recorded with one decimal place, following standard conventions in the literature.

### MD simulation

Among the top 10 drug-like compounds (following all rules), 2 metabolites for MD were chosen for structural uniqueness, full compliance with Lipinski’s rule, and potential as small, promising drug molecules. Standard VNI was also simulated alongside. To derive rational conformational samplings of the selected leads, their complexes with the CYP51 enzyme were subjected to 100 ns MD simulations.

### Root-mean-square deviations

**Figure 4:**
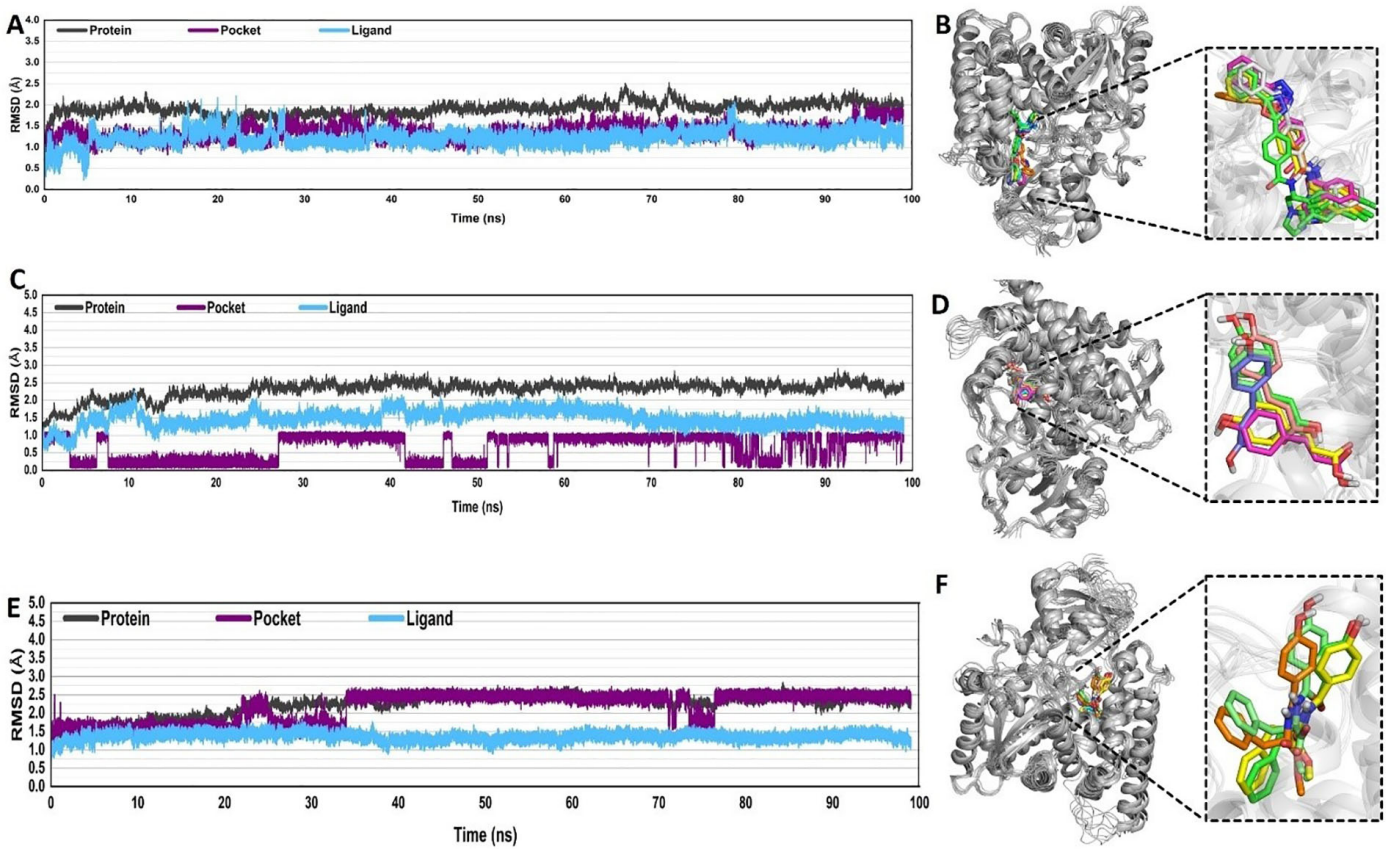
Root mean square deviation (RMSD) analysis for **(A, B)** co-crystallized VNI, **(C, D)** trans-p-coumaric acid, and **(E, F)** MCPHB complexes. **(A, C, E)** RMSD plots showing the time evolution of the protein, pocket, and ligand across 100 ns of MD simulations. RMSD values for protein (black), pocket (purple), and ligand (blue) were monitored to assess structural stability. **(B, D, F)** 3D representations of the protein-ligand complexes, with insets highlighting different conformations of the ligands bound in the protein pocket.

**Figure 5:**
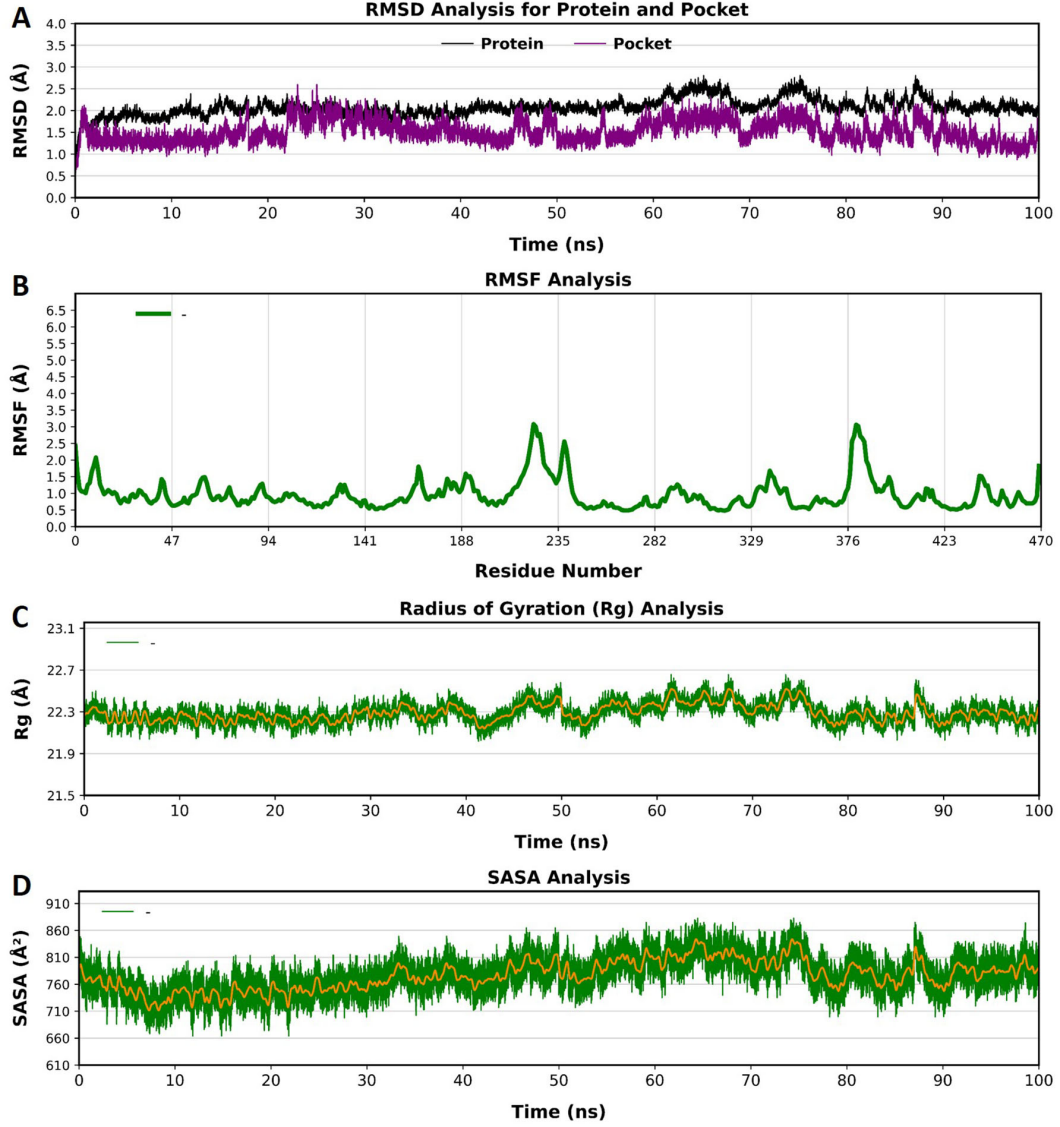
Root mean square deviation (RMSD) **(A)**, root-mean-square fluctuation (RMSF) **(B)**, radius of gyration (Rg) **(C)**, and solvent-accessible surface area (SASA) **(D)** analyses of apoprotein (CYP51). The x-axis represents residue numbers/time in nanoseconds (ns), while the y-axis indicates the RMSF, Rg, or SASA values (in Å).

To assess the stability of MD simulations, RMSDs of backbone atoms in the selected ligands with respect to the crystal structures were computed as a function of the simulated time (**Figure 4**). MD simulation of non-ligand-bonded protein, apoprotein (CYP51), was also performed for the same duration of time (100 ns) using the same parameters and protocol as that used for the PN-LIG (protein-ligand) complex (**Figure 5**).

**Figure 6:**
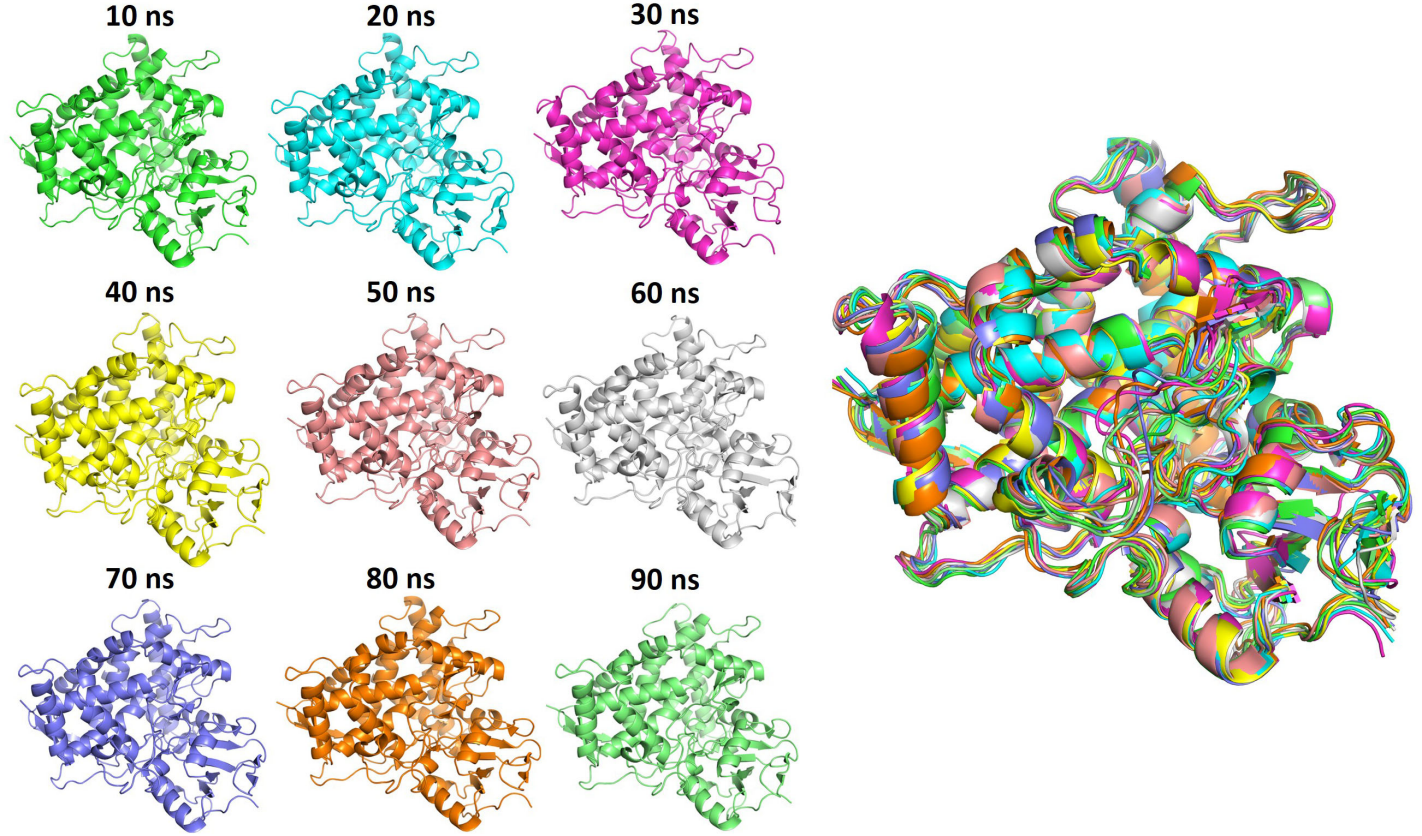
MD snapshots of the apoprotein were captured at different time intervals (left panel). Superimposed MD snapshots highlighting the similarity between poses observed during the MD analysis (right panel).

The RMSD plot shows that the apoprotein reached stability after approximately 20 ns, with minor fluctuations observed throughout the simulation (**Figure 5A**). The overall RMSD values remained within an acceptable range. The binding pocket also exhibited a similar trend with slightly lower deviations. These results suggest that both the protein and pocket maintained its structural integrity and stability during simulation. This can be further illustrated by the snapshots taken at different time intervals during MD simulations and the superimposition of these snapshots (**Figure 6**).

For the VNI-protein complex, the average RMSD values for both the protein and ligand remained below 2.5 Å throughout the simulation. While minor deviations occurred in the VNI conformation, these fluctuations remained within an acceptable range, which indicated the structural integrity of the co-crystalized complex over time (**Figures 4A, B**).

In the case of the trans-p-coumaric acid complex, the RMSD values for the protein remained stable, but the binding pocket displayed some conformational instability beyond 50 ns, with transient deviations in RMSD that exceeded 3 Å. These fluctuations reflect flexibility in the binding pocket, which may influence the strength or stability of the ligand binding. However, the system re-equilibrated and remained stable over extended timescales (**Figures 4C, D**).

The MCPHB complex exhibited the most stable binding, with average RMSD values for both protein and ligand remaining around 2 Å throughout the 100 ns simulation. The pocket also demonstrated minimal fluctuations, indicating a strong and stable interaction between the ligand and the protein binding site (**Figures 4E, F**). Overall, all three complexes reached equilibrium after some initial fluctuations, with RMSD values staying below 3 Å. This suggests that the systems are sufficiently stable for further analysis.

### Root-mean-square fluctuations

**Figure 7:**
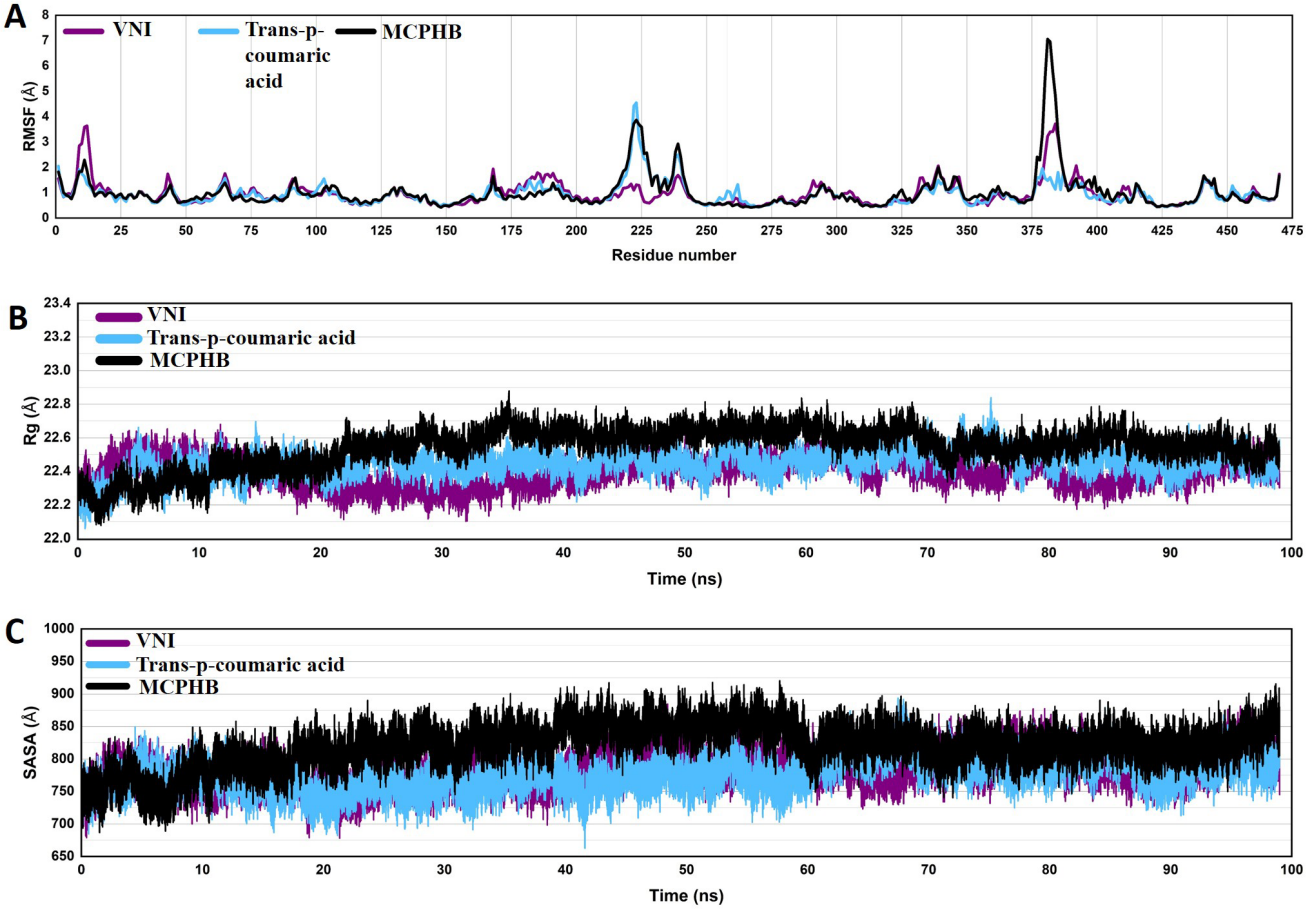
Root-mean-square fluctuation (RMSF) **(A)**, radius of gyration (Rg) **(B)**, and solvent-accessible surface area (SASA) **(C)** analyses of protein (CYP51) bound with VNI (purple), trans-p-coumaric acid (blue), and MCPHB (black) over the 100 ns simulation period. The x-axis represents residue numbers/time in nanoseconds (ns), while the y-axis indicates the RMSF, Rg, or SASA values (in Å).

To evaluate and compare the structural flexibility of the protein in the presence of different ligands, we computed the root-mean-square fluctuations (RMSF) of the C-alpha atoms across the three systems of each selected ligand (**Figure 7A**). The RMSF analysis demonstrates a largely similar fluctuation pattern for all three complexes. This similarity suggests that the protein maintains consistent conformational dynamics, whether unbound (**Figure 5B**) or bound to any of the three ligands (**Figure 7A**).

The RMSF profile of the apoprotein (unbound state) highlights regions with high flexibility, particularly around residues 188, 235, and 376, which show prominent peaks. These fluctuations are primarily located in loop regions, whereas the core residues remain stable. These results indicate that the apoprotein retains its overall structural framework during the simulation.

In the bound state, the majority of residues show RMSF values below 2 Å, indicating high structural stability in these regions. Notably, significant fluctuations occur in a few specific regions, with the highest peaks observed around residues 225 and 375, regardless of the ligand bound. These peaks correspond to flexible loops or regions distant from the binding site, which are generally expected to exhibit higher mobility.

The comparable RMSF values across systems further confirm that ligand binding does not introduce significant conformational disruption in the protein. Such results indicate that the system remains folded and stable throughout the simulation. These findings highlight the stability of the protein-ligand complexes in solution, suggesting that the ligands (trans-p-coumaric acid and MCPHB) do not negatively impact the protein’s structural integrity or folding over the simulation period.

### Radius of gyration

To evaluate the impact of ligand binding on the structural compactness of the protein, the radius of gyration (Rg) was monitored over 100 nanoseconds of simulation for the three ligand-bound systems (**Figure 7B**). The Rg values for all systems remained stable throughout the simulation, indicating that the overall protein structure maintained its compactness in bound and unbound states.

The Rg plot demonstrates that the apoprotein remains compact throughout the simulation (**Figure 5C**), with values fluctuating within a narrow range (~22.3 Å). Overall, the results indicated no significant expansion or collapse of the protein structure.

For the VNI-protein complex, the Rg values fluctuated between 22.2 Å and 22.6 Å (**Figure 7B**). The trans-p-coumaric acid complex exhibited similar behavior, with Rg values mostly between 22.3 Å and 22.7 Å. The MCPHB complex showed the highest Rg range, fluctuating between 22.4 Å and 23.0 Å (**Figure 7B**). All these variations remained within acceptable limits and indicated stable structural integrity. This stability implies that the protein retains its original folded state, which is essential for maintaining the architecture required for effective ligand interaction.

### Solvent-accessible surface area

To investigate the effect of ligand binding on the solvent accessibility of the protein, the solvent-accessible surface area (SASA) was monitored throughout 100 nanoseconds of molecular dynamics simulation for the three systems (**Figure 7C**). SASA provides insights into the degree of protein surface exposure to the solvent, reflecting possible structural changes or ligand-induced compactness variations.

For the apoprotein (unbound state), SASA values remain relatively stable, fluctuating between 760 and 860 Å² (**Figure 5D**). These results suggest minor conformational changes during the solvent exposure.

For the VNI-protein complex, SASA fluctuated primarily between 700 Å² and 800 Å². These values are in the acceptable range and indicate stable solvent accessibility throughout the simulation. Similarly, the trans-p-coumaric acid complex exhibited SASA values within a comparable range, though it showed slightly more frequent dips in surface exposure, indicating minor fluctuations in protein conformation. The MCPHB complex maintained the highest SASA values, fluctuating between 750 Å² and 900 Å², suggesting that this system had the largest surface area exposed to the solvent. The consistent SASA patterns observed across the three complexes reflect that ligand binding did not cause significant changes in the protein’s overall folding or surface exposure.

### Dynamic Cross-Correlation Map

**Figure 8:**
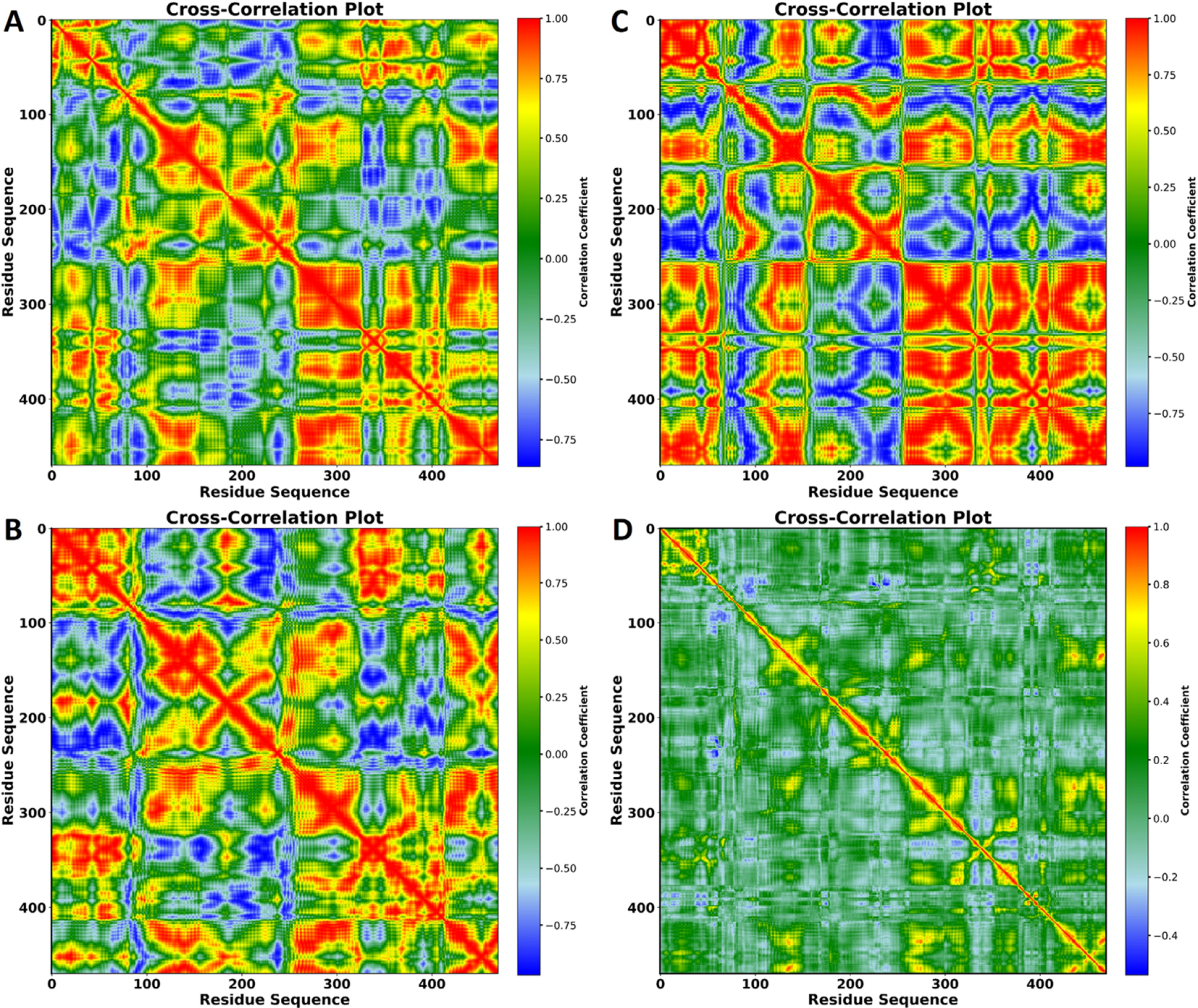
Cross-correlation plots of residue motions in the protein in complexes with **(A)** VNI, **(B)** trans-p-coumaric acid, **(C)** MCPHB, and **(D)** Crosscorrelation plot of residue motions in the apoprotein. The x- and y-axes represent the residue sequence, and the color scale on the right indicates the correlation coefficients, ranging from -1 (anti-correlated, blue) to +1 (positively correlated, red). These plots show the degree of correlated and anti-correlated motions between residues throughout the 100 ns MD simulation.

To investigate the impact of ligand binding on the internal dynamics of the protein, cross-correlation maps were computed for the selected three ligand-protein systems (**Figure 8**). The diagonal elements of these maps represent intra-residue motions, indicating the movement of individual residues relative to themselves. In contrast, the off-diagonal regions reflect inter-residue motions, showing the relative movements between different residues.

The color coding reveals the degree of correlation between residue pairs. Red regions represent highly positive correlations, indicating synchronized motion between residues, while blue areas reflect strong anti-correlations, signifying that residues move in opposite directions. The cross-correlation analysis shows that ligand binding influences the internal dynamics of the protein by modifying the correlated and anti-correlated movements of specific regions (**Figures 8A–C**). Although each ligand introduces unique patterns of motion, the preservation of positive correlations in key regions highlights the stability of these interactions, which could be crucial for maintaining the functional conformation of the protein. On the other hand, no significant correlation or anti-correlation was observed in the DCCM results of the apoprotein, indicating its stable confirmation throughout the simulation (**Figure 8D**).

### Principal component analysis and free energy landscape

**Figure 9:**
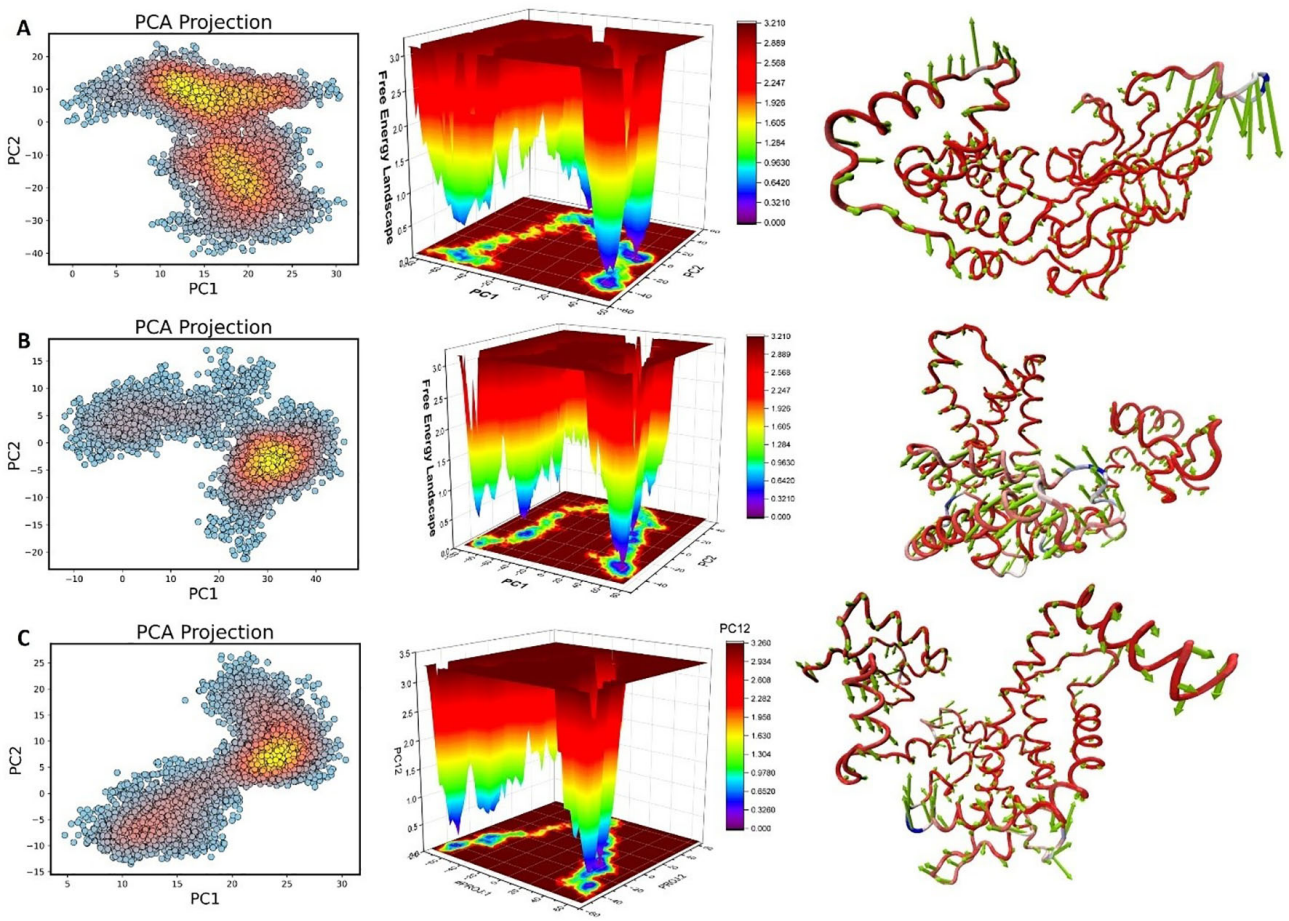
Principal Component Analysis (PCA) and Free Energy Landscape (FEL) representations for the dynamics of the protein-ligand complexes with **(A)** VNI, **(B)** trans-p-coumaric acid, and **(C)** MCPHB. Left panels: PCA projections show the movement trajectories along the first two principal components (PC1 and PC2). Middle panels: FELs reveal energy variations across different conformational states, with red indicating high-energy states and blue indicating low-energy (stable) states. Right panels: Structural representations highlight conformational variations throughout the simulation for each complex. Thicker ribbons indicate areas of higher mobility, and thinner ribbons indicate more rigid regions.

**Figure 10:**
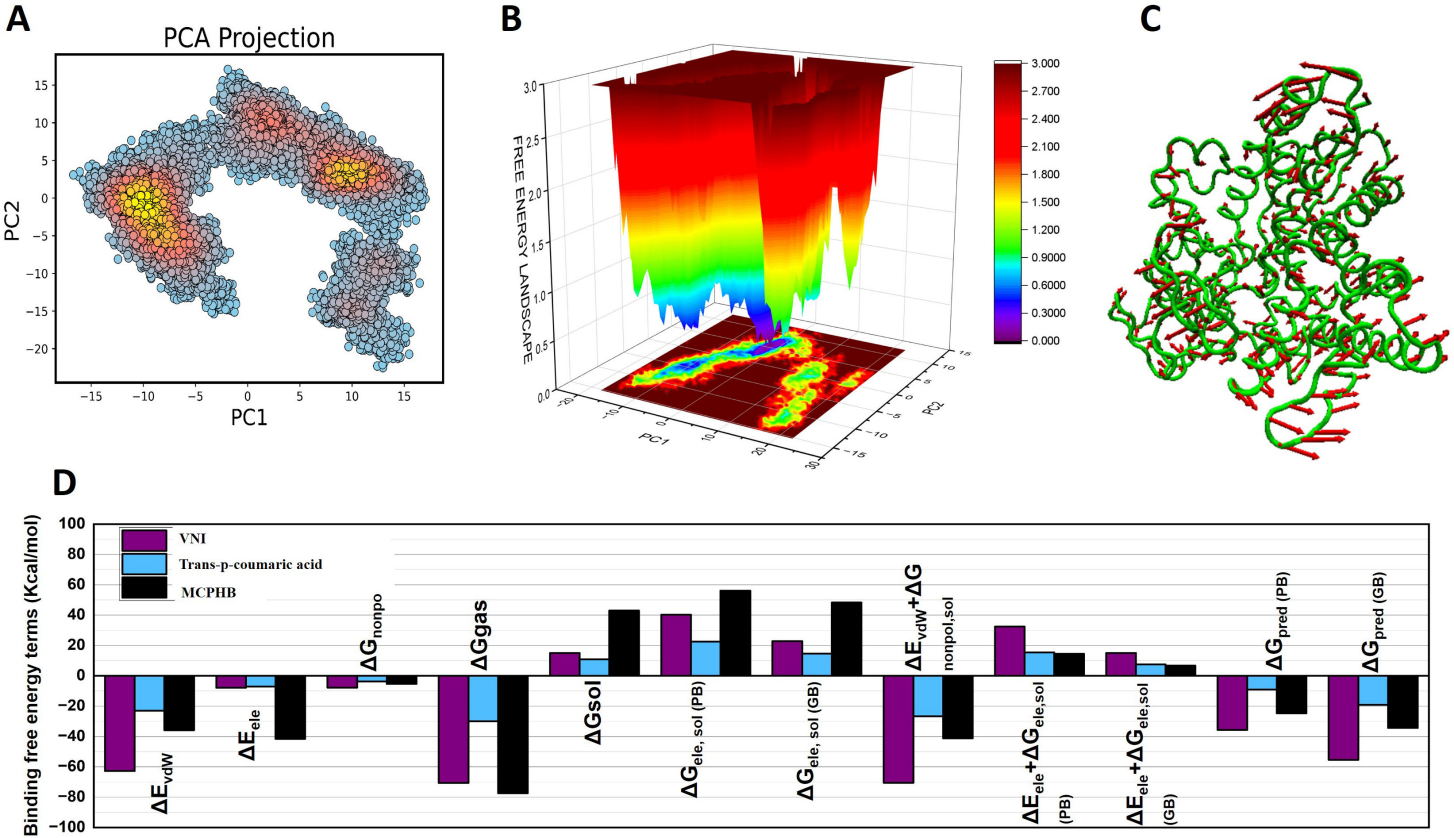
Principal Component Analysis (PCA) **(A)** Free Energy Landscape (FEL) **(B)** and Principal Component (PC1) **(C)** of the apoprotein during the 100 simulations. Structural representations highlight conformational variations throughout the simulation for apoprotein. Thicker ribbons indicate areas of higher mobility, and thinner ribbons indicate more rigid regions. Binding free energy decomposition analysis **(D)** for the protein-ligand complexes with VNI (purple), trans-p-coumaric acid (blue), and MCPHB (black). The y-axis represents binding free energy in kcal/mol for each term. Key components shown include van der Waals energy (ΔE_vdW_), electrostatic energy (ΔE_ele_), gas-phase energy (ΔG_gas_), and solvation energy (ΔG_sol_). The graph also shows predictions of binding free energy with both Poisson-Boltzmann (ΔG_pred_(PB)) and Generalized Born (ΔG_pred_(GB)) methods.

Principal Component Analysis (PCA) identified key motion patterns in the protein-ligand systems (**Figures 9**, **10**). The eigenvalues reflect the variance in movement, where higher values correspond to larger conformational shifts. The PCA projections reveal different clustering patterns, indicating distinct dynamics for each complex.

The VNI-protein complex (**Figure 9A**) shows tight clustering, which suggests minimal conformational shifts. The FEL indicates deep basins (blue regions), which represent stable low-energy states. This implies that VNI forms a stable interaction with the protein and limits structural variability. The trans-p-coumaric acid complex (**Figure 9B**) shows more scattered clusters, reflecting greater conformational flexibility. The FEL highlights broader low-energy regions with small high-energy peaks, indicating that this ligand induces more dynamic movements while maintaining some stable states. The MCPHB complex (**Figure 9C**) shows intermediate clustering with multiple low-energy basins. The FEL suggests smooth transitions between stable states, indicating dynamic yet stable interactions.

For the apoprotein, the PCA plot (**Figure 10A**) shows how the protein explores different conformations. The clusters of points represent stable states, while the spread of points indicates transitions between these states. The densest regions (yellow) highlight the most frequent conformations. The presence of multiple low-energy basins suggests that the apoprotein adopts different stable conformations during the simulation (**Figure 10B**). Structural representations in **Figure 10C** highlight conformational variations throughout the simulation for apoprotein. Thicker ribbons indicate areas of higher mobility, and thinner ribbons indicate more rigid regions.

The overall results reflect moderate flexibility and adaptable binding while preserving structural stability.

### Binding free energy

This free energy decomposition reveals how various energetic contributions affect the binding stability of the three ligand-protein systems. Each ligand displays a unique balance of van der Waals, electrostatic, and solvation energies, which influence the overall interaction strength. The VNI-protein complex shows strong van der Waals forces (ΔE_vdW_) and stable electrostatic energy (ΔE_ele_). These forces, along with favorable solvation energy, contribute to highly negative predicted binding energies (ΔG_pred_) in both Poisson-Boltzmann (PB) and Generalized Born (GB) models.This suggests that VNI forms a stable interaction with the protein (**Figure 10D**).

The trans-p-coumaric acid complex relies more on solvation energy (ΔG_sol_) for stability. Although its van der Waals contributions are lower compared to the other ligands, favorable solvent interactions help maintain a stable complex. The overall predicted binding energy is less negative than that of VNI, indicating a weaker binding affinity (**Figure 10D**).

The MCPHB complex achieves stability through a balanced contribution of van der Waals and electrostatic forces. Its solvation energy also supports the stability of the complex. The predicted binding energy remains highly negative, suggesting that MCPHB binds effectively to the protein while maintaining stable interactions with the solvent. In summary, VNI depends heavily on van der Waals forces, trans-p-coumaric acid benefits more from solvent interactions, and MCPHB achieves a balanced interaction through multiple energy components. All three systems maintain sufficiently stable binding for potential biological function (**Figure 10D**).

### Per-residue energy decomposition

The per-residue energy decomposition was calculated for the lead compounds in comparison to the cocrystalized ligand (CCL), namely VNI, to gain insight into the contribution of each residue of the binding pocket to the BFE of each complex. The contribution of each residue was further broken down into van der Waals, electrostatic, polar solvation, and non-polar solvation.

The total energy decomposition of trans-p-coumaric acid shown in **Supplementary Table S3** illustrated that Val86, Ile415, Gln97, and Cys414 were observed as hotspot residues with major contributions in the binding affinity due to their strong vdW interactions as well as balanced polar and non-polar solvation. The residues Leu94 and Met257 slightly contributed with minimal electrostatic effects. However, destabilizing factors were also observed due to the major contribution of polar solvation rather than non-polar solvation. Notably, pronounced flexibility and polar interaction were noted at the Gln97 and Lys98 residues due to high variation in electrostatic as well as solvation contribution.

The energy decomposition of residues for the MCPHB complex highlighted in **Supplementary Table S4** presented that Tyr87, His412, Ala69, and Val86 made a significant contribution in stabilization due to strong vdW and electrostatic interactions. However, the Lys98 residue was involved in destabilizing effects due to high polar solvation, as observed in the trans-p-coumaric acid complex. Moreover, both Cys414 and Ile415 residues made a balanced contribution in vdW and electrostatic interactions with polar solvation effects. **Supplementary Table S5** represents the per-residue energy decomposition of binding residues of the VNI complex, revealing the highest total energy values due to the maximum contribution of hydrophobic interactions by the following residues: Leu454, Leu76, Phe185, and Ala258. These residues were mainly involved in significant vdW interactions as well as electrostatic interactions with Tyr73 and Cys414, signifying the importance of hydrophobic interactions and electrostatic stabilization with the VNI. Simultaneously, polar solvation effects were observed by the Lys45 residue playing a role in the destabilization of the complex.

Overall, the per-residue energy decomposition analysis showed that Val86, Tyr73, and Leu76 were crucial residues of binding pockets across all ligands, majorly involved in hydrophobic interactions with high stabilizing contributions. Additionally, the polar solvation effects could be mitigated by the incorporation of non-polar or aromatic functional groups.

### Essential pharmacophores of CYP51

**Figure 11:**
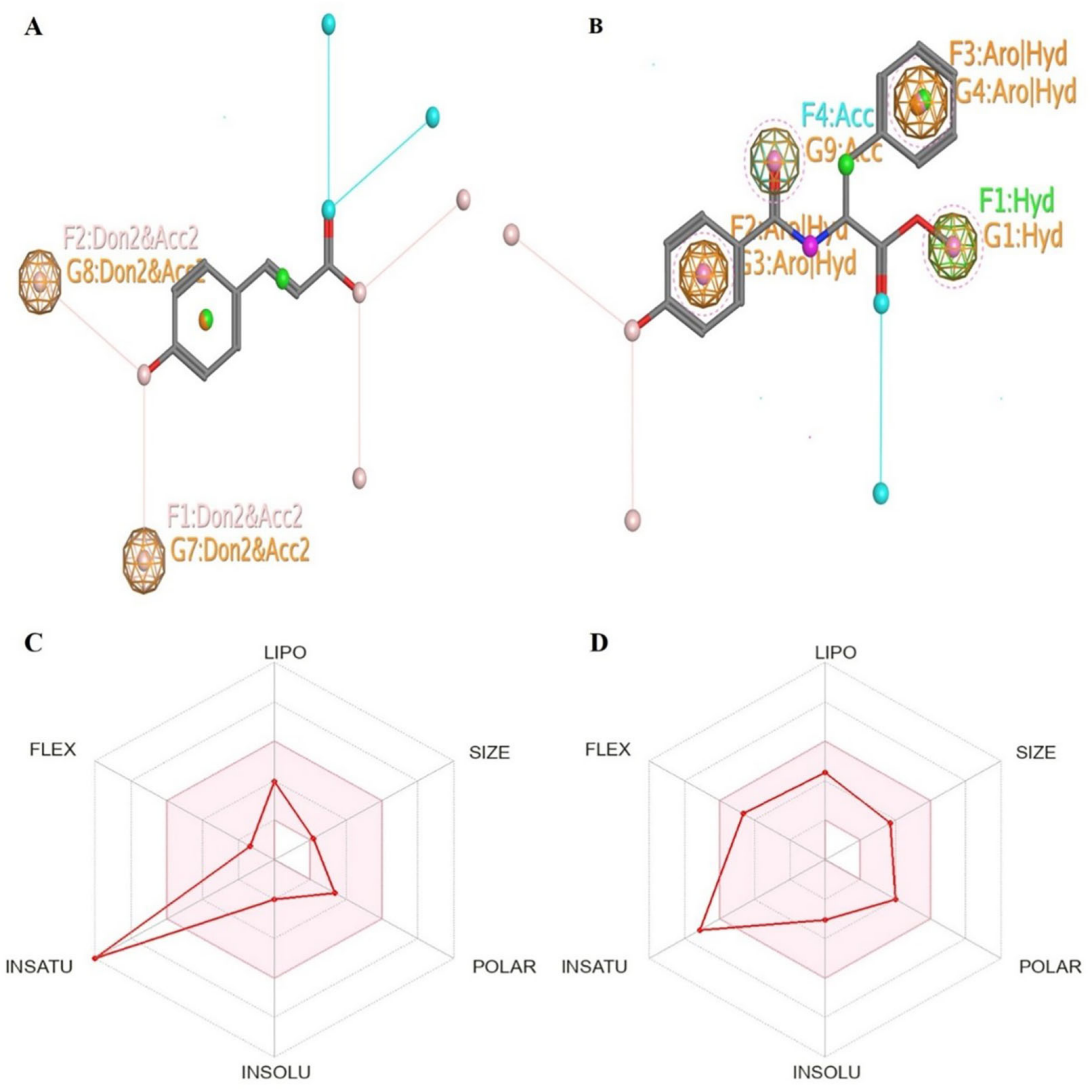
Essential pharmacophoric features of **(A)** trans-p-coumaric acid and **(B)** MCPHB for the inhibition of target protein. With variable radii, the identified features are G1, G2, G4, G7, and G8, and G9, with the descriptors of Hyd, Hyd|Aro, Don2&Acc2, and Acc indicating the hydrophobic, aromatic, and hydrogen bond donor-acceptor sites, respectively. Bioavailability Radar graphs by the SwissADME platform for **(C)** trans-p-coumaric acid and **(D)** MCPHB.

This analysis aims to find the specific pharmacophore patterns of the standard VNI (**Supplementary Figure S6**) and the 2 metabolites (trans-p-coumaric acid, MCPHB) of the Ayurvedic medicinal plant library (**Figures 11A, B**). This method maximizes the ability of these metabolites to bind and effectively inhibit the CYP51 target protein, hence increasing their potential as drugs.

These metabolites offer some distinct interactions that could potentially enhance the selectivity and potency of the drugs by offering other interaction points. The features labeled as G7 and G8 in trans-p-coumaric acid have the descriptor “Don2&Acc2” that form bonds with residues CYS463 and HIS461 (**Figures 11A**, **2A**) and G9 in MCPHB (**Figures 11B**, **2B**) “Acc” with residues CYS463, indicating the presence of hydrogen bond acceptor sites that are important for forming specific interactions with hydrogen bond donors within the active site of CYP51. The radius of G7, G8, and G9 is 0.50, respectively. These features emphasize the significance of hydrogen bond interactions forming C-C and C-H bonds that contribute to structural stability and are essential for binding to the target protein. The presence of “Hyd” in G1 with residue LEU304 and ““Hyd|Aro” in G3 with ALA307 and G4 with ILE373 residues (**Figure 2B**) suggests a region in the metabolites that can participate in both hydrophobic interactions and π-π stacking interactions with radii of 0.50 in both G1 and G3, and 0.55 in G4, offering a dual role in enhancing the binding affinity with the target protein. Comparatively, in the pharmacophoric profile of VNI, G9, G10, and G11 with descriptor “ACC2” and residues ILE464, CYS463, and ILE376; G12 and G13 with descriptor “ACC” and residues HIS4612 and ILE373, indicate the critical hydrogen bond acceptor features for the inhibition of CYP51 (**Supplementary Figure S6**). Furthermore, the “Hyd|Aro” feature in G6 with residues LEU304 and ALA307 offers a dual role in enhancing the binding affinity with the target protein.

By identifying these pharmacophoric properties, we can comprehend the multifaceted connection between the metabolites and their biological target. This emphasizes the importance of each interaction site and spatial arrangement in obtaining the intended therapeutic effect.

### Bioavailability score analysis

The six physicochemical properties that predict the bioavailability of the metabolite are LIPO (lipophilicity), SIZE, POLAR (polarity), INSOL (insolubility), INSATU (unsaturation), and FLEX (flexibility), respectively. The optimal range of physicochemical properties includes lipophilicity: XLOGP3 between −0.7 and +5.0, size: MW between 150 and 500 g/mol, polarity: TPSA between 20 and 130 Å^2^, solubility: log S not higher than 6, saturation: fraction of carbons in the sp^3^ hybridization not less than 0.25, and flexibility: no more than 9 rotatable bonds (Daina et al., 2017) (**Supplementary Table S6**). It should be mentioned here that an algorithmic value of the bioavailability score is also taken into account from the SwissADME webserver. These values have been mentioned in **Table 1** for the top 30 compounds and discussed under the section on drug-likeness and physicochemical properties.

Lipophilicity (logP) is a crucial parameter in determining the physicochemical properties of drugs. It measures a metabolism’s ability to stay in the aqueous phase, influencing its transport across membrane barriers. Lipophilicity also describes the intermolecular forces between the solute and solvent, which include various types of interactions such as ion-dipole interactions, charge transfer interactions, hydrogen bonds, ion-ion interactions, hydrophobic bonds, and van der Waals interactions. These interactions collectively impact the drug’s absorption, distribution, metabolism, and excretion (ADME) properties (Lewis et al., 2008; Pliška et al., 2008). ADME and toxicity profiles will be addressed in subsequent sections of this study. The optimal value of lipophilicity (XLOGP3) is between −0.7 and +5.0, and the MW of the metabolite is between 150 and 500 g/mol. All the metabolites, including trans-p-coumaric acid, MCPHB, and isoliensinine, lie in the optimal range of lipophilicity and molecular size of the metabolite (**Supplementary Table S6**). The polarity of metabolites describes the ability to cross the cell membrane. The optimal range of polarity (TPSA) is between 20 and 130 Å^2^. All our top metabolites follow the optimal range of polarity. Different metabolites show different solubility values (LogS). Metabolites with solubility values of 0 and above are extremely soluble; those in the range of 0 to −2 are soluble; those in the range of −2 to −4 are sparingly soluble; and those metabolites below −4 are insoluble (Chen et al., 2018). The solubility values of the metabolites isoliensinine, neferine, and liensinine are below −4 and are insoluble, and other metabolites are in the range of −2 to −4 and are sparingly soluble. The saturation of metabolites is the fraction of carbons in the sp^3^ hybridization that should not be less than 0.25. The metabolites isoliensinine, neferine, and liensinine are being Fsp3 hybridized. The flexibility of the metabolite shows the number of rotatable bonds. It should have no more than nine rotatable bonds. Most of our top metabolites, including trans-p-coumaric acid and MCPHB, followed the optimal range of rotatable bonds (**Figures 11C, D**). Overall, the metabolites, specifically methylcoumarate, 2,6-dihydroxy-4-methoxyacetophenone, trans-p-coumaric acid, isoliensinine, eudesmic acid, and MCPHB, were found to possess optimal bioavailability characteristics. This implies that they are likely to be efficiently absorbed, distributed, metabolized, and excreted within the body, maximizing their therapeutic potential and effectiveness as drug candidates. These characteristics ensure that the metabolites, including trans-p-coumaric acid and MCPHB, achieve the desired concentration in the bloodstream and target tissues, enhancing their efficacy and safety profiles.

### Medicinal chemistry analysis

The important parameters of medicinal chemistry include PAINS (pan-assay interference metabolites), Brenk (structural alerts), lead-likeness, and synthetic accessibility. These parameters help identify metabolites that may provide false-positive results in assays. PAINS predict the substructure of metabolites that may exhibit biological activity, and all the metabolites show 0 alerts for PAINS. Brenk structural alerts were identified with none of the metabolites except trans-p-coumaric acid, methylcoumarate, and scoparone showing 1 alert. Lead-likeness, which predicts the probability of a metabolite being a lead in drug discovery and increases the chances of success in clinical trials, was observed only in MCPHB among all the metabolites. Synthetic accessibility scores, which range from 1 to 10 and indicate the complexity of the synthetic process, showed that all our top metabolites have simple synthetic processes with scores ranging from 1 to 5 (**Supplementary Table S7**).

To summarize, our medicinal chemistry of the evaluated metabolites generally lacks problematic structural alerts and exhibits favorable properties for drug development. Notably, one metabolite (MCPHB) shows high potential as a lead candidate, and all metabolites possess simple synthetic routes, suggesting they are promising candidates for further research and development.

### Pharmacokinetic analysis

The pharmacokinetic analysis focuses on evaluating the absorption, distribution, metabolism, and excretion (ADME) profiles of the metabolites under study. This section provides a detailed assessment of the behavior of the metabolites within biological systems to determine their potential efficacy, safety, and suitability for further development as therapeutic agents (Shah et al., 2023). It includes Caco-2 permeability (Caco-2), P-glycoprotein substrate (P-gps), P-glycoprotein inhibitor (P-gpi), CYP substrates and inhibitors (CYP1A2, CYP2C9, CYP2D6, CYP2C19, and CYP3A4), human intestinal absorption (HIA), CYP inhibitory promiscuity (CYPPRO), carcinogenicity (CARC), human ether-a-go-go-related gene inhibition (hERG), and the organic cation transporter protein 2 inhibitor (OCT2i) (**Supplementary Table S8**).

All the top metabolites exhibit high values for human intestinal absorption (HIA), indicating their strong potential for efficient absorption from the gastrointestinal tract upon oral administration (Nainwal et al., 2020). Moreover, all the top 10 metabolites exhibit values within the range of −5 to −7.7 for skin permeability (LogKp), indicating their potential to effectively permeate through the outer layer of the skin. This suggests that these metabolites are well-suited for transdermal delivery applications, offering an alternative administration route for treating localized fungal infections (Chen et al., 2018).

All the top 10 metabolites exhibit a high probability of being P-glycoprotein (P-gp) substrates, and most are also predicted to be P-gp inhibitors, with the exceptions of isoliensinine, neferine, and liensinine (**Supplementary Table S8**). This suggests that these metabolites are likely to interact with P-gp, which may influence drug transport and metabolism (Broccatelli et al., 2011; Thapa et al., 2023).

All the metabolites, except 2,6-Dihydroxy-4-methoxyacetophenone and isoliensinine, exhibited plasma protein binding values below 90%, indicating an optimal therapeutic index. Additionally, all metabolites, except eudesmic acid, demonstrated the ability to penetrate the blood-brain barrier (BBB), suggesting potential access to the central nervous system (CNS) (**Supplementary Table S8**). While *C. auris* is not primarily known as a CNS pathogen, it can cause severe systemic infections, including CNS involvement, particularly in immunocompromised individuals. The ability of these antifungal metabolites to cross the BBB is therefore significant for treating rare but serious CNS infections caused by *C. auris*.

Cytochrome P450 enzymes, particularly various isoforms, are responsible for approximately 90% of oxidative metabolic reactions (Guan et al., 2019). Among the studied metabolites, 4-hydroxybenzoate, trans-p-coumaric acid, isoliensinine, neferine, eudesmic acid, and linsinine showed no inhibitory potential against cytochrome P450 enzymes. However, several metabolites acted as inhibitors for specific isozymes: eudesmic acid, 2,6-dihydroxy-4-methoxyacetophenone, scoparone, and MCPHB were identified as inhibitors of CYP1A2, with MCPHB also inhibiting CYP2C19 and CYP2C9. Notably, none of the top metabolites were predicted to inhibit CYP2D6 or CYP3A4.Regarding substrate activity, CYP2C9 was identified as a substrate for methylcoumarate, 2,6-dihydroxy-4-methoxyacetophenone, trans-p-coumaric acid, isoliensinine, neferine, linsinine, and scoparone. Additionally, CYP1A2 served as a substrate for 2,6-dihydroxy-4-methoxyacetophenone and eudesmic acid. The metabolites isoliensinine, neferine, linsinine, and scoparone also acted as substrates for multiple cytochromes, including CYP1A2, CYP2C9, CYP2D6, and CYP3A4. The results suggest that the studied metabolites have favorable metabolic profiles, with minimal cytochrome P450 inhibition, reducing the risk of drug-drug interactions during antifungal therapy targeting *C. auris*.

Toxicity is one of the important properties of a metabolite to be drug candidates. Most of the drug failures are due to the high toxicity of the drugs because they damage different organs of the human body. The important parameters of toxicity are studied as human Ether-à-Go-Related Gene (hERG) blockers, human hepatotoxicity (H-HT), AMES toxicity, skin sensitization, rat oral acute toxicity, drug-induced liver injury (DILI), carcinogenicity, eye corrosion, eye irritation, respiratory toxicity, and maximum recommended daily dose (FDAMDD) (Kalyaanamoorthy and Barakat, 2018). Metabolites such as 4-hydroxybenzoate, methylcoumarate, 2,6-dihydroxy-4-methoxyacetophenone, trans-p-coumaric acid, scoparone, and MCPHB have demonstrated minimal activity as hERG blockers and align with acceptable Maximum Recommended Daily Dose (FDAMDD) levels. These results suggest that these metabolites are likely to have a lower risk of cardiotoxicity and are safer for further development as antifungal agents targeting *C. aureus*.

Based on the pharmacokinetic and safety analysis, 4-hydroxybenzoate, methylcoumarate, 2,6-dihydroxy-4-methoxyacetophenone, trans-p-coumaric acid, scoparone, and MCPHB emerge as the most promising candidates. These metabolites exhibit favorable absorption and distribution profiles, minimal cytochrome P450 inhibition, and lower hERG blocker activity, reducing the risk of cardiotoxicity. Their alignment with acceptable Maximum Recommended Daily Dose (FDAMDD) levels further supports their potential for safe and effective use in antifungal therapy targeting *C. auris*.

### PBPK simulation analysis

The PBPK simulations generated concentration-time profiles for the unbound fraction of the compounds, namely coumaric acid, MCPHB, and VNI, in the plasma over 24 hours. These profiles represented their potential efficacy and duration of action in treating *C. auris* infections.

**Figure 12:**
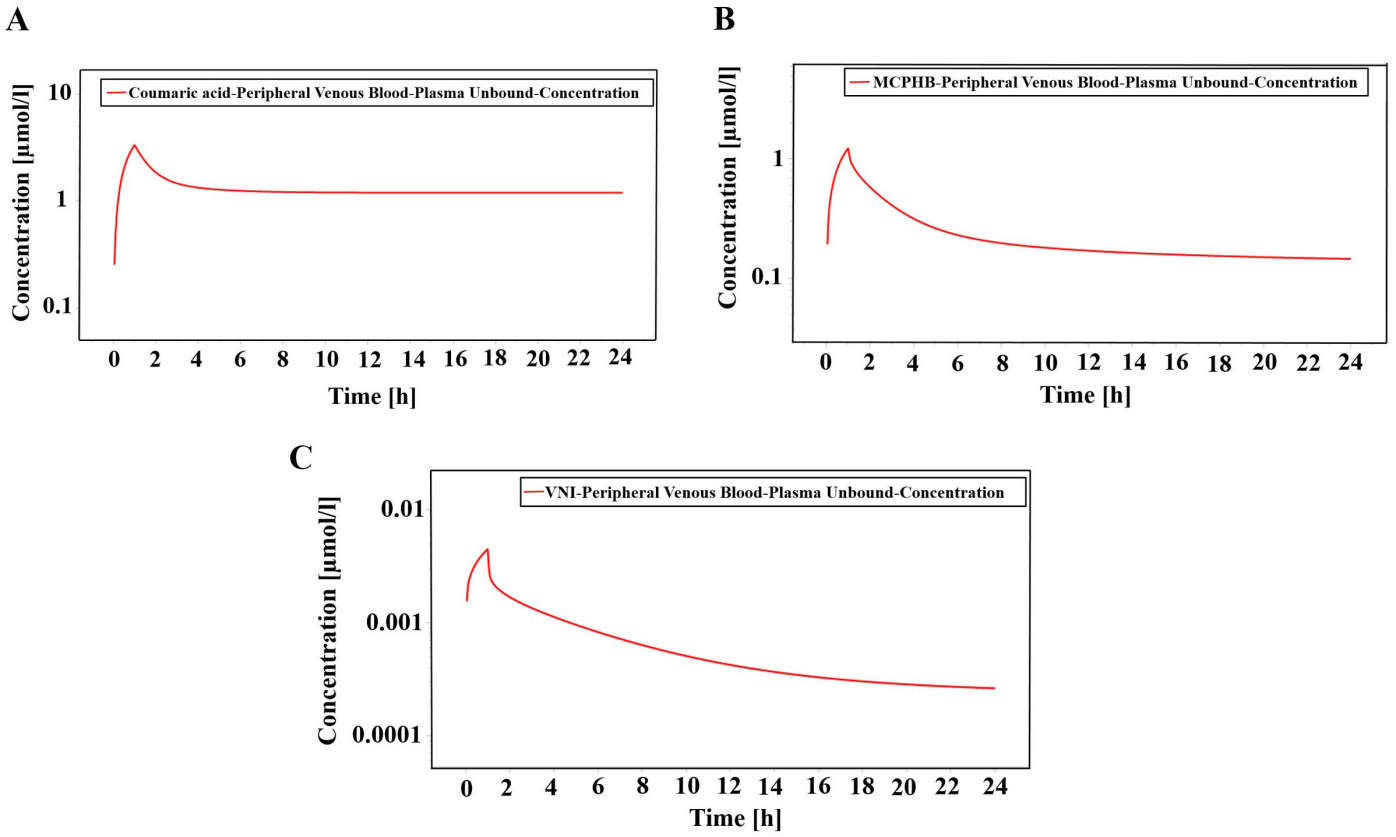
Concentration time profile of metabolites shown in **(A)** coumaric acid, **(B)** (R)-N-(1’-methoxycarbonyl-2’-phenylethyl)-4-hydroxybenzamide (MCPHB), and **(C)** VNI. The red solid line represents varying concentrations of these metabolites in the peripheral venous blood plasma over 24 hours.

In this, the plasma concentration of coumaric acid peaked rapidly at approximately 4 μmol/L within the first hour after post-administration (**Figure 12A**). The concentration then declined to approximately 2 μmol/L within an hour, then followed a gradual pattern and maintained sustained levels over 24 hours. At 24 hours, the concentration remained above 1.2 μmol/L due to a sustained level that suggests a potential for once-daily dosing. Another metabolite, MCPHB, showed a moderate profile compared to coumaric acid with a lower peak concentration of about 1.1 μmol/L (**Figure 12B**). Its elimination shows faster than coumaric acid, due to rapid concentrations dropping to approximately 0.2 μmol/L by 24 hours. This shows more frequent dosing due to rapid elimination compared to coumaric acid. The plasma concentration profile of VNI shows an initial rapid increase peaking at approximately 0.004 μmol/L within the first hour after administration (**Figure 12C**). Following the peak, the concentration gradually declined, reaching approximately 0.0002 μmol/L by 24 hours suggesting that it may have a moderate duration of action. However, its comparatively low plasma concentration indicates the need for dose optimization or alternative formulations to enhance efficacy.

Comparatively, the peak concentrations can be indicated as coumaric acid > MCPHB > VNI. Coumaric acid shows more sustained and higher plasma concentrations that suggest that it may have the most promising pharmacokinetic profile for targeting *C. auris* infections. It shows superior plasma retention due to its moderate lipophilicity and high solubility. MCPHB shows a balanced profile with initial concentrations and moderate sustained levels that are potentially suitable for twice-daily dosing. Isolinsinine may require significant dose adjustments or alternative formulation strategies due to its extremely low plasma concentrations.

All three compounds demonstrate varying capabilities to maintain plasma concentrations, while coumaric acid and MCPHB show the most promising candidates. These results show that our top compounds performed better than the standard compound. Further experimental studies are required to confirm the antifungal activity and safety profiles of these metabolites to assess their potential as treatments for C. auris infections fully.

### Target prediction analysis

An important analysis was performed to identify the human possible targets and evaluate the comprehensive pharmacodynamic profile of our top 10 metabolites for treating *C. auris* disease subjected through the Swiss target prediction online tool. Through this analysis, we can predict both beneficial and harmful interactions within the body that help in evaluating the drug’s efficacy and safety, as they can affect both the disease pathology and the drug’s therapeutic potential.

Among our top metabolites, 4-hydroxybenzoate (**Supplementary Figure S7A**) shows a high probability of interaction with carbonic anhydrase receptor protein (**Supplementary Table S9**). Among the top 15 results, it shows maximum interaction with this class of carbonic anhydrase receptor protein (80%), according to the pie chart. This receptor protein is involved in many biological processes such as respiration, pH homeostasis, fungal growth, virulence, and CO_2_ transport. The carbonic anhydrase (CA) is a metalloenzyme that catalyzes the essential physiological reaction. It is efficiently involved in the interconversion of carbon dioxide (CO_2_) and bicarbonate, which is essential for fungal virulence in *C. auris.* By targeting CA, it can be a potential target for developing new antifungal drugs (Akhtar et al., 2023b). Previously, studies also proved that CA is a crucial target for antifungal drug discovery (Schlicker et al., 2009; Supuran and Capasso, 2021). These metabolites, trans-p-coumaric acid (**Supplementary Figure S7B**), eudesmic acid/3,4,5-trimethoxybenzoic acid (**Supplementary Figure S7C**), and scoparone (**Supplementary Figure S7D**), have the maximum probability (73.3%) to interact with the carbonic anhydrase receptor protein, which belongs to the target class of the Lyase family. Trans-p-coumaric acid metabolite also shows interaction with AKR1B1 receptor protein, but this metabolite is not directly linked with our target diseases.

**Figure 13:**
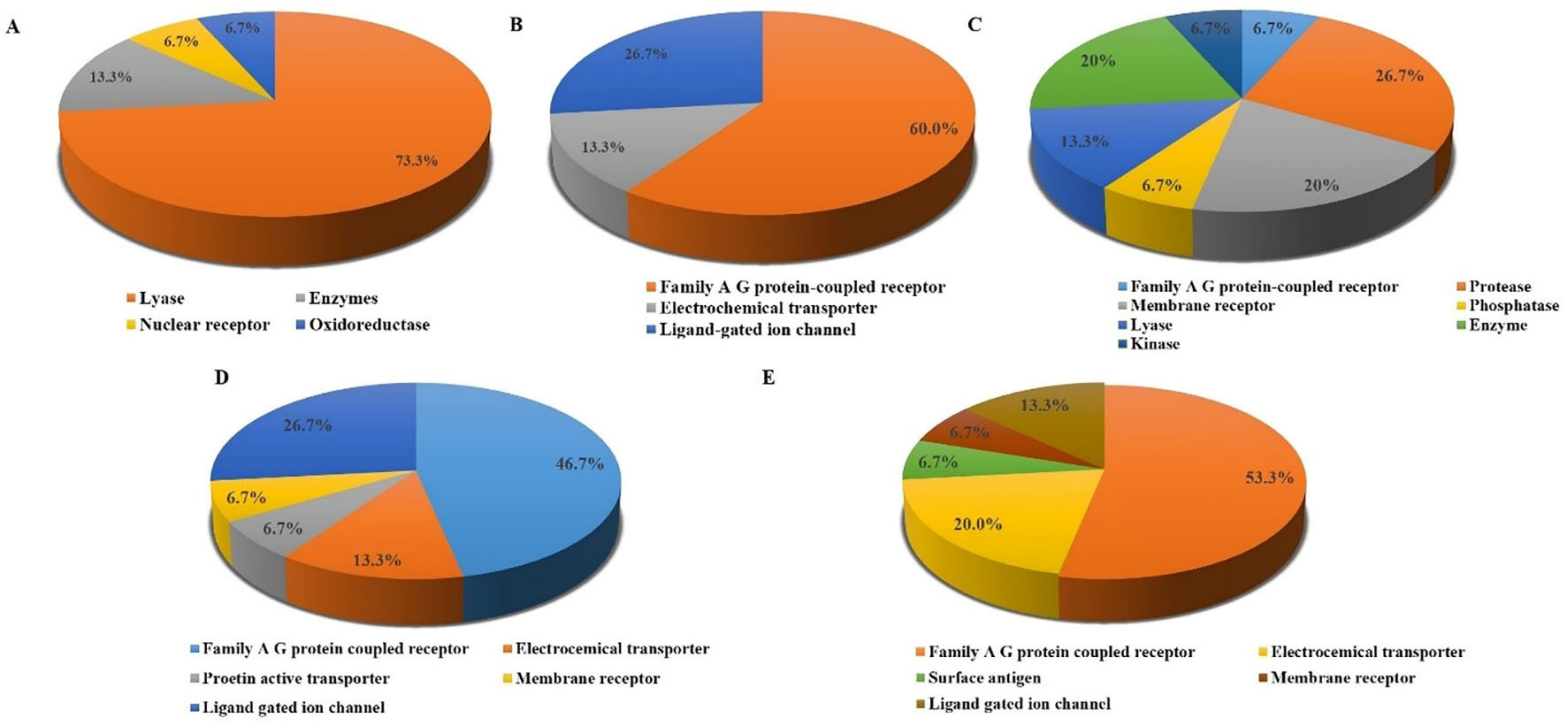
Target prediction of molecules **(A)** 2,6-Dihydroxy-4-methoxyacetophenone, **(B)** Isoliensinine, **(C)** Liensinine, **(D)** Neferine, and **(E)** MCPHB with different receptor proteins shown in the pie chart diagram.

Another top metabolite, methylcoumarate, shows maximum interaction with the carbonic anhydrase receptor (66.7%) according to the pie chart (**Supplementary Figure S7E**). Metabolite 2,6-dihydroxy-4-methoxyacetophenone (**Figure 13A**) also shows maximum interaction with the carbonic anhydrase receptor (40%). It also can interact with other classes of target proteins, including serine/threonine-protein kinase/endoribonuclease IRE1 (13.3%) and cannabinoid receptor protein (13.3%). This IRE1 receptor protein plays a vital role in the fungal pathogenesis mechanism (Akhtar et al., 2023a). Cannabinoid receptor protein, which belongs to Family A G protein-coupled receptor, is not related to our disease directly or indirectly, but this shows the broad-spectrum activity of our metabolite that it can also be used in other diseases or benefits for other processes, but further validation is required to confirm its activity. Our metabolites Isoliensinine (**Figure 13B**), Liensinine (**Figure 13C**), and Neferine (**Figure 13D**) show the maximum probability of interaction with the dopamine receptor protein, which belongs to the target class of a G protein-coupled receptor. This receptor is also not directly linked with our disease, but this shows the interactions with other diseases, including Parkinson’s disease (Deeb et al., 2010). (R)-MCPHB (**Figure 13E**) shows interaction with different classes of receptor proteins, including C-C chemokine receptor type 3, cystinyl aminopeptidase, and protein-tyrosine phosphatase 1B, but these receptors are not related to our target disease. These top metabolites, methylcoumarate, 2,6-dihydroxy-4-methoxyacetophenone, trans-p-coumaric acid, eudesmic acid, scoparone, and 4-hydroxybenzoate, mostly interact with the CA receptor protein, which is the part of metabolism related to our disease. These metabolites could be beneficial for treating the disease in various ways other than targeting the disease we selected in our study. The results of our top metabolites demonstrated that they have broad-spectrum activity, making them adaptable to treat different infectious diseases.

## Conclusions

This investigation is intended to explore the efficacy of traditional Ayurvedic medicinal plants in treating fungal infections, particularly targeting *C. auris*. Targeting CYP51 is important for maintaining the integrity, fluidity, and permeability of cell membrane structure that contributes a potential pathway for novel antifungal therapeutics. Ayurvedic medicinal plants are well-known for their medicinal activity, including antimicrobial activity, antioxidant, anti-inflammatory, anti-cancer, antifungal, anti-diabetic, antibacterial, and immunomodulatory properties. This study aimed to explore the reported natural products of Ayurvedic medicinal plants against the CYP51 of *C. auris*. A total of 469 reported secondary metabolites of the plant, *S. chirayita* (Roxb.) H.Karst. [Gentianaceae], *A. indicum* (L.) Sweet [Malvaceae], *P. emblica* L. [Phyllanthaceae], *N. nucifera Gaertn*. [Nelumbonaceae], *A. salviifolium* (L.f.) Wangerin [Cornaceae], *R. serpentina* (L.) Benth. ex Kurz [Apocynaceae], *A. racemosus Willd*. [Asparagaceae], and *A. augustum* (L.) L.f. [Malvaceae], were collected and virtually screened against the CYP51, which is an essential component of the fungal cell wall. Through virtual screening, 160 metabolites showed a binding affinity higher than the standard metabolite. Key active site residues, namely HIS461, CYS463, and TRY122, were observed in the interaction patterns of the studied metabolites that predicted the potential to inhibit the ergosterol synthesis, with VNI employed to benchmark the findings. The results from RMSD, RMSF, Rg, and SASA analyses confirmed that all three ligands, including our two metabolites, namely trans-p-coumaric acid and MCPHB, and the co-crystallized ligand VNI, achieved stable binding with the protein throughout the MD simulations. The PCA and cross-correlation analyses revealed distinct patterns of residue motion, where VNI constrained the protein structure, trans-p-coumaric acid promoted higher flexibility, and MCPHB maintained a balance between stability and flexibility. The binding free energy decomposition showed that VNI’s interaction relied heavily on van der Waals forces, trans-p-coumaric acid benefited from solvent interactions, and MCPHB exhibited a balanced contribution from van der Waals, electrostatic, and solvation energies. The per-residue energy decomposition analysis showed that Val86, Tyr73, and Leu76 were crucial residues of binding pockets across all ligands. These analyses highlight that each ligand forms a stable complex in comparison with the standard VNI, maintaining the protein’s structural integrity and making them suitable candidates for further biological investigation. The highlighted pharmacophoric features indicated the significant structural features of the studied metabolites that were required for stable binding with key active site residues. Further analysis indicates that our top 10 metabolites followed the maximum rules of drug-likeness. Pharmacokinetic, toxicity, and target prediction analyses have demonstrated that the top metabolites exhibit low toxicity and do not affect human proteins. Furthermore, our top metabolites showed that they have broad-spectrum activity, making them adaptable to treat different infectious diseases. Natural metabolites explored in this study exhibit significant potential for experimental validation in the search of innovative therapeutic approaches to combat fungal infections.

## Data Availability

The original contributions presented in the study are included in the article/**Supplementary Material**. Further inquiries can be directed to the corresponding authors.

## Supplementary material

Supplementary Figure 1 Superimposing the docked complex (in red) onto the co-crystallized complex (in gold) within the active site using PyMOL (RMSD = 0.980 A^0^).

Supplementary Figure 2 Protein-Ligand Interaction analysis of metabolite VNI; 2D schematic interactions with bond distance **(A)**; 3D interactions with amino acids residues **(B)** and 3D conformation **(C)** in the binding pocket of the target protein.

Supplementary Figure 3 Protein-Ligand Interaction analysis of metabolite Isoliensinine; 2D schematic interactions with bond distance **(A)**; 3D interactions with amino acids residues **(B)** and 3D conformation **(C)** in the binding pocket of the target protein.

Supplementary Figure 4 Protein-Ligand Interaction analysis of metabolite Liensinine; 2D schematic interactions with bond distance **(A)**; 3D interactions with amino acids residues **(B)**; and 3D conformation **(C)** in the binding pocket of the target protein.

Supplementary Figure 5 Protein-Ligand Interaction analysis of metabolite Neferine; 2D schematic interactions with bond distance **(A)**; 3D interactions with amino acids residues **(B)**; and 3D conformation **(C)** in the binding pocket of the target protein.

Supplementary Figure 6 Essential pharmacophoric features of the co-crystallized ligand and the standard compound, VNI, for the inhibition of target protein.

Supplementary Figure 7 Target prediction of molecules **(A)** 4-hydroxybenzoate, **(B)** trans-p-coumaric, **(C)** eudesmic acid/3,4,5-Trimethoxybenzoic acid, **(D)** scoparone, and **(E)** methylcoumarate with different receptor proteins shown in a pie chart diagram.
