# Corrigendum: Computational analysis of Ayurvedic metabolites for potential treatment of drug-resistant *Candida auris*

**DOI:** 10.3389/fcimb.2025.1595290

**Published:** 2025-05-08

**Authors:** Mohibullah Shah, Mahnoor Zia, Iqra Ahmad, Muhammad Umer Khan, Hasan Ejaz, Maqsood Alam, Shahid Aziz, Umar Nishan, Hanna Dib, Riaz Ullah, Suvash Chandra Ojha

**Affiliations:** ^1^ Department of Biochemistry, Bahauddin Zakariya University, Multan, Pakistan; ^2^ Institute of Molecular Biology and Biotechnology, The University of Lahore, Lahore, Pakistan; ^3^ Department of Clinical Laboratory Sciences, College of Applied Medical Sciences, Jouf University, Sakaka, Saudi Arabia; ^4^ Department of Biochemistry and Molecular Biology, Federal University of Ceara, Fortaleza, Brazil; ^5^ Department of Chemistry, Kohat University of Science and Technology, Kohat, Pakistan; ^6^ College of Engineering and Technology, American University of the Middle East, Egaila 54200, Kuwait; ^7^ Department of Pharmacognosy, College of Pharmacy, King Saud University, Riyadh, Saudi Arabia; ^8^ Department of Infectious Diseases, The Affiliated Hospital of Southwest Medical University, Luzhou, China

**Keywords:** fungal infections, *Candida auris*, computational chemistry, plants, molecular docking

In the published article, there was an error in **Figure 8** as published. [A part of the figure was not labeled]. The corrected **Figure 8** and its caption appear below.

**Figure 8:**
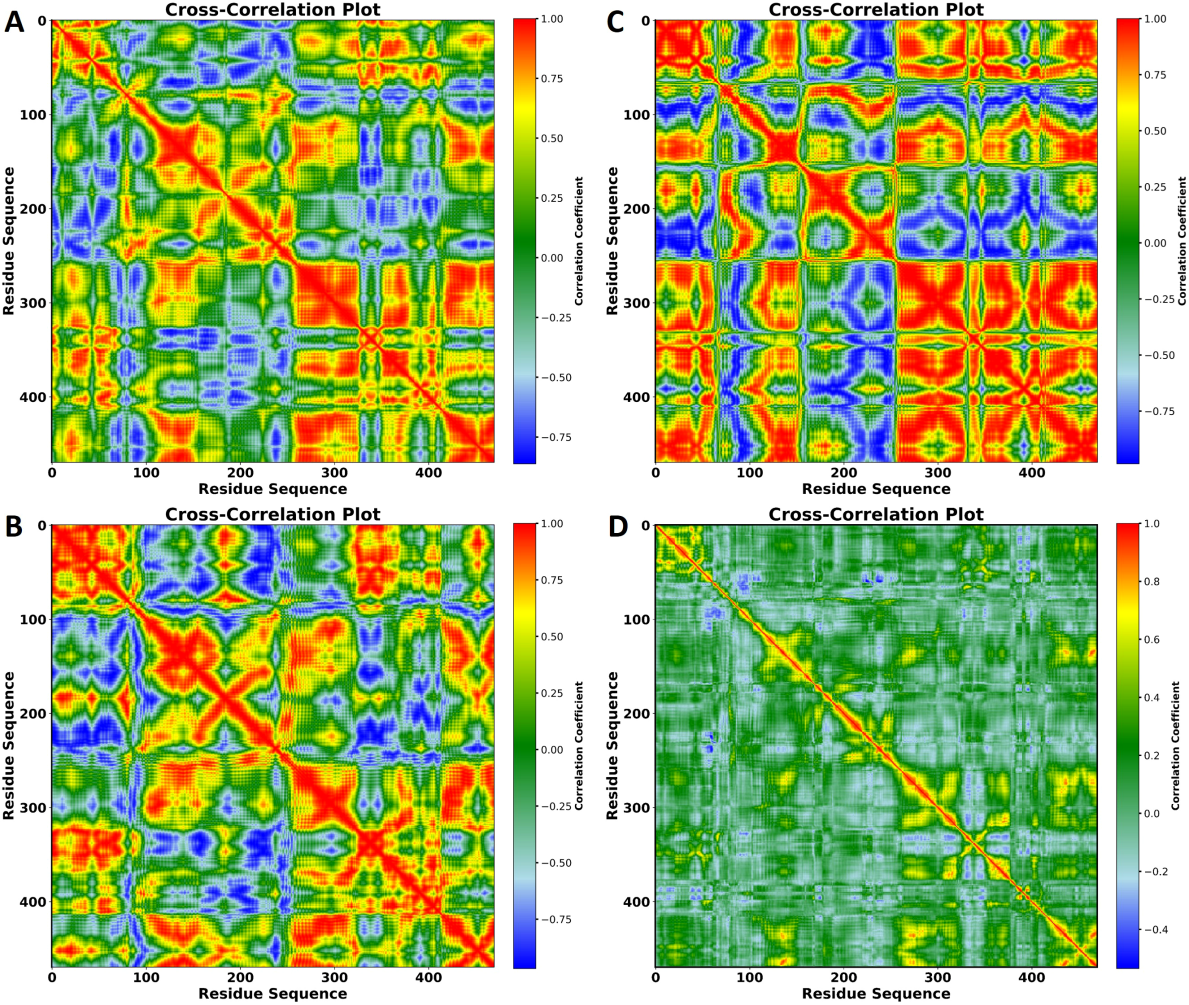
Cross-correlation plots of residue motions in the protein in complexes with **(A)** VNI, **(B)** trans-p-coumaric acid, **(C)** MCPHB, and **(D)** Cross-correlation plot of residue motions in the apoprotein. The x- and y-axes represent the residue sequence, and the color scale on the right indicates the correlation coefficients, ranging from -1 (anti-correlated, blue) to +1 (positively correlated, red). These plots show the degree of correlated and anti-correlated motions between residues throughout the 100 ns MD simulation.

The authors apologize for this error and state that this does not change the scientific conclusions of the article in any way. The original article has been updated.

