# Supplementary material for: Computational analysis of Ayurvedic metabolites for potential treatment of drug-resistant Candida auris
Source: Front Cell Infect Microbiol. 2025 Mar 13;15:1537872. doi: 10.3389/fcimb.2025.1537872 (PMC11979702; doi:10.3389/fcimb.2025.1537872)
Supplement: Supplementary file 1 [file Table1.docx]

**Table S1.** Library of secondary metabolites of Ayurvedic medicinal plants.

| **Sr. No.** | **COMPOUNDS** | **Compound CID** | **Canonical SMILES** | **STRUCTURE** | **Docking Score** | **References** |  |
| --- | --- | --- | --- | --- | --- | --- | --- |
|  | Gammacer-16-en-3-β-ol |  |  |  | Not Interacted | (Chakravarty, Mukhopadhyay, et al., 1991a) |  |
|  | Swerta-7,9(11)-dien-3-β-ol |  |  |  | Not Interacted | (Dey et al., 2020) |  |
|  | Pichierenol |  |  |  | Not Interacted | (Tabassum et al., 2012) |  |
|  | Olean-12-ene-18αH-3-one-9α-ol |  |  |  | Not Interacted | (Bajaj et al., 2017) |  |
|  | mallonin, putranjivain A | 153274338 | C1C(C(C2(O1)C3C45CC(O2)(C6(C(C4C7=C(O6)C(=C(C=C7C(=O)OC8C9C(C(COC(=O)C1=CC(=C(C(=C1C1=C(C(=C(C=C1C(=O)O9)O)O)O)O)O)O)OC8OC(=O)C1=CC(=C(C(=C1)O)O)O)OC5=O)O)O)(O3)O)O)O)O)O |  | Not Interacted | (H. Kim et al., 1995) |  |
|  | camphor | 2537 | CC1(C2CCC1(C(=O)C2)C)C |  | Not Interacted | (Dong et al., 2007) |  |
|  | borneol | 64685 | CC1(C2CCC1(C(C2)O)C)C |  | Not Interacted | (Dong et al., 2007) |  |
|  | methyl-4-hydroxybenzoate | 49775374 | C1=COC(C2C1C(C=C2COC(=O)C3=CC=C(C=C3)O)O)O.C1=COC(C2C1C(C=C2COC(=O)C3=CC=C(C=C3)O)O)OC4C(C(C(C(O4)CO)O)O)O |  | Not Interacted | (Costa et al., 1992) |  |
|  | 3S,5R-Dihydroxy-6S,7-megastigmadien-9-one |  |  |  | Not Interacted | (Ahn et al., 2013b) |  |
|  | ( 6R,6aR)roemerine-N(β)-oxide |  |  |  | Not Interacted | (Ahn et al., 2013b) |  |
|  | Dihydrophaseic acid 3’-O-beta-D-glucopyranoside | 11988281 | CC(=CC(=O)O)C=CC1(C2(CC(CC1(OC2)C)OC3C(C(C(C(O3)CO)O)O)O)C)O |  | Not Interacted | (Youn et al., 2011) |  |
|  | Rauwolfine | 6100671 | CCC1C2CC3C4C5(CC(C2C5O)N3C1O)C6=CC=CC=C6N4C |  | Not Interacted | (Itoh et al., 2005) |  |
|  | Ajmaline | 6100671 | CCC1C2CC3C4C5(CC(C2C5O)N3C1O)C6=CC=CC=C6N4C |  | Not Interacted | (Srivastava et al., 2006) |  |
|  | Methyl (3β,13α,14β,20α)-3-hydroxy-13-methyl-26-norolean-8-en-  29-oate |  |  |  | Not Interacted | (Swati, Bhatt, Sendri, …, et al., 2023) |  |
|  | Shatavarin-V |  |  |  | Not Interacted | (P. Hayes et al., 2006) |  |
|  | Abromasterol |  |  |  | Not Interacted | (Dasgupta & Basu, 1970) |  |
|  | Aristophyll-C | 162941351 | CCC1=C(C2=NC1=CC3=C(C4=C(N3)C(=C5C(C(C(=CC6=NC(=C2)C(=C6C)C=C)N5)C)CCC(=O)OCC=C(C)CCCC(C)CCCC(C)CCCC(C)C)C(=O)OC4=O)C)C |  | -18.306 | (CHAN et al., 1999) |  |
|  | Pheophytin-a | 135398712 | CCC1=C(C2=NC1=CC3=C(C4=C(C(C(=C5C(C(C(=CC6=NC(=C2)C(=C6C)C=C)N5)C)CCC(=O)OCC=C(C)CCCC(C)CCCC(C)CCCC(C)C)C4=N3)C(=O)OC)O)C)C |  | -17.702 | (Vernon, 1960) |  |
|  | alamandines | 44192273 | CCC(C)C(C(=O)NC(CC1=CN=CN1)C(=O)N2CCCC2C(=O)O)NC(=O)C(CC3=CC=C(C=C3)O)NC(=O)C(C(C)C)NC(=O)C(CCCN=C(N)N)NC(=O)C(C)N |  | -17.167 | (Lautner et al., 2013) |  |
|  | 1-Hentetracontanol | 38627 | CCCCCCCCCCCCCCCCCCCCCCCCCCCCCCCCCCCCCCCCCO |  | -16.561 | (Suja & Sivakala, 2021) |  |
|  | racemoside B | 102253063 | CC1CCC2(C(C3C(O2)CC4C3(CCC5C4CCC6C5(CCC(C6)OC7C(C(C(C(O7)COC8C(C(C(C(O8)COC9C(C(C(C(O9)C)O)O)O)O)O)O)O)O)O)C)C)C)OC1 |  | -15.448 | (Mandal et al., 2006a) |  |
|  | Nelumboroside B |  |  |  | -14.853 | (Sook et al., 2006) |  |
|  | 4-hydroxybenzoate | 54675830 | C1=CC(=CC=C1C(=O)O)[O-] |  | -14.726 | (P. C. Kuo et al., 2008a) |  |
|  | methylcoumarate | 5319562 | COC(=O)C=CC1=CC=C(C=C1)O |  | -14.377 | (Wu et al., 2004) |  |
|  | 2,6-Dihydroxy-4-methoxyacetophenone | 24135 | CC(=O)C1=C(C=C(C=C1O)OC)O |  | -14.145 | (Kokubun et al., 1994) |  |
|  | trans-p-coumaric acid | 637542 | C1=CC(=CC=C1C=CC(=O)O)O |  | -14.069 | (P. C. Kuo et al., 2008a) |  |
|  | Isoliensinine | 5274591 | CN1CCC2=CC(=C(C=C2C1CC3=CC=C(C=C3)OC)OC4=C(C=CC(=C4)CC5C6=CC(=C(C=C6CCN5C)OC)O)O)OC |  | -14.048 | (Y. Chen et al., 2007) |  |
|  | Tritriacontane-9,10-diol |  |  |  | -13.986 | (Koch et al., 2006) |  |
|  | Shatavarin-VII | 101847688 | CC1C2C(CC3C2(CCC4C3CCC5C4(CCC(C5)OC6C(C(C(C(O6)CO)OC7C(C(C(C(O7)C)O)O)O)O)OC8C(C(C(C(O8)CO)O)O)O)C)C)OC19CCC(=C)CO9 |  | -13.926 | (Kumeta et al., 2013), (P. Y. Hayes et al., 2008a) |  |
|  | Rescinnamidine | 184180 | COC1C(CC2CN3CCC4=C(C3CC2C1C(=O)OC)NC5=C4C=CC(=C5)OC)OC(=O)CCC6=CC(=C(C(=C6)OC)OC)OC |  | -13.859 | (Siddiqui, Haider, et al., 1987a) |  |
|  | Hentriacontane-12,15-diol | 85780241 | CCCCCCCCCCCCCCCCC(CCC(CCCCCCCCCCC)O)O |  | -13.834 | (Koch et al., 2006) |  |
|  | Neferine | 159654 | CN1CCC2=CC(=C(C=C2C1CC3=CC=C(C=C3)OC)OC4=C(C=CC(=C4)CC5C6=CC(=C(C=C6CCN5C)OC)OC)O)OC |  | -13.749 | (Y. Chen et al., 2007) |  |
|  | Nonacosane-10,13-diol | 85977306 | CCCCCCCCCCCCCCCCC(CCC(CCCCCCCCC)O)O |  | -13.463 | (Koch et al., 2006) |  |
|  | Rescinnamine | 5280954 | COC1C(CC2CN3CCC4=C(C3CC2C1C(=O)OC)NC5=C4C=CC(=C5)OC)OC(=O)C=CC6=CC(=C(C(=C6)OC)OC)OC |  | -13.415 | (Cronheim et al., 1954) |  |
|  | Shatavarin VI | 101847687 | CC1CCC2(C(C3C(O2)CC4C3(CCC5C4CCC6C5(CCC(C6)OC7C(C(C(C(O7)CO)OC8C(C(C(C(O8)C)O)O)O)O)OC9C(C(C(C(O9)CO)O)O)O)C)C)C)OC1 |  | -13.403 | (P. Y. Hayes et al., 2008a) |  |
|  | Eudesmic acid | 8357 | COC1=CC(=CC(=C1OC)OC)C(=O)O |  | -13.255 | (Rajput & Patel, 2012) |  |
|  | Liensinine | 160644 | CN1CCC2=CC(=C(C=C2C1CC3=CC=C(C=C3)O)OC4=C(C=CC(=C4)CC5C6=CC(=C(C=C6CCN5C)OC)OC)O)OC |  | -13.243 | (Y. Chen et al., 2007) |  |
|  | scoparone | 8417 | COC1=C(C=C2C(=C1)C=CC(=O)O2)OC |  | -13.205 | (Razdan et al., 1987) |  |
|  | Racemoside c | 102253064 | CC1CCC2(C(C3C(O2)CC4C3(CCC5C4CCC6C5(CCC(C6)OC7C(C(C(C(O7)COC8C(C(C(C(O8)C)O)O)O)OC9C(C(C(C(O9)C)O)O)O)O)O)C)C)C)OC1 |  | -13.203 | (Mandal et al., 2006a) |  |
|  | Glycine, N-[(3.alpha.,5.beta.,12.alpha.) | 22214182 | CC(CCC(=O)NCC(=O)OC)C1CCC2C1(C(CC3C2CCC4C3(CCC(C4)O[Si](C)(C)C)C)O[Si](C)(C)C)C |  | -13.160 | (Suja & Sivakala, 2021) |  |
|  | Shatavarin - X | 101847691 | CC1CCC2(C(C3C(O2)CC4C3(CCC5C4CCC6C5(CCC(C6)OC7C(C(C(C(O7)CO)OC8C(C(C(C(O8)COC(=O)C)O)O)O)O)OC9C(C(C(C(O9)CO)O)O)O)C)C)C)OC1 |  | -13.092 | (P. Y. Hayes et al., 2006a) |  |
|  | Shatavarin-IV / Asparanin B | 441896 | CC1CCC2(C(C3C(O2)CC4C3(CCC5C4CCC6C5(CCC(C6)OC7C(C(C(C(O7)CO)OC8C(C(C(C(O8)C)O)O)O)O)OC9C(C(C(C(O9)CO)O)O)O)C)C)C)OC1 |  | -13.058 | (P. Y. Hayes et al., 2006a) |  |
|  | Reserpine | 5770 | COC1C(CC2CN3CCC4=C(C3CC2C1C(=O)OC)NC5=C4C=CC(=C5)OC)OC(=O)C6=CC(=C(C(=C6)OC)OC)OC |  | -13.032 | (Klohs et al., 1954) |  |
|  | Shatavarin-I | 101406647 | CC1C2C(CC3C2(CCC4C3CCC5C4(CCC(C5)OC6C(C(C(C(O6)CO)OC7C(C(C(C(O7)C)O)O)O)O)OC8C(C(C(C(O8)CO)O)O)O)C)C)OC1(CCC(C)COC9C(C(C(C(O9)CO)O)O)O)O |  | -12.965 | (P. Y. Hayes et al., 2006b) |  |
|  | (R)-N-(1’-methoxycarbonyl-2’-phenylethyl)-4-hydroxybenzamide | 73941365 | COC(=O)C(CC1=CC=CC=C1)NC(=O)C2=CC=C(C=C2)O |  | -12.939 | (P. C. Kuo et al., 2008b) |  |
|  | asparanin-A | 21575007 | CC1CCC2(C(C3C(O2)CC4C3(CCC5C4CCC6C5(CCC(C6)OC7C(C(C(C(O7)CO)O)O)OC8C(C(C(C(O8)CO)O)O)O)C)C)C)OC1 |  | -12.878 | (Sharma et al., 1982) |  |
|  | Octacosanol | 68406 | CCCCCCCCCCCCCCCCCCCCCCCCCCCCO |  | -12.875 | (Pollard et al., 1933) |  |
|  | Abromine | 247 | C[N+](C)(C)CC(=O)[O-] |  | -12.852 | (Hoare & Hiscock, 1974) |  |
|  | Nonacosane-4,10-diol | 85977331 | CCCCCCCCCCCCCCCCCCCC(CCCCCC(CCC)O)O |  | -12.814 | (Jetter & Riederer, 1995) |  |
|  | Triacontane-7-ol | 101428905 | CCCCCCCCCCCCCCCCCCCCCCCC(CCCCCC)O |  | -12.792 | (Moradinia & Teja, 1986) |  |
|  | 4-hydroxy-3-methoxy-trans-cinnamic acid methyl ester / Methyl isoferulate | 6439893 | COC1=C(C=C(C=C1)C=CC(=O)OC)O |  | -12.638 | (Shimazono, 1959) |  |
|  | Nonacosane-5,10-diol | 13797568 | CCCCCCCCCCCCCCCCCCCC(CCCCC(CCCC)O)O |  | -12.569 | (Hunt & Baker, 1979) |  |
|  | Geraniin | 3001497 | C1C2C3C(C(C(O2)OC(=O)C4=CC(=C(C(=C4)O)O)O)OC(=O)C5=CC(=C(C6=C5C7C(=CC(=O)C(C7(O)O)(O6)O)C(=O)O3)O)O)OC(=O)C8=CC(=C(C(=C8C9=C(C(=C(C=C9C(=O)O1)O)O)O)O)O)O |  | -12.280 | (Y.-C. Yang et al., 2010) |  |
|  | Dauricine | 73400 | CN1CCC2=CC(=C(C=C2C1CC3=CC=C(C=C3)OC4=C(C=CC(=C4)CC5C6=CC(=C(C=C6CCN5C)OC)OC)O)OC)OC |  | -12.276 | (Shujuan et al., 2000) |  |
|  | Isorhamnetin 3-O-α-L-rhamnopyranosyl  (1→6)-β-D-glucopyranoside |  |  |  | -12.252 | (Yuca et al., 2022) |  |
|  | 3,7-dihydroxy chromen-2-one | 6918810 | C1=CC2=C(C=C1O)OC(=O)C(=C2)O |  | -12.252 | (Battersby et al., 1968) |  |
|  | Isorhamnetin 3-O-rutinoside | 5481663 | CC1C(C(C(C(O1)OCC2C(C(C(C(O2)OC3=C(OC4=CC(=CC(=C4C3=O)O)O)C5=CC(=C(C=C5)O)OC)O)O)O)O)O)O |  | -12.248 | (Cubukcu & Yazgan, 1974) |  |
|  | Aceclofenac | 71771 | C1=CC=C(C(=C1)CC(=O)OCC(=O)O)NC2=C(C=CC=C2Cl)Cl |  | -12.240 | (H. S. Lee et al., 2000) |  |
|  | Kaempferol 3-O-alpha-L-rhamnopyranosyl-(1-2)-beta-D glucuronopyranoside |  |  |  | -12.233 | (da Silva Oliveira et al., 2022) |  |
|  | Kaempferol 3-O-alpha-L-rhamnopyranosyl-(1-2)-beta-D glucopyranoside |  |  |  | -12.233 | (da Silva Oliveira et al., 2022) |  |
|  | alangamide |  |  |  | -12.188 | (J. Singh & Trivedi, 1978) |  |
|  | Shatavarin-IX | 101847690 | CC1CCC2(C(C3C(O2)CC4C3(CCC5C4CCC6C5(CCC(C6)OC7C(C(C(C(O7)CO)OC8C(C(C(C(O8)CO)O)O)O)O)OC9C(C(C(C(O9)CO)O)O)O)C)C)C)OC1 |  | -12.140 | (Onlom et al., 2017) |  |
|  | serpentinine | 5351576 | CC=C1CN2CCC3=C(C2CC1C(CC4=C[N+]5=C(CC6C(C5)C(OC=C6C(=O)OC)C)C7=C4C8=CC=CC=C8N7)C(=O)OC)NC9=CC=CC=C39 |  | -12.043 | (Mengoni et al., 2001) |  |
|  | cholest-5,24-dien-3beta-yl beta-D-glucopyranoside | 70699276 | CC(CCC=C(C)C)C1CCC2C1(CCC3C2CC=C4C3(CCC(C4)OC5C(C(C(C(O5)CO)O)O)O)C)C |  | -12.036 | (Zacher et al., 2018) |  |
|  | Ferulic acid | 445858 | COC1=C(C=CC(=C1)C=CC(=O)O)O |  | -12.035 | (Garcia-Conesa et al., 1997) |  |
|  | β-Sitosterol-D-glucoside | 12309060 | CCC(CCC(C)C1CCC2C1(CCC3C2CC=C4C3(CCC(C4)OC5C(C(C(C(O5)CO)O)O)O)C)C)C(C)C |  | -11.995 | (Hossain et al., 2019) |  |
|  | aurantiamide acetate | 124319 | CC(=O)OCC(CC1=CC=CC=C1)NC(=O)C(CC2=CC=CC=C2)NC(=O)C3=CC=CC=C3 |  | -11.970 | (Olutola Dosumu et al., 2014) |  |
|  | Ginnol | 16057860 | CCCCCCCCCCCCCCCCCCCC(CCCCCCCCC)O |  | -11.910 | (Berkenbusch & Brückner, 1998) |  |
|  | Vincoside | 101289755 | COC(=O)C1=COC(C(C1CC2C3=C(CCN2)C4=CC=CC=C4N3)C=C)OC5C(C(C(C(O5)CO)O)O)O |  | -11.835 | (Battersby et al., 1968) |  |
|  | Kaempferol 3-O-robinobioside | 15944778 | CC1C(C(C(C(O1)OCC2C(C(C(C(O2)OC3=C(OC4=CC(=CC(=C4C3=O)O)O)C5=CC=C(C=C5)O)O)O)O)O)O)O |  | -11.799 | (Yasukawa & Takido, 1987) |  |
|  | deserpidine | 8550 | COC1C(CC2CN3CCC4=C(C3CC2C1C(=O)OC)NC5=CC=CC=C45)OC(=O)C6=CC(=C(C(=C6)OC)OC)OC |  | -11.791 | (MacPhillamy et al., 1955) |  |
|  | Shatavarin-VIII | 101847689 | CC1CCC2(C(C3C(O2)CC4C3(CCC5C4CCC6C5(CCC(C6)OC7C(C(C(C(O7)COC8C(C(C(C(O8)CO)O)O)O)OC9C(C(C(CO9)O)O)O)O)OC2C(C(C(C(O2)CO)O)O)O)C)C)C)OC1 |  | -11.787 | (P. Y. Hayes et al., 2008b) |  |
|  | Nonacosan-10-ol | 25240035 | CCCCCCCCCCCCCCCCCCCC(CCCCCCCCC)O |  | -11.713 | (Jetter & Riederer, 1994) |  |
|  | Paracetamol | 1983 | CC(=O)NC1=CC=C(C=C1)O |  | -11.683 | (Clissold, 1986) |  |
|  | Luteolin-7-O-β-glucopyranoside | 5280637 | C1=CC(=C(C=C1C2=CC(=O)C3=C(C=C(C=C3O2)OC4C(C(C(C(O4)CO)O)O)O)O)O)O |  | -11.664 | (Kisiel, 2014) |  |
|  | psychotrine | 65380 | CCC1CN2CCC3=CC(=C(C=C3C2CC1CC4=NCCC5=CC(=C(C=C54)OC)O)OC)OC |  | -11.659 | (Wiegrebe et al., 1984) |  |
|  | racemoside A | 102253062 | CC1CCC2(C(C3C(O2)CC4C3(CCC5C4CCC6C5(CCC(C6)OC7C(C(C(C(O7)COC8C(C(C(C(O8)CO)O)O)O)OC9C(C(C(C(O9)COC2C(C(C(C(O2)C)O)O)O)O)O)O)O)O)C)C)C)OC1 |  | -11.630 | (Mandal et al., 2006b) |  |
|  | dimethylphychotrine |  |  |  | -11.612 | (Tan et al., 1991) |  |
|  | Kaempferol 3-O-alpha-L-rhamnopyranosyl-(1-6)-beta-D glucopyranoside |  |  |  | -11.577 | (Astiti et al., 2021) |  |
|  | punicafolin | 5320800 | C1C2C(C(C(C(O2)OC(=O)C3=CC(=C(C(=C3)O)O)O)OC(=O)C4=CC(=C(C(=C4)O)O)O)OC(=O)C5=CC(=C(C(=C5C6=C(C(=C(C=C6C(=O)O1)O)O)O)O)O)O)OC(=O)C7=CC(=C(C(=C7)O)O)O |  | -11.438 | (Tanaka et al., 1985) |  |
|  | quercetin-3-O-b-D-xylopyranosyl-(1-2)-b-D glucopyranosyl  glycoside | 10167806 |  |  | -11.430 | (Jung et al., 2015) |  |
|  | Tercatain | 14411426 | C1C2C(C(C(C(O2)OC(=O)C3=CC(=C(C(=C3)O)O)O)O)OC(=O)C4=CC(=C(C(=C4C5=C(C(=C(C=C5C(=O)O1)O)O)O)O)O)O)OC(=O)C6=CC(=C(C(=C6)O)O)O |  | -11.385 | (TANAKA et al., 1986) |  |
|  | Isoschaftoside | 3084995 | C1C(C(C(C(O1)C2=C(C(=C3C(=C2O)C(=O)C=C(O3)C4=CC=C(C=C4)O)C5C(C(C(C(O5)CO)O)O)O)O)O)O)O |  | -11.358 | (Qimin et al., 1991) |  |
|  | cis 12, 13-epoxyoleic (vernolic) acid | 6449780 | CCCCCC1C(O1)CC=CCCCCCCCC(=O)O |  | -11.312 | (Morris & Crouchman, 1969) |  |
|  | Sapogenin A | 23265676 | CC1(C(CCC2(C1C(CC3C2=CCC4(C3(CC(C4C5(CCC(O5)C(C)(C)O)C)O)C)C)O)C)O)C |  | -11.249 | (TAKAGI et al., 1980) |  |
|  | tubulosine | 72341 | CCC1CN2CCC3=CC(=C(C=C3C2CC1CC4C5=C(CCN4)C6=C(N5)C=CC(=C6)O)OC)OC |  | -11.247 | (Bhakuni et al., 1983) |  |
|  | Quercetin 3-O-beta-D-xylopyranosyl-(1-2)-beta-D galactopyranoside | 5274586 | C1C(C(C(C(O1)OC2C(C(C(OC2OC3=C(OC4=CC(=CC(=C4C3=O)O)O)C5=CC(=C(C=C5)O)O)CO)O)O)O)O)O |  | -11.247 | (Sharoyan et al., 2015) |  |
|  | N-methylcephaeline | 10219 | CCC1CN2CCC3=CC(=C(C=C3C2CC1CC4C5=CC(=C(C=C5CCN4)OC)OC)OC)OC |  | -11.246 | (Carr & Pyman, 1914) |  |
|  | Asparacoside | 21575006 | CC1CCC2(C(C3C(O2)CC4C3(CCC5C4CCC6C5(CCC(C6)OC7C(C(C(C(O7)COC8C(C(C(CO8)O)O)O)OC9C(C(C(CO9)O)O)O)O)OC2C(C(C(C(O2)CO)O)O)O)C)C)C)OC1 |  | -11.246 | (H.-J. Zhang et al., 2004) |  |
|  | strictosidine | 161336 | COC(=O)C1=COC(C(C1CC2C3=C(CCN2)C4=CC=CC=C4N3)C=C)OC5C(C(C(C(O5)CO)O)O)O |  | -11.222 | (TREIMER & ZENK, 1979) |  |
|  | alangicine | 442158 | CCC1CN2CCC3=C(C(=C(C=C3C2CC1CC4=NCCC5=CC(=C(C=C54)OC)O)OC)OC)O |  | -11.128 | (FUJII et al., 1983) |  |
|  | Syringaresinol-4''-O-β-D-glucopyranoside |  |  |  | -11.101 | (H.-J. Zhang et al., 2004) |  |
|  | Nelumboroside A |  |  |  | -11.077 | (Hyun et al., 2006) |  |
|  | ajmalimine | 53463147 | CCC1C2CC3C4C5(CC(C2C5OC(=O)C6=CC(=C(C(=C6)OC)OC)OC)N3C1O)C7=CC=CC=C7N4C |  | -11.042 | (Siddiqui, Ahmad, et al., 1987) |  |
|  | Chiratanin |  |  |  | -11.031 | (Mandal (née Sarkar) & Chatterjee, 1987) |  |
|  | Emetine | 10219 | CCC1CN2CCC3=CC(=C(C=C3C2CC1CC4C5=CC(=C(C=C5CCN4)OC)OC)OC)OC |  | -11.030 | (Wiegrebe et al., 1984) |  |
|  | Schidigerasaponin B1 | 10629514 | CC1C2C(CC3C2(C(=O)CC4C3CCC5C4(CCC(C5)OC6C(C(C(C(O6)CO)O)OC7C(C(C(CO7)O)O)O)OC8C(C(C(C(O8)CO)O)O)O)C)C)OC19CCC(=C)CO9 |  | -10.988 | (Miyakoshi et al., 2000) |  |
|  | Heptadecanoic acid, 15-methyl-methyl ester / Methyl 15-methylheptadecanoate | 554152 | CCC(C)CCCCCCCCCCCCCC(=O)OC |  | -10.920 | (Sebedio et al., 1989) |  |
|  | Octadecenoic acid, 2-hydroxy-1-(hydroxymethyl) ethyl ester | 71751311 | CCCCCCCCC=CCCCCCCCC(=O)OC(CO)CO |  | -10.910 | (Sanjeev & Doshi, 2018) |  |
|  | Gamma-sitosterol | 457801 | CCC(CCC(C)C1CCC2C1(CCC3C2CC=C4C3(CCC(C4)O)C)C)C(C)C |  | -10.898 | (Thompson et al., 1963) |  |
|  | 8-O-[β-D-xylopyranosyl-(1→6)-β-D-glucopyranosyl]-1,7-dihydroxyl-3-methoxy xanthone |  |  |  | -10.897 | (Thompson et al., 1963) |  |
|  | Vasicine | 667496 | C1CN2CC3=CC=CC=C3N=C2C1O |  | -10.869 | (Hanford et al., 1934) |  |
|  | Gossypetin-7- o- b-glucoside | 14034216 | COC1=C(C=C(C=C1)C2=C(C(=O)C3=C(O2)C(=C(C=C3O)OC)OC)O)OC |  | -10.822 | (Stich et al., 1997) |  |
|  | 3-Hydroxystigmast-5-en-7-one | 160608 | CCC(CCC(C)C1CCC2C1(CCC3C2C(=O)C=C4C3(CCC(C4)O)C)C)C(C)C |  | -10.817 | (Radulovic & Djordjevic, 2011) |  |
|  | 3',8,8'-Trimethoxy-3-piperidyl-2,2'-binaphthalene-1,1',4,4'-tetrone | 590815 | COC1=CC=CC2=C1C(=O)C(=C(C2=O)N3CCCCC3)C4=C(C(=O)C5=C(C4=O)C(=CC=C5)OC)OC |  | -10.794 | (Sharoyan et al., 2015) |  |
|  | 1',2'-Dehydrotubulosine | 135423968 | CCC1CN2CCC3=CC(=C(C=C3C2CC1CC4=NCCC5=C4NC6=C5C=C(C=C6)O)OC)OC |  | -10.772 | (Itoh et al., 2000) |  |
|  | Luteolin | 5280445 | C1=CC(=C(C=C1C2=CC(=O)C3=C(C=C(C=C3O2)O)O)O)O |  | -10.748 | (X.-W. Wang, 2000) |  |
|  | Glucovanilloyl glucose | 14132344 |  |  | -10.701 | (Gaind & Chopra, 1976) |  |
|  | 2,3-Dihydroxypropyl elaidate | 5364833 | CCCCCCCCC=CCCCCCCCC(=O)OCC(CO)O |  | -10.697 | (Khan & Javaid, 2023) |  |
|  | methyl 4-hydroxyphenylacetate | 518900 | COC(=O)CC1=CC=C(C=C1)O |  | -10.690 | (Mao et al., 2006) |  |
|  | 8-O-[β-D-xylopyranosyl-(1→6)-β-D-glucopyranosyl]-1-hydroxyl-3,7-dimethoxy xanthone |  |  |  | -10.675 | (Thompson et al., 1963) |  |
|  | Corilagin | 73568 | C1C2C(C(C(C(O2)OC(=O)C3=CC(=C(C(=C3)O)O)O)O)OC(=O)C4=CC(=C(C(=C4C5=C(C(=C(C=C5C(=O)O1)O)O)O)O)O)O)O |  | -10.652 | (Kochumadhavan et al., 2019) |  |
|  | methylparaben | 7456 | COC(=O)C1=CC=C(C=C1)O |  | -10.638 | (Kuş et al., 2013) |  |
|  | alangiside | 442161 | COC1=C(C=C2C3CC4C(C(OC=C4C(=O)N3CCC2=C1)OC5C(C(C(C(O5)CO)O)O)O)C=C)O |  | -10.631 | (Tamaki et al., 2000) |  |
|  | 8'-α-hydroxyllariciresinol-4'-O-β-D-glucopyranoside |  |  |  | -10.630 | (Thompson et al., 1963) |  |
|  | alangimarckine | 442159 | CCC1CN2CCC3=C(C(=C(C=C3C2CC1CC4C5=C(CCN4)C6=CC=CC=C6N5)OC)OC)O |  | -10.621 | (Jain et al., 2002) |  |
|  | Olean-12-ene-28-carboxy-3-β-hexadecanoate |  |  |  | -10.619 | (Mallavadhani et al., 2003) |  |
|  | Epi-syringaresinol-4''-O-β-D-glucopyranoside |  |  |  | -10.618 | (Wang Changzeng & Jia Zhongjian, 1997) |  |
|  | Fumaric acid | 444972 | C(=CC(=O)O)C(=O)O |  | -10.615 | (Kloetzer et al., 2019) |  |
|  | Diosgenin acetate | 101952 | CC1CCC2(C(C3C(O2)CC4C3(CCC5C4CC=C6C5(CCC(C6)OC(=O)C)C)C)C)OC1 |  | -10.598 | (Pazhanichamy et al., 2012) |  |
|  | vanillin | 1183 | COC1=C(C=CC(=C1)C=O)O |  | -10.554 | (Walton et al., 2003) |  |
|  | alangidiol | 101277332 | CC(C)C1CCC2(C1(CCC3(C2CCC4C3(CCC5C4(CCC(C5(C)C)O)C)C)C)C)O |  | -10.554 | (Ogunkoya, 1978) |  |
|  | β-Sitosterol | 222284 | CCC(CCC(C)C1CCC2C1(CCC3C2CC=C4C3(CCC(C4)O)C)C)C(C)C |  | -10.537 | (Gupta et al., 1980) |  |
|  | B-Sitostenone | 579897 | CCC(CCC(C)C1CCC2C1(CCC3C2CCC4=CC(=O)CCC34C)C)C(C)C |  | -10.519 | (Huyen et al., 2021) |  |
|  | Phyllanemblinin A | 11135859 | C1=C(C=C(C(=C1O)O)O)C(=O)OC2C3C(C(C(O2)CO)OC(=O)C4=CC(=C(C5=C4C6=C(O5)C(=C(C=C6C(=O)O3)O)O)O)O)O |  | -10.496 | (Y.-J. Zhang et al., 2001) |  |
|  | cephaeline | 442195 | CCC1CN2CCC3=CC(=C(C=C3C2CC1CC4C5=CC(=C(C=C5CCN4)O)OC)OC)OC |  | -10.492 | (Habib & Harkiss, 2011) |  |
|  | monolinolein | 5283469 | CCCCCC=CCC=CCCCCCCCC(=O)OCC(CO)O |  | -10.466 | (Stoessl et al., 1980) |  |
|  | chebulinic acid | 72284 | C1=C(C=C(C(=C1O)O)O)C(=O)OCC2C3C(C(C(O2)OC(=O)C4=CC(=C(C(=C4)O)O)O)OC(=O)C5=CC(=C(C6=C5C(C(C(=O)O3)CC(=O)O)C(C(=O)O6)O)O)O)OC(=O)C7=CC(=C(C(=C7)O)O)O |  | -10.433 | (YOSHIDA et al., 1980) |  |
|  | salviifosides B |  |  |  | -10.422 | (Hung et al., 2009) |  |
|  | Erythrodiol-3-O-palmitate | 124222344 | CCCCCCCCCCCCCCCC(=O)OC1CCC2(C(C1(C)C)CCC3(C2CC=C4C3(CCC5(C4CC(CC5)(C)C)CO)C)C)C |  | -10.419 | (F. Wang & Li, 2010) |  |
|  | 3-O-Demethyl swertipunicoside | 10372399 | C1=C(C=C2C(=C1O)C(=O)C3=C(C(=CC(=C3O2)O)C4=C5C(=C(C(=C4O)C6C(C(C(C(O6)CO)O)O)O)O)C(=O)C7=CC(=C(C=C7O5)O)O)O)O |  | -10.417 | (Du et al., 2012) |  |
|  | 9, 10-methylene octadec-9-enoic(sterculic) acid | 11426463 | CCCCCCCCC1CC1CCCCCCCC(=O)O |  | -10.399 | (Roomi & Hopkins, 1970) |  |
|  | Sitogluside | 5742590 | CCC(CCC(C)C1CCC2C1(CCC3C2CC=C4C3(CCC(C4)OC5C(C(C(C(O5)CO)O)O)O)C)C)C(C)C |  | -10.396 | (Guo et al., 2021) |  |
|  | Amaroswerin | 45359883 | C=CC1C(OC=C2C1(CCOC2=O)O)OC3C(C(C(C(O3)CO)O)O)OC(=O)C4=C(C=C(C=C4O)O)C5=CC(=CC=C5)O |  | -10.393 | (Narasimha Rao et al., 1998) |  |
|  | deoxytubulosine | 165003 | CCC1CN2CCC3=CC(=C(C=C3C2CC1CC4C5=C(CCN4)C6=CC=CC=C6N5)OC)OC |  | -10.371 | (Narasimha Rao et al., 1998) |  |
|  | 6′  -(stigmast-5-en-3-O-β-D-glucopyranosidyl) hexadecanoate | 146156493 | CCC(CCC(C)C1CCC2C1(CCC3C2CC(C4=CC(CCC34C)O)O)C)C(C)C |  | -10.337 | (Irsyam et al., 2020) |  |
|  | Syringetin 3-O-glucoside | 5321577 | COC1=CC(=CC(=C1O)OC)C2=C(C(=O)C3=C(C=C(C=C3O2)O)O)OC4C(C(C(C(O4)CO)O)O)O |  | -10.320 | (Mattivi et al., 2006) |  |
|  | Higenamine 4'-O-β-D-glucoside |  |  |  | -10.316 | (Kato et al., 2017) |  |
|  | chebulanin | 75034370 | C1=C(C=C(C(=C1O)O)O)C(=O)OC2C3C(C(C(O2)CO)OC(=O)C(C4C(C(=O)OC5=C4C(=CC(=C5O)O)C(=O)O3)O)CC(=O)O)O |  | -10.304 | (Bag et al., 2013) |  |
|  | chrysoeriol 7-O-glucopyranoside |  |  |  | -10.295 | (Dej-adisai et al., 2018) |  |
|  | Myricetin 3',5'-dimethylether 3-O-beta-D-glucopyranoside |  |  |  | -10.287 | (Kunanusorn et al., 2011) |  |
|  | (+)-Cycloolivil-4'-O-β-D-glucopyranoside |  |  |  | -10.263 | (S. Li et al., 2020) |  |
|  | salviifosides C |  |  |  | -10.248 | (Panara et al., 2016) |  |
|  | isocephaeline | 12302705 | CCC1CN2CCC3=CC(=C(C=C3C2CC1CC4C5=CC(=C(C=C5CCN4)O)OC)OC)OC |  | -10.213 | (Itoh et al., 1999) |  |
|  | Amaronitidin | 101720810 | C=CC1C(OC=C2C1=CCOC2=O)OC3C(C(C(C(O3)CO)O)O)OC4=C(C=C(C=C4O)O)C5=CC(=CC=C5)O |  | -10.201 | (KAWAHARA et al., 2001) |  |
|  | Stigmast-5-en-3-yl acetate | 521199 | CCC(CCC(C)C1CCC2C1(CCC3C2CC=C4C3(CCC(C4)OC(=O)C)C)C)C(C)C |  | -10.187 | (Ding et al., 2010) |  |
|  | isocorilagin | 10077799 | C1C2C(C(C(C(O2)OC(=O)C3=CC(=C(C(=C3)O)O)O)O)OC(=O)C4=CC(=C(C(=C4C5=C(C(=C(C=C5C(=O)O1)O)O)O)O)O)O)O |  | -10.184 | (Braca et al., 2002) |  |
|  | N-feruloyl tyrosine | 46865641 | COC1=C(C=CC(=C1)C=CC(=O)NC(CC2=CC=C(C=C2)O)C(=O)O)O |  | -10.169 | (Negrel & Martin, 1984) |  |
|  | Kaempferol 3-O-beta-D-galactopyranoside | 5282149 | C1=CC(=CC=C1C2=C(C(=O)C3=C(C=C(C=C3O2)O)O)OC4C(C(C(C(O4)CO)O)O)O)O |  | -10.103 | (Baba & Kashimawo, 2013) |  |
|  | 20, 23-Dimethylcholesta-6, 22-dien-3β-ol |  |  |  | -10.098 | (Gomaa et al., 2018) |  |
|  | Swertiachiridiol A |  |  |  | -10.095 | (Swati, Bhatt, Sendri, Bhatt, et al., 2023) |  |
|  | 4,4-Dimethyl-5a-cholesta-8,24-dien-3-b-ol | 50990081 | CC(CCC=C(C)C)C1CCC2C1(CCC3=C2CCC4C3(CCC(C4(C)C)O)C)C |  | -10.023 | (Smith & Goad, 1971) |  |
|  | Myricetin 3-O-glucoside | 5318606 | C1=C(C=C(C(=C1O)O)O)C2=C(C(=O)C3=C(C=C(C=C3O2)O)O)OC4C(C(C(C(O4)CO)O)O)O |  | -10.017 | (Razavi et al., 2009) |  |
|  | 4-hydroxy-4-methyl-2 pentanone | 31256 | CC(=O)CC(C)(C)O |  | -10.002 | (Kamar et al., 1986) |  |
|  | 7-O-[β-D-xylopyranosyl-(1→2)-β-D-xylopyranosyl]-1,8-dihydroxy-3-methoxy xanthone |  |  |  | -9.972 | (Yin et al., 2014) |  |
|  | 10-Eicosanol | 3904033 | CCCCCCCCCCC(CCCCCCCCC)O |  | -9.960 | (Agnihotri et al., 2008) |  |
|  | Isorhamnetin 3-O-β-D-glucopyranoside |  |  |  | -9.958 | (Kokubo et al., 1991) |  |
|  | 8'-α-hydroxyllariciresinol-4-O-β-D-glucopyranoside |  |  |  | -9.952 | (J. Li et al., 2015) |  |
|  | 2,6-Dimethoxy-1,4 benzoquinone | 68262 | COC1=CC(=O)C=C(C1=O)OC |  | -9.925 | (Harasawa & Tagashira, 1994) |  |
|  | Kaempferol 3-O-beta-D-glucuronopyranosyl  methylester |  |  |  | -9.916 | (Kunanusorn et al., 2011) |  |
|  | 1,2-Benzenedicarboxylic acid, diisooctyl ester | 33934 | CC(C)CCCCCOC(=O)C1=CC=CC=C1C(=O)OCCCCCC(C)C |  | -9.891 | (MOHAMED ZAKY ZAYED et al., 2014) |  |
|  | 6'-O-β-D-glucopyranosyl gentiopicroside |  |  |  | -9.867 | (Xu et al., 2009) |  |
|  | 9E,12E,15E-Octadecatrienoic acid | 5282822 | CCC=CCC=CCC=CCCCCCCCC(=O)O |  | -9.814 | (Chaudhuri & Singh, 2009) |  |
|  | Amarogentin | 115149 | C=CC1C2CCOC(=O)C2=COC1OC3C(C(C(C(O3)CO)O)O)OC(=O)C4=C(C=C(C=C4O)O)C5=CC(=CC=C5)O |  | -9.811 | (Ray et al., 1996) |  |
|  | Moretenol | 12309610 | CC(=C)C1CCC2(C1CCC3(C2CCC4C3(CCC5C4(CCC(C5(C)C)O)C)C)C)C |  | -9.802 | (Galbraith et al., 1965) |  |
|  | 1,6-di-O-galloyl- β-D-glucose | 440221 | C1=C(C=C(C(=C1O)O)O)C(=O)OCC2C(C(C(C(O2)OC(=O)C3=CC(=C(C(=C3)O)O)O)O)O)O |  | -9.791 | (Gross & Denzel, 1990) |  |
|  | Isoquercetin | 5280804 | C1=CC(=C(C=C1C2=C(C(=O)C3=C(C=C(C=C3O2)O)O)OC4C(C(C(C(O4)CO)O)O)O)O)O |  | -9.753 | (Jayachandran et al., 2018) |  |
|  | 3-hydroxy-β-ionol | 5352752 | CC1=C(C(CC(C1)O)(C)C)C=CC(C)O |  | -9.723 | (Fujimori et al., 1975) |  |
|  | Stigmasterol | 5280794 | CCC(C=CC(C)C1CCC2C1(CCC3C2CC=C4C3(CCC(C4)O)C)C)C(C)C |  | -9.703 | (Nair et al., 1984) |  |
|  | Immunoside / sarsasapogenin | 92095 | CC1CCC2(C(C3C(O2)CC4C3(CCC5C4CCC6C5(CCC(C6)O)C)C)C)OC1 |  | -9.702 | (Sidiq et al., 2011) |  |
|  | VNI [(R)-N-(1-(2,4-dichlorophenyl)-2-(1H-imidazol-1-yl)ethyl)-4-(5-phenyl-1,3,4-oxadiazol-2-yl)benzamide)] |  |  |  | -9.691 | (Shamsuddin et al., 2021) |  |
|  | Quercetin 3-O-b-D-glucuronide | 5274585 | C1=CC(=C(C=C1C2=C(C(=O)C3=C(C=C(C=C3O2)O)O)OC4C(C(C(C(O4)C(=O)O)O)O)O)O)O |  | -9.605 | (Hiermann et al., 1998) |  |
|  | 4-hydroxyacetophenone | 7469 | CC(=O)C1=CC=C(C=C1)O |  | -9.581 | (Bernardes et al., 2008) |  |
|  | trans-Phytol | 5280435 | CC(C)CCCC(C)CCCC(C)CCCC(=CCO)C |  | -9.571 | (B. Singh et al., 1991) |  |
|  | α-Mangostin | 5281650 | CC(=CCC1=C(C2=C(C=C1O)OC3=C(C2=O)C(=C(C(=C3)O)OC)CC=C(C)C)O)C |  | -9.550 | (Chairungsrilerd et al., 1996) |  |
|  | Chiratenol | 14831162 | CC1(CCC2(C3CCC4C5(CCC(C(C5CCC4(C3(CC=C2C1)C)C)(C)C)O)C)C)C |  | -9.538 | (Kumar Chakravarty et al., 1990) |  |
|  | Chirat-16-en-3β-24-diol |  |  |  | -9.534 | (A.K., 2001) |  |
|  | Maslinic acid | 73659 | CC1(CCC2(CCC3(C(=CCC4C3(CCC5C4(CC(C(C5(C)C)O)O)C)C)C2C1)C)C(=O)O)C |  | -9.528 | (Lozano-Mena et al., 2014) |  |
|  | Stigmasta-4,22-dien-3-one | 6442194 | CCC(C=CC(C)C1CCC2C1(CCC3C2CCC4=CC(=O)CCC34C)C)C(C)C |  | -9.517 | (HAYASHI et al., 1969) |  |
|  | 6'-O-β-D-glucopyranosyl sweroside |  |  |  | -9.516 | (Xu et al., 2009) |  |
|  | 3-hydroxy-β-damascone | 5366075 | CC=CC(=O)C1=C(C(CCC1(C)C)O)C |  | -9.469 | (Gerhäuser et al., 2009) |  |
|  | stigmast-4-en-3,6-dione | 146158313 | CCC(CCC(C)C1CCC2C1(CCC3C2CC(=O)C4=CC(=O)CCC34C)C)C(C)C |  | -9.468 | (HAYASHI et al., 1969) |  |
|  | Kaempferol 7-O-beta-D-glucopyranoside | 10095180 | C1=CC(=CC=C1C2=C(C(=O)C3=C(C=C(C=C3O2)OC4C(C(C(C(O4)CO)O)O)O)O)O)O |  | -9.451 | (HAYASHI et al., 1969) |  |
|  | Tannic acid | 16129778 | C1=C(C=C(C(=C1O)O)O)C(=O)OC2=CC(=CC(=C2O)O)C(=O)OCC3C(C(C(C(O3)OC(=O)C4=CC(=C(C(=C4)OC(=O)C5=CC(=C(C(=C5)O)O)O)O)O)OC(=O)C6=CC(=C(C(=C6)OC(=O)C7=CC(=C(C(=C7)O)O)O)O)O)OC(=O)C8=CC(=C(C(=C8)OC(=O)C9=CC(=C(C(=C9)O)O)O)O)O)OC(=O)C1=CC(=C(C(=C1)OC(=O)C1=CC(=C(C(=C1)O)O)O)O)O |  | -9.450 | (Mitjavila et al., 1977) |  |
|  | Nonadecane | 12401 | CCCCCCCCCCCCCCCCCCC |  | -9.440 | (Zerbi et al., 1981) |  |
|  | Methyl (3β)-3-hydroxyurs-12-en-28-oate |  |  |  | -9.419 | (S.-J. Chang et al., 2001) |  |
|  | Olean-12-en-18αH-3-one |  |  |  | -9.403 | (Šmelcerović et al., 2017) |  |
|  | Stigmast-4-en-3-one | 5484202 | CCC(CCC(C)C1CCC2C1(CCC3C2CCC4=CC(=O)CCC34C)C)C(C)C |  | -9.396 | (S.-J. Chang et al., 2001) |  |
|  | Olean-12-ene-18αH-3-one-19β-ol |  |  |  | -9.372 | (Šmelcerović et al., 2017) |  |
|  | Myricetin 3-O-galactoside | 5491408 | C1=C(C=C(C(=C1O)O)O)C2=C(C(=O)C3=C(C=C(C=C3O2)O)O)OC4C(C(C(C(O4)CO)O)O)O |  | -9.364 | (Romani et al., 1999) |  |
|  | Ψ-Taraxasterol or heterolupeol | 604983 | CC1C2C3CCC4C5(CCC(C(C5CCC4(C3(CCC2(CC=C1C)C)C)C)(C)C)O)C |  | -9.342 | (Halsall et al., 1954) |  |
|  | Cholest-4-en 3-one | 91477 | CC(C)CCCC(C)C1CCC2C1(CCC3C2CCC4=CC(=O)CCC34C)C |  | -9.339 | (S.-C. Lee et al., 2010) |  |
|  | diosgenin | 99474 | CC1CCC2(C(C3C(O2)CC4C3(CCC5C4CC=C6C5(CCC(C6)O)C)C)C)OC1 |  | -9.337 | (J Shah, 2012) |  |
|  | reserpiline | 67228 | CC1C2CN3CCC4=C(C3CC2C(=CO1)C(=O)OC)NC5=CC(=C(C=C45)OC)OC |  | -9.324 | (Maurya et al., 2013) |  |
|  | Quercetin 3-glucuronide | 12004528 | C1=CC(=C(C=C1C2=C(C(=O)C3=C(C=C(C=C3O2)O)O)OC4C(C(C(C(O4)C(=O)O)O)O)O)O)O |  | -9.308 | (Dueñas et al., 2008) |  |
|  | Isoorientin | 114776 | C1=CC(=C(C=C1C2=CC(=O)C3=C(O2)C=C(C(=C3O)C4C(C(C(C(O4)CO)O)O)O)O)O)O |  | -9.304 | (Zeraik & Yariwake, 2010) |  |
|  | 10-Octadecenoic acid, methyl ester | 5364425 | CCCCCCCC=CCCCCCCCCC(=O)OC |  | -9.261 | (Rodríguez et al., 2001) |  |
|  | Cyanidine 3-O-galactoside | 441699 | C1=CC(=C(C=C1C2=[O+]C3=CC(=CC(=C3C=C2OC4C(C(C(C(O4)CO)O)O)O)O)O)O)O |  | -9.242 | (Ozga et al., 2007) |  |
|  | salviifosides A |  |  |  | -9.219 | (Ozga et al., 2007) |  |
|  | Isophytol | 10453 | CC(C)CCCC(C)CCCC(C)CCCC(C)(C=C)O |  | -9.189 | (Sato et al., 1963) |  |
|  | Myricetin 3-O-glucuronide | 5487413 | C1=C(C=C(C(=C1O)O)O)C2=C(C(=O)C3=C(C=C(C=C3O2)O)O)OC4C(C(C(C(O4)C(=O)O)O)O)O |  | -9.162 | (Barbosa et al., 2006) |  |
|  | Astragalin | 5282102 | C1=CC(=CC=C1C2=C(C(=O)C3=C(C=C(C=C3O2)O)O)OC4C(C(C(C(O4)CO)O)O)O)O |  | -9.151 | (Kotani et al., 2000) |  |
|  | Asparagamine-A |  |  |  | -9.141 | (Ikegami, 2005) |  |
|  | 12-Hydroxyoleanolic lactone |  |  |  | -9.124 | (Barton & Holness, 1952) |  |
|  | Taraxerol | 92097 | CC1(CCC2(CC=C3C4(CCC5C(C(CCC5(C4CCC3(C2C1)C)C)O)(C)C)C)C)C |  | -9.077 | (Komai et al., 2006) |  |
|  | Benzene, 1, 3-dimethyl m xylene | 7929 | CC1=CC(=CC=C1)C |  | -9.070 | (Temitayo Oyekunle & Temitayooyekunle, 2017) |  |
|  | purpactin A | 10341722 | CC1=CC2=C(C(=C1)O)OC3=C(C(=C(C=C3)C(CC(C)C)OC(=O)C)OC)C(=O)OC2 |  | -9.054 | (Komai et al., 2006) |  |
|  | 7,11,15-Trimethylhexadecan-2-one | 14718575 | CC(C)CCCC(C)CCCC(C)CCCCC(=O)C |  | -9.051 | (Rontani et al., 2002) |  |
|  | 3,6-di-O-galloyl-D-glucose | 129852263 | C1=C(C=C(C(=C1O)O)O)C(=O)OCC(C(C(C(C=O)O)OC(=O)C2=CC(=C(C(=C2)O)O)O)O)O |  | -9.040 | (Nishira & Joslyn, 1968) |  |
|  | Oleic acid | 445639 | CCCCCCCCC=CCCCCCCCC(=O)O |  | -9.013 | (Jassal et al., 1994) |  |
|  | Kairatenol | 102285188 | CC1(CCCC2C1(CCC3(C2=CCC4C3(CCC5C4(CCC(C5(C)C)O)C)C)C)C)C |  | -8.987 | (Tra et al., 2022) |  |
|  | betulinaldehyde | 99615 | CC(=C)C1CCC2(C1C3CCC4C5(CCC(C(C5CCC4(C3(CC2)C)C)(C)C)O)C)C=O |  | -8.976 | (Yan et al., 2011) |  |
|  | Kaempferol 3-O-beta-D-glucuronopyranoside | 5318759 | C1=CC(=CC=C1C2=C(C(=O)C3=C(C=C(C=C3O2)O)O)OC4C(C(C(C(O4)C(=O)O)O)O)O)O |  | -8.966 | (Yan et al., 2011) |  |
|  | Abutilin A |  |  |  | -8.962 | (P.-C. Kuo et al., 2008) |  |
|  | Luteolin/luteolin glucoside | 49852298 | C1=CC(=C(C=C1C2=CC(=O)C3=C(O2)C=C(C(=C3[O-])C4C(C(C(C(O4)CO)O)O)O)O)O)O |  | -8.947 | (C. Hu & Kitts, 2004) |  |
|  | 1-O-β-D-glucopyranosyl-3,5,8-trihydroxy xanthone |  |  |  | -8.927 | (Bannon et al., 1973) |  |
|  | Methyl (3β)-3-hydroxyolean-12-en-28-oate |  |  |  | -8.912 | (Hart et al., 1976) |  |
|  | beta-Sitosterol acetate | 5354503 | CCC(CCC(C)C1CCC2C1(CCC3C2CC=C4C3(CCC(C4)OC(=O)C)C)C)C(C)C |  | -8.885 | (Hidayathulla et al., 2018) |  |
|  | Swertianolin | 5281662 | COC1=CC(=C2C(=C1)OC3=C(C=CC(=C3C2=O)OC4C(C(C(C(O4)CO)O)O)O)O)O |  | -8.862 | (Wei et al., 2018) |  |
|  | Isovitexin | 162350 | C1=CC(=CC=C1C2=CC(=O)C3=C(O2)C=C(C(=C3O)C4C(C(C(C(O4)CO)O)O)O)O)O |  | -8.848 | (Fu et al., 2008) |  |
|  | Hyperoside | 5281643 | C1=CC(=C(C=C1C2=C(C(=O)C3=C(C=C(C=C3O2)O)O)OC4C(C(C(C(O4)CO)O)O)O)O)O |  | -8.799 | (X. Zhou et al., 2008) |  |
|  | Ursolic acid | 64945 | CC1CCC2(CCC3(C(=CCC4C3(CCC5C4(CCC(C5(C)C)O)C)C)C2C1C)C)C(=O)O |  | -8.783 | (Vetal et al., 2012) |  |
|  | Nelumnucifoside B | 154814078 | CC1=C2CC(CCC2(CCC1=O)C)C(C)(C)OC3C(C(C(C(O3)CO)O)O)O |  | -8.771 | (Ahn et al., 2013a) |  |
|  | Ankorine | 442166 | CCC1CN2CCC3=C(C(=C(C=C3C2CC1CCO)OC)OC)O |  | -8.756 | (Dasgupta, 1965) |  |
|  | Nelumnucifoside A |  |  |  | -8.754 | (Lim, 2016) |  |
|  | lirioresinol A/ Syringaresinol | 100067 | COC1=CC(=CC(=C1O)OC)C2C3COC(C3CO2)C4=CC(=C(C(=C4)OC)O)OC |  | -8.747 | (Battersby et al., 1968) |  |
|  | p-hydroxybenzaldehyde | 126 | C1=CC(=CC=C1C=O)O |  | -8.743 | (Carnero Ruiz et al., 1990) |  |
|  | Yohimbine | 8969 | COC(=O)C1C(CCC2C1CC3C4=C(CCN3C2)C5=CC=CC=C5N4)O |  | -8.729 | (Betz et al., 1995) |  |
|  | N-Trans-feruloyltramine | 5280537 | COC1=C(C=CC(=C1)C=CC(=O)NCCC2=CC=C(C=C2)O)O |  | -8.707 | (Kanada et al., 2012) |  |
|  | Pentadecenoic acid, 14-methyl-methyl ester |  |  |  | -8.704 | (Abe et al., 2006) |  |
|  | Deacetylcentapicrin |  |  |  | -8.688 | (Ghosh et al., 2021) |  |
|  | Methyl indole-3-carboxylate | 589098 | COC(=O)C1=CNC2=CC=CC=C21 |  | -8.625 | (S.-C. Hu et al., 2005) |  |
|  | (3β,13α,14β,20α)-3-hydroxy-13-methyl-26-norolean-8-en-29-oic acid |  |  |  | -8.613 | (Pant et al., 2003) |  |
|  | Norswertianolin | 5281659 | C1=CC(=C2C(=C1O)OC3=CC(=CC(=C3C2=O)O)O)OC4C(C(C(C(O4)CO)O)O)O |  | -8.591 | (SAKAMOTO et al., 1982) |  |
|  | Benzene, 1, 3-dimethyl P xylene | 7809 | CC1=CC=C(C=C1)C |  | -8.585 | (Temitayo Oyekunle & Temitayooyekunle, 2017) |  |
|  | Digalloylglucose | 129628549 | C1=C(C=C(C(=C1O)O)O)C(=O)C(C(C(C(C(C=O)O)O)O)O)(C(=O)C2=CC(=C(C(=C2)O)O)O)O |  | -8.569 | (Gross & Denzel, 1991) |  |
|  | 4-ketopinoresinol | 44578390 | COC1=C(C=CC(=C1)C2C3COC(C3C(=O)O2)C4=CC(=C(C=C4)O)OC)O |  | -8.568 | (Tra et al., 2022) |  |
|  | 3-β-Hydroxy-11-oxo-olean-12-enyl-3-palmitate |  |  |  | -8.553 | (K.-W. Wang, 2007) |  |
|  | Oleanolic acid | 10494 | CC1(CCC2(CCC3(C(=CCC4C3(CCC5C4(CCC(C5(C)C)O)C)C)C2C1)C)C(=O)O)C |  | -8.532 | (Pollier & Goossens, 2012) |  |
|  | indobinine |  |  |  | -8.505 | (Grewal et al., 2020) |  |
|  | 9-cis-Retinal | 6436082 | CC1=C(C(CCC1)(C)C)C=CC(=CC=CC(=CC=O)C)C |  | -8.464 | (Popp et al., 1993) |  |
|  | Icariside B2 | 10385469 | CC(=O)C=CC12C(CC(CC1(O2)C)OC3C(C(C(C(O3)CO)O)O)O)(C)C |  | -8.448 | (Hartleb & Seifert, 1994) |  |
|  | Bridelionoside B | 53326026 | CC(C=CC1(C(CC(CC1(C)O)O)(C)C)O)OC2C(C(C(C(O2)CO)O)O)O |  | -8.432 | (Sueyoshi et al., 2006) |  |
|  | Racemosol | 624971 | CC1=C(C=C2CCC3=C(C=CC(=C3O)OC)C4C2=C1OC(C4)(C)C)O |  | -8.422 | (Sekine et al., 1997) |  |
|  | Swertiachoside A |  |  |  | -8.421 | (N.-J. Zhou et al., 2015) |  |
|  | 4-Methyl-N-methylcoclaurine |  |  |  | -8.362 | (Girisham et al., 1986) |  |
|  | Swertiachoside B (3-nortetrahydroswertianolin) | 168011885 | C1CC(C2=C(C1O)OC3CC(CC(C3C2=O)O)O)OC4C(C(C(C(O4)CO)O)O)O |  | -8.352 | (S. Yang et al., 2018) |  |
|  | Swertenol | 21726415 | CC1(CCCC2C1(CCC3(C2(CCC4C3=CCC5C4(CCC(C5(C)C)O)C)C)C)C)C |  | -8.349 | (Chakravarty, Mukhopadhyay, et al., 1991b) |  |
|  | Episwertenol | 101619548 | CC1(CCCC2C1(CCC3(C2(CCC4C3=CCC5C4(CCC(C5(C)C)O)C)C)C)C)C |  | -8.348 | (Chakravarty, Mukhopadhyay, et al., 1991b) |  |
|  | mucic acid 1-ethyl ester 3-O-gallate |  |  |  | -8.322 | (Sun et al., 2023) |  |
|  | Serpentine | 73391 | CC1C2C[N+]3=C(CC2C(=CO1)C(=O)OC)C4=C(C=C3)C5=CC=CC=C5N4 |  | -8.313 | (Proctor & Woodell, 1975) |  |
|  | Isomangostin | 5281641 | CC(=CCC1=C(C(=CC2=C1C(=O)C3=C(O2)C=C(C4=C3OC(CC4)(C)C)O)O)OC)C |  | -8.308 | (Quan et al., 2010) |  |
|  | Swertanone | 102285187 | CC1(CCCC2C1(CCC3(C2(CCC4C3=CCC5C4(CCC(=O)C5(C)C)C)C)C)C)C |  | -8.304 | (Chakravarty, Das, et al., 1991) |  |
|  | 4-epi-hederagenin | 73299 | CC1(CCC2(CCC3(C(=CCC4C3(CCC5C4(CCC(C5(C)CO)O)C)C)C2C1)C)C(=O)O)C |  | -8.252 | (Cantrell et al., 2003) |  |
|  | 7-Hydroxydehydroglaucine | 10317002 | CN1CCC2=CC(=C(C3=C2C1=C(C4=CC(=C(C=C43)OC)OC)O)OC)OC |  | -8.251 | (Mukusheva et al., 2011) |  |
|  | Byzantionoside A | 73345910 | CC1=CC(CC(C1C=CC(=O)C)(C)C)OC2C(C(C(C(O2)CO)O)O)O |  | -8.250 | (W. Wang et al., 2009) |  |
|  | Methyl (3β)-3-hydroxyolean-12,15-dien-28-oate |  |  |  | -8.245 | (Borges et al., 2009) |  |
|  | 1-Hydroxy-2,3,4,7-tetramethoxy xanthone | 5318358 | COC1=CC2=C(C=C1)OC3=C(C(=C(C(=C3C2=O)O)OC)OC)OC |  | -8.210 | (Dhasmana & Garg, 1989) |  |
|  | α-amyrin | 73170 | CC1CCC2(CCC3(C(=CCC4C3(CCC5C4(CCC(C5(C)C)O)C)C)C2C1C)C)C |  | -8.163 | (Nnamonu et al., 2016) |  |
|  | Isomangiferin | 5318597 | C1=C2C(=CC(=C1O)O)OC3=C(C2=O)C(=CC(=C3C4C(C(C(C(O4)CO)O)O)O)O)O |  | -8.157 | (Kitanov & Nedialkov, 1998) |  |
|  | isolariciresinol | 160521 | COC1=C(C=C2C(C(C(CC2=C1)CO)CO)C3=CC(=C(C=C3)O)OC)O |  | -8.154 | (Meagher et al., 1999) |  |
|  | 4-O-β-Glucosylbenzoic acid | 440186 | C1=CC(=CC=C1C(=O)O)OC2C(C(C(C(O2)CO)O)O)O |  | -8.132 | (Jaiswal et al., 2014) |  |
|  | (E)-Sinapate-4-O-b-D-glucopyranoside |  |  |  | -8.125 | (Rho & Yoon, 2017) |  |
|  | Alangine | 10851977 | COC1=C(C=C2C3CC(CCN3CCC2=C1)C(CO)C=C)O |  | -8.124 | (Bal & Dutt, 1946) |  |
|  | 1-Hydroxy3,5,7,8-tetramethoxy xanthone | 10065320 | COC1=CC(=C2C(=C1)OC3=C(C2=O)C(=C(C=C3OC)OC)OC)O |  | -8.119 | (Siriwardhana et al., 2015) |  |
|  | cis-N-Feruloyltyramine /cis-N-Coumaroyltyramine | 6440659 | COC1=C(C=CC(=C1)C=CC(=O)NCCC2=CC=C(C=C2)O)O |  | -8.113 | (D. K. Kim et al., 2005) |  |
|  | (-)-1(R)-N-methylcoclaurine | 440595 | CN1CCC2=CC(=C(C=C2C1CC3=CC=C(C=C3)O)O)OC |  | -8.104 | (K.-B. Choi et al., 2001) |  |
|  | 1-Hydroxy-2,3,4,6-tetramethoxy xanthone | 11522801 | COC1=CC2=C(C=C1)C(=O)C3=C(C(=C(C(=C3O2)OC)OC)OC)O |  | -8.078 | (Siriwardhana et al., 2015) |  |
|  | Medioresinol | 181681 | COC1=CC(=CC(=C1O)OC)C2C3COC(C3CO2)C4=CC(=C(C=C4)O)OC |  | -8.069 | (DEYAMA, 1983) |  |
|  | Mangiferin | 5281647 | C1=C2C(=CC(=C1O)O)OC3=C(C2=O)C(=C(C(=C3)O)C4C(C(C(C(O4)CO)O)O)O)O |  | -8.064 | (Kulkarni & Rathod, 2014) |  |
|  | Betulinic acid | 64971 | CC(=C)C1CCC2(C1C3CCC4C5(CCC(C(C5CCC4(C3(CC2)C)C)(C)C)O)C)C(=O)O |  | -8.055 | (G. Zhao et al., 2007) |  |
|  | Salicin | 439503 | C1=CC=C(C(=C1)CO)OC2C(C(C(C(O2)CO)O)O)O |  | -8.054 | (Minakhmetov et al., 2002) |  |
|  | Dehydronuciferine | 821347 | CN1CCC2=CC(=C(C3=C2C1=CC4=CC=CC=C43)OC)OC |  | -8.034 | (Saa & Cava, 1977) |  |
|  | Friedelin | 91472 | CC1C(=O)CCC2C1(CCC3C2(CCC4(C3(CCC5(C4CC(CC5)(C)C)C)C)C)C)C |  | -8.029 | (de Vasconcelos et al., 2000) |  |
|  | Phyllaemblinol | 9842429 | CC1=CC2=C(C(=C1)O)OC3=C(C(=C(C=C3)C(CC(C)C)O)OC)C(=O)OC2 |  | -8.028 | (Kaur, 2022) |  |
|  | Benzene, 1, 3-dimethyl O xylene | 7237 | CC1=CC=CC=C1C |  | -8.022 | (Temitayo Oyekunle & Temitayooyekunle, 2017) |  |
|  | 2-epi-corosolic acid | 24721200 | CC1CCC2(CCC3(C(=CCC4C3(CCC5C4(CC(C(C5(C)C)O)O)C)C)C2C1C)C)C(=O)O |  | -7.995 | (Sommerwerk et al., 2016) |  |
|  | Erythrodiol | 101761 | CC1(CCC2(CCC3(C(=CCC4C3(CCC5C4(CCC(C5(C)C)O)C)C)C2C1)C)CO)C |  | -7.987 | (Amelio et al., 1992) |  |
|  | β-amyrin | 73145 | CC1(CCC2(CCC3(C(=CCC4C3(CCC5C4(CCC(C5(C)C)O)C)C)C2C1)C)C)C |  | -7.987 | (Simándi et al., 2002) |  |
|  | hexahydrofarnesyl acetone | 10408 | CC(C)CCCC(C)CCCC(C)CCCC(=O)C |  | -7.972 | (Balogun et al., 2017) |  |
|  | (-)-Nuciferine | 10146 | CN1CCC2=CC(=C(C3=C2C1CC4=CC=CC=C43)OC)OC |  | -7.968 | (Luo et al., 2005) |  |
|  | Ajmalicine | 441975 | CC1C2CN3CCC4=C(C3CC2C(=CO1)C(=O)OC)NC5=CC=CC=C45 |  | -7.947 | (Zenk, 1980) |  |
|  | Coclaurine | 160487 | COC1=C(C=C2C(NCCC2=C1)CC3=CC=C(C=C3)O)O |  | -7.936 | (Finkelstein, 1951) |  |
|  | indobine |  |  |  | -7.931 | (Siddiqui, Haider, et al., 1987b) |  |
|  | Lupeol | 259846 | CC(=C)C1CCC2(C1C3CCC4C5(CCC(C(C5CCC4(C3(CC2)C)C)(C)C)O)C)C |  | -7.931 | (Moriarty et al., 1998) |  |
|  | Neolancerin | 92029590 | C1=CC2=C(C=C1O)C(=O)C3=C(O2)C=C(C(=C3O)C4C(C(C(C(O4)CO)O)O)O)O |  | -7.901 | (Schaufelberger & Hostettmann, 1988) |  |
|  | N-Norarmepavine | 6999736 | COC1=C(C=C2C(NCCC2=C1)CC3=CC=C(C=C3)O)OC |  | -7.900 | (T. Yang et al., 1972) |  |
|  | Armepavine | 442169 | CN1CCC2=CC(=C(C=C2C1CC3=CC=C(C=C3)O)OC)OC |  | -7.873 | (Phillipson et al., 1981) |  |
|  | (6R,7E,9R)-9-hydroxymegastigma-4,7-dien-3-one-9-O-β-D-glucopyranoside |  |  |  | -7.872 | (In et al., 2014) |  |
|  | isoalanto‐lactone | 73285 | CC12CCCC(=C)C1CC3C(C2)OC(=O)C3=C |  | -7.833 | (Ketai, 2000) |  |
|  | Sweroside | 161036 | C=CC1C2CCOC(=O)C2=COC1OC3C(C(C(C(O3)CO)O)O)O |  | -7.825 | (Baba & Kashimawo, 2013) |  |
|  | 8, 9-methylene-heptadec-8-enoic (malvalic) acid | 10416 | CCCCCCCCC1=C(C1)CCCCCCC(=O)O |  | -7.814 | (Ralaimanarivo et al., 1982) |  |
|  | Swertiamarin | 442435 | C=CC1C(OC=C2C1(CCOC2=O)O)OC3C(C(C(C(O3)CO)O)O)O |  | -7.802 | (Vishwakarma et al., 2004) |  |
|  | Elephantorrhizol | 11988716 | C1C(C(OC2=C(C(=C(C(=C21)O)O)O)O)C3=CC(=C(C=C3)O)O)O |  | -7.784 | (Moyo et al., 1999) |  |
|  | 1-O-vanilloyl-beta-D-glucose | 14132344 | COC1=C(C=CC(=C1)C(=O)OC2C(C(C(C(O2)CO)O)O)O)O |  | -7.782 | (da Silva Oliveira et al., 2022) |  |
|  | alangimarine | 442160 | COC1=C(C=C2C(=C1)CCN3C2=CC4=C(C3=O)C=NC=C4C=C)O |  | -7.760 | (Nagarajan et al., 1994) |  |
|  | 1-Hydroxy-2,3,4,5-tetramethoxy xanthone | 5318357 | COC1=CC=CC2=C1OC3=C(C(=C(C(=C3C2=O)O)OC)OC)OC |  | -7.745 | (Y. Wang et al., 2009) |  |
|  | Ampicillin |  |  |  | -7.737 | (Shamsuddin et al., 2021) |  |
|  | Nuciferine N-Oxide | 71720427 | C[N+]1(CCC2=CC(=C(C3=C2C1CC4=CC=CC=C43)OC)OC)[O-] |  | -7.735 | (Z. Yang et al., 2014) |  |
|  | Norjuziphine | 15690955 | COC1=C(C2=C(CCNC2CC3=CC=C(C=C3)O)C=C1)O |  | -7.729 | (Hao & Qicheng, 1986) |  |
|  | 1,8-Dihydroxy-3,5,7-trimethoxy xanthone | 14779538 | COC1=CC(=C2C(=C1)OC3=C(C=C(C(=C3C2=O)O)OC)OC)O |  | -7.715 | (Markham, 1964) |  |
|  | (-)-Catechin | 73160 | C1C(C(OC2=CC(=CC(=C21)O)O)C3=CC(=C(C=C3)O)O)O |  | -7.699 | (Vuong et al., 2010) |  |
|  | Racemofuran |  |  |  | -7.691 | (Selvaraj et al., 2019) |  |
|  | Gentiopicroside | 88708 | C=CC1C(OC=C2C1=CCOC2=O)OC3C(C(C(C(O3)CO)O)O)O |  | -7.687 | (Popov et al., 1988) |  |
|  | mucic acid 6-methyl ester 3-O-gallate |  |  |  | -7.669 | (Sun et al., 2023) |  |
|  | Loganic acid | 89640 | CC1C(CC2C1C(OC=C2C(=O)O)OC3C(C(C(C(O3)CO)O)O)O)O |  | -7.658 | (Coscia et al., 1969) |  |
|  | Methylswertianin or Swertiaperennin | 5281653 | COC1=C(C2=C(C=C1)OC3=CC(=CC(=C3C2=O)O)OC)O |  | -7.647 | (KOMATSU et al., 1969) |  |
|  | 1-Hydroxy-3,5,8-trimethoxy xanthone |  |  |  | -7.618 | (Bennett et al., 1990) |  |
|  | Deoxyloganic acid | 443322 | CC1CCC2C1C(OC=C2C(=O)O)OC3C(C(C(C(O3)CO)O)O)O |  | -7.588 | (TAKEDA & INOUYE, 1976) |  |
|  | chebulagic acid | 442674 | C1C2C3C(C(C(O2)OC(=O)C4=CC(=C(C(=C4)O)O)O)OC(=O)C5=CC(=C(C6=C5C(C(C(=O)O3)CC(=O)O)C(C(=O)O6)O)O)O)OC(=O)C7=CC(=C(C(=C7C8=C(C(=C(C=C8C(=O)O1)O)O)O)O)O)O |  | -7.584 | (Han et al., 2006) |  |
|  | (-)-Lirinidine (5-demethylnuciferine) | 821343 | CN1CCC2=CC(=C(C3=C2C1CC4=CC=CC=C43)O)OC |  | -7.568 | (Ziyaev et al., 1973) |  |
|  | Isorhamnetin | 5281654 | COC1=C(C=CC(=C1)C2=C(C(=O)C3=C(C=C(C=C3O2)O)O)O)O |  | -7.561 | (Cao et al., 2008) |  |
|  | Acetic acid, 2oxo-5[(2-oxocyclohexyl)-phenyl-methyl]-pyrolidin-3yl ester |  |  |  | -7.534 | (Selin-Rani et al., 2016) |  |
|  | Chiratol / 1,5-Dihydroxy-3,8-dimethoxy xanthone | 10356746 | COC1=C2C(=C(C=C1)O)OC3=CC(=CC(=C3C2=O)O)OC |  | -7.496 | (Asthana et al., 1991) |  |
|  | d,l-Armepavine | 98348 | CN1CCC2=CC(=C(C=C2C1CC3=CC=C(C=C3)O)OC)OC |  | -7.495 | (Kupchan et al., 1969) |  |
|  | Lotusine | 5274587 | C[N+]1(CCC2=CC(=C(C=C2C1CC3=CC=C(C=C3)O)OC)O)C |  | -7.484 | (T. Yang & Chen, 1970a) |  |
|  | Taxifolin | 439533 | C1=CC(=C(C=C1C2C(C(=O)C3=C(C=C(C=C3O2)O)O)O)O)O |  | -7.479 | (H. Liu et al., 2010) |  |
|  | 3,4'-dihydroxy-3'-methoxypropiophenone-3-O-β-D-glucopyranoside |  |  |  | -7.475 | (Monte et al., 2001) |  |
|  | Mucic acid 2-gallate | 102039055 | C1=C(C=C(C(=C1O)O)O)C(=O)OC(C(C(C(C(=O)O)O)O)O)C(=O)O |  | -7.464 | (P.-H. Li et al., 2022) |  |
|  | Decussatin , (1-Hydroxy-3,7,8-trimethoxyxanthone | 5378284 | COC1=CC(=C2C(=C1)OC3=C(C=CC(=C3C2=O)O)O)O |  | -7.446 | (Fukamiya et al., 1990) |  |
|  | N-Methylisococlaurine | 21817819 | CN1CCC2=CC(=C(C=C2C1CC3=CC=C(C=C3)O)OC)O |  | -7.411 | (KAMETANI et al., 1968) |  |
|  | Nornuciferine | 41169 | COC1=C(C2=C3C(CC4=CC=CC=C42)NCCC3=C1)OC |  | -7.362 | (Luo et al., 2005) |  |
|  | N-Nornuciferine | 12313579 | COC1=C(C2=C3C(CC4=CC=CC=C42)NCCC3=C1)OC |  | -7.351 | (Luo et al., 2005) |  |
|  | Pronuciferine | 200480 | CN1CCC2=CC(=C(C3=C2C1CC34C=CC(=O)C=C4)OC)OC |  | -7.319 | (Bayazeid et al., 2018) |  |
|  | Kaempferol | 5280863 | C1=CC(=CC=C1C2=C(C(=O)C3=C(C=C(C=C3O2)O)O)O)O |  | -7.299 | (Watson & Oliveira, 1999) |  |
|  | Quercetin | 5280343 | C1=CC(=C(C=C1C2=C(C(=O)C3=C(C=C(C=C3O2)O)O)O)O)O |  | -7.263 | (M. Li & Xu, 2008) |  |
|  | Epitaxifolin | 443758 | C1=CC(=C(C=C1C2C(C(=O)C3=C(C=C(C=C3O2)O)O)O)O)O |  | -7.208 | (NONAKA et al., 1987) |  |
|  | 1(β)-O-Galloylglucose | 124375 | COC1C(C(C(C(O1)OC(=O)C2=CC(=C(C(=C2)O)O)O)O)O)O |  | -7.193 | (Cammann et al., 1989) |  |
|  | Cepharadione B | 189151 | CN1C2=CC3=CC=CC=C3C4=C2C(=CC(=C4OC)OC)C(=O)C1=O |  | -7.179 | (Jong & Jean, 1993) |  |
|  | 1-Hydroxy-3,7-dimethoxy xanthone | 5488808 | COC1=CC2=C(C=C1)OC3=CC(=CC(=C3C2=O)O)OC |  | -7.178 | (Jackson et al., 1967) |  |
|  | 5,7,3,5-Tetrahydroxyflavanone | 11483087 | C1C(OC2=CC(=CC(=C2C1=O)O)O)C3=CC(=CC(=C3)O)O |  | -7.142 | (Nessa et al., 2004) |  |
|  | mucic acid 6-ethyl ester 2-O-gallate |  |  |  | -7.061 | (Sun et al., 2023) |  |
|  | Higenamine/Norcoclaurine | 114840 | C1CNC(C2=CC(=C(C=C21)O)O)CC3=CC=C(C=C3)O |  | -7.045 | (Samanani & Facchini, 2001) |  |
|  | 1,5,6-Trihydroxy-3-methoxy xanthone |  |  |  | -7.029 | (Shen & Yang, 2006) |  |
|  | Roemerine | 119204 | CN1CCC2=CC3=C(C4=C2C1CC5=CC=CC=C54)OCO3 |  | -7.007 | (You et al., 1995) |  |
|  | alantolactone | 72724 | CC1CCCC2(C1=CC3C(C2)OC(=O)C3=C)C |  | -6.948 | (Tsuda et al., 1957) |  |
|  | (-)-Anonaine | 160597 | C1CNC2CC3=CC=CC=C3C4=C2C1=CC5=C4OCO5 |  | -6.944 | (T. Yang & Chen, 1970b) |  |
|  | Isobellidifolin | 5322042 | COC1=C2C(=C(C=C1)O)C(=O)C3=C(C=C(C=C3O2)O)O |  | -6.939 | (KOMATSU et al., 1969) |  |
|  | 1,3,8-Trihydroxy-5-methoxy xanthone | [5322042](https://pubchem.ncbi.nlm.nih.gov/compound/5322042) | COC1=C2C(=C(C=C1)O)C(=O)C3=C(C=C(C=C3O2)O)O |  | -6.936 | (Bennett et al., 1990) |  |
|  | (-)-Caaverine | 23335 | COC1=C(C2=C3C(CC4=CC=CC=C42)NCCC3=C1)O |  | -6.926 | (Ziyaev et al., 1973) |  |
|  | 1-O-galloyl-β-Dglucose | 124021 | C1=C(C=C(C(=C1O)O)O)C(=O)OC2C(C(C(C(O2)CO)O)O)O |  | -6.912 | (Abou-Zaid & Nozzolillo, 1999). |  |
|  | Dehydroemerine |  |  |  | -6.903 | (Chuliá et al., 2011) |  |
|  | Bellidifolin [ 1,5,8-trihydroxy-3- methoxyxanthone] | 5281623 | COC1=CC(=C2C(=C1)OC3=C(C=CC(=C3C2=O)O)O)O |  | -6.897 | (Basnet et al., 1994) |  |
|  | (-)-Asimilobine | 160875 | COC1=C(C=C2CCNC3C2=C1C4=CC=CC=C4C3)O |  | -6.795 | (Bick & Preston, 1971) |  |
|  | 1-Hydroxy-3,5-dimethoxy xanthone |  |  |  | -6.788 | (Locksley et al., 1971) |  |
|  | (-)-N-Methylasimilobine | 5319512 | CN1CCC2=CC(=C(C3=C2C1CC4=CC=CC=C43)OC)O |  | -6.780 | (F.-R. Chang et al., 1998) |  |
|  | Maclurin | 68213 | C1=CC(=C(C=C1C(=O)C2=C(C=C(C=C2O)O)O)O)O |  | -6.753 | (Healey, 1932) |  |
|  | N-methylasimilobine | 197017 | CN1CCC2=CC(=C(C3=C2C1CC4=CC=CC=C43)OC)O |  | -6.739 | (F.-R. Chang et al., 1998) |  |
|  | 2-chloro-bicyclooct-5-ene-2-carbonitrile | 138373833 | C1CCCC(CCC1)C2CCC=CCCC2(C#N)Cl |  | -6.734 | (Amir et al., 2014) |  |
|  | Grasshopper ketone | 10220146 | CC(=O)C=C=C1C(CC(CC1(C)O)O)(C)C |  | -6.722 | (Blomquist & Jackson, 1973) |  |
|  | Swertinin | 5491517 | COC1=C(C2=C(C=C1)OC3=CC(=CC(=C3C2=O)O)O)OC |  | -6.722 | (Kulanthaivel et al., 1988) |  |
|  | 4',6-Dimethoxy kaempferol | 5352001 | COC1=CC=C(C=C1)C2=C(C(=O)C3=C(C=C(C=C3O2)O)O)OC |  | -6.707 | (Fang & Mabry, 1986) |  |
|  | Iriflophenone | 11311158 | C1=CC(=CC=C1C(=O)C2=C(C=C(C=C2O)O)O)O |  | -6.693 | (Tay et al., 2014) |  |
|  | β-elemene | 9859094 | CC(=C)C1CCC(C(C1)C(=C)C)(C)C=C |  | -6.683 | (Z. Chen et al., 2009) |  |
|  | Swerchirin or Methylbellidifolin | 5281660 | COC1=C2C(=C(C=C1)O)C(=O)C3=C(C=C(C=C3O2)OC)O |  | -6.682 | (Markham, 1964) |  |
|  | Dehydroanonaine | 85821358 | C1CNC2=CC3=CC=CC=C3C4=C2C1=CC5=C4OCO5 |  | -6.647 | (Chuliá et al., 2011) |  |
|  | 2-Hydroxy-1-methoxy-6a,7-dehydroaporphine | 46203429 | CN1CCC2=CC(=C(C3=C2C1=CC4=CC=CC=C43)OC)O |  | -6.635 | (Nakamura et al., 2013) |  |
|  | 1-lycoperodine | 449440 | C1C(NCC2=C1C3=CC=CC=C3N2)C(=O)O |  | -6.620 | (Abat et al., 2017) |  |
|  | fisetin | 5281614 | C1=CC(=C(C=C1C2=C(C(=O)C3=C(O2)C=C(C=C3)O)O)O)O |  | -6.613 | (S.-H. Kim & Huh, 2022) |  |
|  | Lysicamine | 122691 | COC1=C(C2=C3C(=C1)C=CN=C3C(=O)C4=CC=CC=C42)OC |  | -6.594 | (Park et al., 1991) |  |
|  | 1,8-Dihydroxy-3,7-dimethoxy xanthone |  |  |  | -6.550 | (Asthana et al., 1991) |  |
|  | Swerimilegenin I |  |  |  | -6.538 | (Geng et al., 2013) |  |
|  | Liriodenine | 10144 | C1OC2=C(O1)C3=C4C(=C2)C=CN=C4C(=O)C5=CC=CC=C53 |  | -6.533 | (Hufford et al., 1975) |  |
|  | Decanal | 8175 | CCCCCCCCCC=O |  | -6.529 | (Ikeda et al., 1962) |  |
|  | (-)-Boscialin | 6442487 | CC1CC(CC(C1(C=CC(=O)C)O)(C)C)O |  | -6.501 | (Busch et al., 1998) |  |
|  | Desmethylbellidifolin | 5281626 | C1=CC(=C2C(=C1O)C(=O)C3=C(C=C(C=C3O2)O)O)O |  | -6.499 | (KOMATSU et al., 1969) |  |
|  | dl-salsoline | 46695 | CC1C2=CC(=C(C=C2CCN1)O)OC |  | -6.465 | (Achari et al., 1980) |  |
|  | Linalool | 6549 | CC(=CCCC(C)(C=C)O)C |  | -6.444 | (Foss & Harder, 1998) |  |
|  | Swertianin | 5281661 | COC1=CC(=C2C(=C1)OC3=C(C2=O)C(=C(C=C3)O)O)O |  | -6.415 | (KOMATSU et al., 1969) |  |
|  | Arbutin | 440936 | C1=CC(=CC=C1O)OC2C(C(C(C(O2)CO)O)O)O |  | -6.413 | (DURKEE et al., 1968) |  |
|  | Dihydrophaseic acid | 11988272 | CC(=CC(=O)O)C=CC1(C2(CC(CC1(OC2)C)O)C)O |  | -6.405 | (Martin et al., 1977) |  |
|  | 3-oxo-retro-α-ionol I |  |  |  | -6.394 | (Alarcón et al., 2019) |  |
|  | Norswertianin | 5281658 | C1=CC2=C(C(=C1O)O)C(=O)C3=C(C=C(C=C3O2)O)O |  | -6.313 | (KOMATSU et al., 1969) |  |
|  | (+)-Dehydrovomifoliol | 688492 | CC1=CC(=O)CC(C1(C=CC(=O)C)O)(C)C |  | -6.260 | (Y. Yang et al., 2013) |  |
|  | 3,3',5-trihydroxybiphenyl | 643386 | C1=CC(=CC(=C1)O)C2=C(C(=CC(=C2)O)O)C(=O)O |  | -6.198 | (KANAMORI et al., 1986) |  |
|  | Annuionone D | 14605579 | CC(=O)C=CC12C(CC(CC1(O2)C)O)(C)C |  | -6.179 | (Macías et al., 1999) |  |
|  | 5,6-epoxy-3-Hydroxy-7-megastigmen-9-one | 14605580 | CC(=O)C=CC12C(CC(CC1(O2)C)O)(C)C |  | -6.177 | (K. H. Kim et al., 2008) |  |
|  | 1,2,5,6-Tetrahydroxy xanthone |  |  |  | -6.167 | (Chanmahasathien et al., 2003) |  |
|  | Undecane | 14257 | CCCCCCCCCCC |  | -6.062 | (Cohen et al., 1992) |  |
|  | ellagic acid | 5281855 | C1=C2C3=C(C(=C1O)O)OC(=O)C4=CC(=C(C(=C43)OC2=O)O)O |  | -6.029 | (J.-H. Lee et al., 2005) |  |
|  | diethyl malate | 24197 | CCOC(=O)CC(C(=O)OCC)O |  | -6.008 | (KUO & CHANG, 2000) |  |
|  | 1,3,7-Trihydroxy-8-methoxy xanthone | 5322072 | COC1=C(C=CC2=C1C(=O)C3=C(C=C(C=C3O2)O)O)O |  | -5.947 | (U. R. Moon et al., 2021) |  |
|  | (-)-Roemerine | 235224 | CN1CCC2=CC3=C(C4=C2C1CC5=CC=CC=C54)OCO3 |  | -5.943 | (You et al., 1995) |  |
|  | Vomifoliol | 5280462 | CC1=CC(=O)CC(C1(C=CC(C)O)O)(C)C |  | -5.943 | (OKAMURA et al., 1981) |  |
|  | 2,5-dihydroxy terephthalic acid |  |  |  | -5.924 | (Hatjimanoli et al., 1988) |  |
|  | 1,3,7,8-Tetrahydroxy xanthone |  |  |  | -5.916 | (Jabit et al., 2007) |  |
|  | methyl eugenol | 7127 | COC1=C(C=C(C=C1)CC=C)OC |  | -5.890 | (Kaul et al., 2008) |  |
|  | 2- decenal | 5283345 | CCCCCCCC=CC=O |  | -5.890 | (Mattiacci et al., 1993) |  |
|  | 3-Hydroxymegastigm-7-en-9-one |  |  |  | -5.816 | (Weyerstahl et al., 1994) |  |
|  | Djalonenol | 15241181 | C=CC(CO)C1CCOC(=O)C1CO |  | -5.804 | (Onocha et al., 1995) |  |
|  | methyl caffeate | 689075 | COC(=O)C=CC1=CC(=C(C=C1)O)O |  | -5.786 | (INAYAMA et al., 1984) |  |
|  | 1,3-Dihydroxy xanthone |  |  |  | -5.775 | (IINUMA et al., 1996) |  |
|  | methyl gallate | 7428 | COC(=O)C1=CC(=C(C(=C1)O)O)O |  | -5.721 | (Bailey et al., 1986) |  |
|  | Oleracein E | 21574476 | C1CC(=O)N2C1C3=CC(=C(C=C3CC2)O)O |  | -5.660 | (Xiang et al., 2005) |  |
|  | Nonacosanyl hentriacontanoate |  |  |  | -5.654 | (Neerja et al., 2022) |  |
|  | nerol | 643820 | CC(=CCCC(=CCO)C)C |  | -5.653 | (Cori et al., 1986) |  |
|  | β-bourbonene | 324224 | CC(C)C1CCC2(C1C3C2CCC3=C)C |  | -5.647 | (Tomioka et al., 1982) |  |
|  | Chrysoeriol | 5280666 | COC1=C(C=CC(=C1)C2=CC(=O)C3=C(C=C(C=C3O2)O)O)O |  | -5.629 | (Tai et al., 2009) |  |
|  | Ethyl gallate | 13250 | CCOC(=O)C1=CC(=C(C(=C1)O)O)O |  | -5.622 | (Baderschneider & Winterhalter, 2001) |  |
|  | Syringaldehyde | 8655 | COC1=CC(=CC(=C1O)OC)C=O |  | -5.610 | (Pedroso et al., 2008) |  |
|  | (E)-Ferulic acid | 445858 | COC1=C(C=CC(=C1)C=CC(=O)O)O |  | -5.608 | (Garcia-Conesa et al., 1997) |  |
|  | Mallotusinin | 514180 | C1C2C3C(C(C(O2)OC(=O)C4=CC(=C(C(=C4)O)O)O)OC(=O)C5=CC(=C(C6=C5C7=C(O6)C(=C(C=C7C(=O)O3)O)O)O)O)OC(=O)C8=CC(=C(C(=C8C9=C(C(=C(C=C9C(=O)O1)O)O)O)O)O)O |  | -5.601 | (SAIJO et al., 1989) |  |
|  | Vanillic acid | 8468 | COC1=C(C=CC(=C1)C(=O)O)O |  | -5.585 | (Huang et al., 1993) |  |
|  | coniferyl aldehyde / 4-Hydroxy-3-methoxycinnam aldehyde | 5280536 | COC1=C(C=CC(=C1)C=CC=O)O |  | -5.583 | (Brandt et al., 2001) |  |
|  | Sinapaldehyde | 5280802 | COC1=CC(=CC(=C1O)OC)C=CC=O |  | -5.581 | (Black et al., 1953) |  |
|  | Erythrocentaurin | 191120 | C1COC(=O)C2=CC=CC(=C21)C=O |  | -5.576 | (Wenkert et al., 1964) |  |
|  | 2-hydroxy terephthalic acid | 97257 | C1=CC(=C(C=C1C(=O)O)O)C(=O)O |  | -5.557 | (Huskić & Žigon, 2003) |  |
|  | (E)-p-Coumaric acid | 637542 | C1=CC(=CC=C1C=CC(=O)O)O |  | -5.554 | (Karthikeyan et al., 2015) |  |
|  | cumin aldehyde | 326 | CC(C)C1=CC=C(C=C1)C=O |  | -5.547 | (H.-S. Lee, 2005) |  |
|  | 3-Indoleacetic acid | 802 | C1=CC=C2C(=C1)C(=CN2)CC(=O)O |  | -5.539 | (Hamilton et al., 1961) |  |
|  | Enicoflavine | 5281564 | C=CC(C=O)C1(CCOC(=O)C1=CN)O |  | -5.485 | (Ghosal et al., 1974) |  |
|  | Decane | 15600 | CCCCCCCCCC |  | -5.466 | (IIZUKA et al., 1966) |  |
|  | Galactaric acid | 3037582 | C(C(C(C(=O)O)O)O)(C(C(=O)O)O)O |  | -5.451 | (Jeffrey & Wood, 1982) |  |
|  | myrcene | 31253 | CC(=CCCC(=C)C=C)C |  | -5.428 | (Bohlmann et al., 1997) |  |
|  | 1,2Benzenedicarboxylic acid | 1017 | C1=CC=C(C(=C1)C(=O)O)C(=O)O |  | -5.428 | (Das et al., 2012) |  |
|  | (+)-Epiloliolide | 44511808 | CC1(CC(CC2(C1=CC(=O)O2)C)O)C |  | -5.405 | (Mori & Khlebnikov, 1993) |  |
|  | (3-ethoxy-4,5-dihydroxy-benzoic acid), isostrictiniin | 86047602 | CCOC1=CC(=CC(=C1O)O)C(=O)O |  | -5.403 | (L. Zhang et al., 2003) |  |
|  | Erythrocentauric acid | 118726453 | C1COC(=O)C2=C1C(=CC=C2)C(=O)O |  | -5.377 | (CHEN et al., 1994) |  |
|  | Gentianine | 354616 | C=CC1=CN=CC2=C1CCOC2=O |  | -5.367 | (Wolfender et al., 2015) |  |
|  | Scopoletin | 5280460 | COC1=C(C=C2C(=C1)C=CC(=O)O2)O |  | -5.362 | (Goodwin & Kavanagh, 1949) |  |
|  | Syringic acid | 10742 | COC1=CC(=CC(=C1O)OC)C(=O)O |  | -5.289 | (Dar & Ikram, 1979) |  |
|  | 4-hydroxy-3-methoxy benzaldehyde | 1183 | COC1=C(C=CC(=C1)C=O)O |  | -5.232 | (L. Wang et al., 2014) |  |
|  | 1H-Imidazole, 1-(phenylmethyl) |  |  |  | -5.193 | (Triolo et al., 1991) |  |
|  | Ethyl benzoate | 7165 | CCOC(=O)C1=CC=CC=C1 |  | -5.186 | (Gettler et al., 1932) |  |
|  | Gallic acid | 370 | C1=C(C=C(C(=C1O)O)O)C(=O)O |  | -5.142 | (Kambourakis et al., 2000) |  |
|  | Anisic acid | 7478 | COC1=CC=C(C=C1)C(=O)O |  | -5.136 | (Fausto et al., 1997) |  |
|  | methyl salicylate | 4133 | COC(=O)C1=CC=CC=C1O |  | -5.109 | (J.-H. Moon et al., 1996) |  |
|  | 1H-Indole, 1,2-dimethyl | 13408 | CC1=CC2=CC=CC=C2N1C |  | -5.107 | (Helen et al., 2012) |  |
|  | Cinnamic acid | 444539 | C1=CC=C(C=C1)C=CC(=O)O |  | -5.071 | (R. A, 1994) |  |
|  | elaeocarpusin | 492393 | C1C(C2C(O1)(C3(C(=O)O2)C45CC(=O)C(O3)(C6(C4C7=C(O6)C(=C(C=C7C(=O)OC8C9C(C(COC(=O)C1=CC(=C(C(=C1C1=C(C(=C(C=C1C(=O)O9)O)O)O)O)O)O)OC8OC(=O)C1=CC(=C(C(=C1)O)O)O)OC5=O)O)O)O)O)O)O |  | -5.065 | (Tanaka et al., 1986) |  |
|  | Sarpagine | 12314884 | CC=C1CN2C3CC1C(C2CC4=C3NC5=C4C=C(C=C5)O)CO |  | -5.028 | (Lounasmaa et al., 1999) |  |
|  | Protocatechuic acid | 72 | C1=CC(=C(C=C1C(=O)O)O)O |  | -5.019 | (Link et al., 1929) |  |
|  | limonene | 22311 | CC1=CCC(CC1)C(=C)C |  | -4.999 | (R. Kodama et al., 1976) |  |
|  | m-hydroxybenzoic acid | 7420 | C1=CC(=CC(=C1)O)C(=O)O |  | -4.986 | (Vieitez et al., 1966) |  |
|  | Indolizine, 2,6-dimethyl | 589391 | CC1=CN2C=C(C=C2C=C1)C |  | -4.961 | (Prostakov & Baktibaev, 1972) |  |
|  | p-Hydroxybenzoic acid | 135 | C1=CC(=CC=C1C(=O)O)O |  | -4.958 | (Vieitez et al., 1966) |  |
|  | 2-Methylbutyl acetate | 12209 | CCC(C)COC(=O)C |  | -4.915 | (Bail et al., 2009) |  |
|  | pyrimidine 2,4,5-Triamine | 148506 | C1=C(C(=NC(=N1)N)N)N |  | -4.896 | (Gillespie et al., 2009) |  |
|  | acetophenone | 7410 | CC(=O)C1=CC=CC=C1 |  | -4.848 | (Ohmori et al., 1988) |  |
|  | Propane phosphonicacid |  |  |  | -4.782 | (Wackett et al., 1987) |  |
|  | Pyrocatechol | 289 | C1=CC=C(C(=C1)O)O |  | -4.629 | (Yoshida et al., 1982) |  |
|  | Gentiocrucine |  |  |  | -4.607 | (Ganem, 1976) |  |
|  | Tartaric acid | 875 | C(C(C(=O)O)O)(C(=O)O)O |  | -4.513 | (Kotera et al., 1972) |  |
|  | 5-hydroxymethylfurfural | 237332 | C1=C(OC(=C1)C=O)CO |  | -4.47823 | (ULBRICHT et al., 1984) |  |
|  | β-caryophyllene | 5281522 | CC1=CCCC(=C)C2CC(C2CC1)(C)C |  | -4.429 | (Dahham et al., 2015) |  |
|  | Benzaldehyde | 240 | C1=CC=C(C=C1)C=O |  | -4.407 | (Sneh & Cheshnovsky, 1991) |  |
|  | coahuilensol | 15605901 | CC1=C(C(=CC=C1)O)C=C |  | -4.395 | (Adams et al., 2007) |  |
|  | α-Furfural | 7362 | C1=COC(=C1)C=O |  | -4.097 | (Al-Syari & Hassan, 2023) |  |
|  | Quercetin-3-O-β-D-glucopyranoside | 12304324 | C1=CC(=C(C=C1C2=C(C(=O)C3=C(C=C(C=C3O2)O)O)OC4C(C(C(C(O4)CO)O)O)O)O)O |  | -2.926 | (T. Kodama et al., 1990) |  |
|  | choline | 305 | C[N+](C)(C)CCO |  | -2.016 | (Fonnum, 1969) |  |
|  | 4,5,7'-Trihydroxy-4' methoxy flavone-7-O-β-D glucopyranoside | 53317532 | COC1=C(OC2=CC(=CC(=C2C1=O)O)OC3C(C(C(C(O3)CO)O)O)O)C4=CC=C(C=C4)O |  | -0.520 | (Abu-Niaaj & Katampe, 2018) |  |
|  | Apigenin-7-O-β-glupyranoside | 12304094 | C1=CC(=CC=C1C2=CC(=O)C3=C(C=C(C=C3O2)OC4C(C(C(C(O4)CO)O)O)O)O)O |  | 1.170 | (R et al., 2003) |  |
|  | Octacosene-1 | 87821 | CCCCCCCCCCCCCCCCCCCCCCCCCCC=C |  | 1.275 | (Fonnum, 1969) |  |
|  | Tetrapentacontane | 521846 | CCCCCCCCCCCCCCCCCCCCCCCCCCCCCCCCCCCCCCCCCCCCCCCCCCCCCC |  | 2.645 | (Ali et al., 2021) |  |
|  | Chrysoeriol-7-O-β-glupyranoside | 13871880 | COC1=C(C=CC(=C1)C2=CC(=O)C3=C(C=C(C=C3O2)OC4C(C(C(C(O4)CO)O)O)O)O)O |  | 3.178 | (Gomaa et al., 2018) |  |
|  | Gossypetin 8-O-β glucoside | 5281621 | C1=CC(=C(C=C1C2=C(C(=O)C3=C(O2)C(=C(C=C3O)O)OC4C(C(C(C(O4)CO)O)O)O)O)O)O |  | 3.583 | (Thuong et al., 2007) |  |
|  | Squalene | 638072 | CC(=CCCC(=CCCC(=CCCC=C(C)CCC=C(C)CCC=C(C)C)C)C)C |  | 4.049 | (He et al., 2002) |  |
|  | Quercetin-3-O-α-rhamnopyranosyl (1-6)-β-glucopyranoside / Rutin | 5280805 | CC1C(C(C(C(O1)OCC2C(C(C(C(O2)OC3=C(OC4=CC(=CC(=C4C3=O)O)O)C5=CC(=C(C=C5)O)O)O)O)O)O)O)O |  | 4.096 | (Buszewski et al., 1993) |  |
|  | mucic acid lactone gallate | 129641771 | C1=C(C=C(C(=C1O)O)O)C(=O)C2(C(C(C(C(O2)(C(=O)C3=CC(=C(C(=C3)O)O)O)OC(=O)C4=CC(=C(C(=C4)O)O)O)(C(=O)C5=CC(=C(C(=C5)O)O)O)OC(=O)C6=CC(=C(C(=C6)O)O)O)(C(=O)C7=CC(=C(C(=C7)O)O)O)OC(=O)C8=CC(=C(C(=C8)O)O)O)(C(=O)C9=CC(=C(C(=C9)O)O)O)O)COC(=O)C1=CC(=C(C(=C1)O)O)O |  | 5.274 | (Majeed et al., 2009) |  |
|  | Cyanidin-3-O-rutinoside | 441674 | CC1C(C(C(C(O1)OCC2C(C(C(C(O2)OC3=CC4=C(C=C(C=C4[O+]=C3C5=CC(=C(C=C5)O)O)O)O)O)O)O)O)O)O |  | 7.332 | (P. Liu et al., 2020) |  |
|  | trans-N-Coumaroyltyramine |  |  |  | --8.506 | (Al-Taweel et al., 2012) |  |
|  | 20, 23-Dimethylcholesta-6, 22-dien-3β-ol |  |  |  | 9.676 | (Gomaa et al., 2018) |  |
|  | Methyl triacontanoate | 12400 | CCCCCCCCCCCCCCCCCCCCCCCCCCCCCC(=O)OC |  | 13.028 | (Rao & Supharao, 1992) |  |
|  | Cholest-5-en-3-beta- ol, 4, 4-dimethyl- | 280427 | CC(C)CCCC(C)C1CCC2C1(CCC3C2CC=C4C3(CCC(C4(C)C)O)C)C |  | 15.065 | (Dikshit, 2017) |  |
|  | 9,19-Cyclolanost-3-one-24,25-diol | 129847807 | CC(CCC(C(C)(C)O)O)C1CCC2(C1(CCC34C2CCC5C3(C4)CCC(=O)C5(C)C)C)C |  | 16.909 | (Barik et al., 1994) |  |
|  | (24R)-5α Stigmastane-3,6 dione | 13992092 | CCC(CCC(C)C1CCC2C1(CCC3C2CC(=O)C4C3(CCC(=O)C4)C)C)C(C)C |  | 17.609 | (C.-C. Zhao et al., 2005) |  |
|  | β-Sitostrol-3-O-β-D-glucopyranoside | 12309057 | CCC(CCC(C)C1CCC2C1(CCC3C2CC=C4C3(CCC(C4)OC5C(C(C(C(O5)CO)O)O)O)C)C)C(C)C |  | 21.893 | (Gomaa et al., 2018) |  |
|  | Lup-20(29)-en-3.beta.-ol, acetate | 12900948 | CC(=C)C1CCC2(C1C3CCC4C5(CCC(C(C5CCC4(C3(CC2)C)C)(C)C)OC(=O)C)C)C |  | 29.038 | (Karau et al., 2014) |  |
|  | β-Amyrin-3-palmitate | 146159295 | CCCCCCCCCCCCCCCC(=O)OC1CCC2(C(C1(C)C)CCC3(C2CC=C4C3(CCC5(C4CC(CC5)(C)C)C)C)C)C |  | 90.238 | (H. S. Lee et al., 2000) |  |
