# Supplementary material for: Computational analysis of Ayurvedic metabolites for potential treatment of drug-resistant Candida auris
Source: Front Cell Infect Microbiol. 2025 Mar 13;15:1537872. doi: 10.3389/fcimb.2025.1537872 (PMC11979702; doi:10.3389/fcimb.2025.1537872)
Supplement: Supplementary file 2 [file Table2.docx]

**Table S2.** Physicochemical Analysis of top selected metabolites by SwissADME webserver.

| **Sr. No.** | **Metabolites** | **PHYSICOCHEMICAL PROPERTIES** | | | | | | | | | |
| --- | --- | --- | --- | --- | --- | --- | --- | --- | --- | --- | --- |
|  |  | **Formula** | **Molecular weight** | **Num. heavy atoms** | **Num. arom. heavy atoms** | **Fraction Csp3** | **Num. rotatable bonds** | **Num. H-bond acceptors** | **Num. H-bond donors** | **Molar Refractivity** | **TPSA** |
|  | 4-Hydroxybenzoate | C7H5O3- | 137.11 g/mol | 10 | 6 | 0.00 | 1 | 3 | 1 | 33.53 | 60.36 Å² |
|  | Methylcoumarate | C10H10O3 | 178.18 g/mol | 13 | 6 | 0.10 | 3 | 3 | 1 | 49.46 | 46.53 Å² |
|  | 2,6-Dihydroxy-4-methoxyacetophenone | C9H10O4 | 182.17 g/mol | 13 | 6 | 0.22 | 2 | 4 | 2 | 47.17 | 66.76 Å² |
|  | Trans-p-coumaric acid | C9H8O3 | 164.16 g/mol | 12 | 6 | 0.00 | 2 | 3 | 2 | 45.13 | 57.53 Å² |
|  | Isoliensinine | C37H42N2O6 | 610.74 g/mol | 45 | 24 | 0.35 | 9 | 8 | 2 | 183.55 | 83.86 Å² |
|  | Neferine | C38H44N2O6 | 624.77 g/mol | 46 | 24 | 0.37 | 10 | 8 | 1 | 188.02 | 72.86 Å² |
|  | Eudesmic acid | C10H12O5 | 212.20 g/mol | 15 | 6 | 0.30 | 4 | 5 | 1 | 52.88 | 64.99 Å² |
|  | Liensinine | C37H42N2O6 | 610.74 g/mol | 45 | 24 | 0.35 | 9 | 8 | 2 | 183.55 | 83.86 Å² |
|  | Scoparone | C11H10O4 | 206.19 g/mol | 15 | 10 | 0.18 | 2 | 4 | 0 | 55.47 | 48.67 Å² |
|  | (R)-N-(1’-methoxycarbonyl-2’-phenylethyl)-4-hydroxybenzamide | C17H17NO4 | 299.32 g/mol | 22 | 12 | 0.18 | 7 | 4 | 2 | 81.65 | 75.63 Å² |
