# Supplementary material for: Computational analysis of Ayurvedic metabolites for potential treatment of drug-resistant Candida auris
Source: Front Cell Infect Microbiol. 2025 Mar 13;15:1537872. doi: 10.3389/fcimb.2025.1537872 (PMC11979702; doi:10.3389/fcimb.2025.1537872)
Supplement: Supplementary file 3 [file Table3.docx]

**Table S3.** Per-residue energy decomposition of trans-p-coumaric acid.

| **Total Energy Decomposition:** trans-p-coumaric acid | | | | | |
| --- | --- | --- | --- | --- | --- |
| **Residue** | **van der Waals** | **Electrostatic** | **Polar Solvation** | **Non-Polar Solv.** | **TOTAL** |
| PHE 81 | -0.571 | -0.0844 | 0.2089 | -0.0916596 | -0.5381596 |
| VAL 86 | -1.039 | -0.0358 | -0.0431 | -0.17685216 | -1.29475216 |
| LEU 94 | -0.1747 | -0.1828 | 0.135 | -0.0158328 | -0.2383328 |
| GLN 97 | -0.0371 | -2.1101 | 1.0228 | -0.02635632 | -1.15075632 |
| LYS 98 | -0.6121 | 1.0125 | -0.938 | -0.06320448 | -0.60080448 |
| PHE 100 | -0.161 | 0.1264 | -0.2647 | -0.00296352 | -0.30226352 |
| VAL 101 | -0.6516 | 0.1358 | -0.2161 | -0.0763488 | -0.8082488 |
| MET 251 | -0.7104 | -0.2903 | 0.2013 | -0.05686632 | -0.85626632 |
| ALA 254 | -0.8325 | 0.2014 | 0.2975 | -0.05859288 | -0.39219288 |
| LEU 255 | -0.5955 | -0.0206 | -0.2505 | -0.02546712 | -0.89206712 |
| MET 257 | -0.2705 | -0.1833 | -0.0398 | -0.01575144 | -0.50935144 |
| ALA 258 | -0.5881 | -0.0618 | -0.0171 | -0.08762472 | -0.75462472 |
| CYS 414 | -0.6281 | -0.3999 | 0.2702 | -0.12739824 | -0.88519824 |
| ILE 415 | -0.96 | -0.2906 | 0.2146 | -0.1272744 | -1.1632744 |
