# Supplementary material for: Computational analysis of Ayurvedic metabolites for potential treatment of drug-resistant Candida auris
Source: Front Cell Infect Microbiol. 2025 Mar 13;15:1537872. doi: 10.3389/fcimb.2025.1537872 (PMC11979702; doi:10.3389/fcimb.2025.1537872)
Supplement: Supplementary file 4 [file Table4.docx]

**Table S4.** Per-residue energy decomposition of MCPHB [(r)-n-(1'-methoxycarbonyl-2'-phenylethyl)-4-hydroxybenzamide].

| **Total Energy Decomposition: MCPHB** | | | | | |
| --- | --- | --- | --- | --- | --- |
| **Residue** | **van der Waals** | **Electrostatic** | **Polar Solvation** | **Non-Polar Solv.** | **TOTAL** |
| ALA 69 | 0.6029 | -4.2187 | 2.0155 | -0.03127 | -1.63157 |
| GLU 70 | -0.45 | -1.5005 | 1.402 | -0.02567 | -0.57417 |
| TYR 73 | -0.7296 | 0.1215 | 0.1682 | -0.0548 | -0.4947 |
| PHE 81 | -0.5877 | -0.3393 | 0.0966 | -0.0546 | -0.885 |
| VAL 86 | -1.3957 | -0.1367 | 0.4483 | -0.18119 | -1.26529 |
| TYR 87 | -2.1587 | -3.4131 | 3.775 | -0.36253 | -2.15933 |
| LYS 98 | -0.2922 | -9.6185 | 10.0488 | -0.10089 | 0.037211 |
| ALA 254 | -0.4023 | -0.1813 | 0.5155 | -0.04108 | -0.10918 |
| ALA 258 | -0.665 | 0.1673 | 0.1389 | -0.18083 | -0.53963 |
| HIS 261 | -0.2592 | 0.0521 | 0.1965 | -0.03917 | -0.04977 |
| ILE 324 | -0.6307 | -0.0007 | 0.0562 | -0.21685 | -0.79205 |
| ARG 329 | -0.3646 | -1.5696 | 0.72 | -0.02499 | -1.23919 |
| HIS 412 | -2.7326 | -1.0059 | 2.1551 | -0.29655 | -1.87995 |
| ARG 413 | -0.9431 | 0.0268 | 0.7673 | -0.0233 | -0.1723 |
| CYS 414 | -1.5398 | -0.608 | 1.0219 | -0.23018 | -1.35608 |
| ILE 415 | -0.9176 | -0.028 | 0.4314 | -0.10437 | -0.61857 |
