# Supplementary material for: Computational analysis of Ayurvedic metabolites for potential treatment of drug-resistant Candida auris
Source: Front Cell Infect Microbiol. 2025 Mar 13;15:1537872. doi: 10.3389/fcimb.2025.1537872 (PMC11979702; doi:10.3389/fcimb.2025.1537872)
Supplement: Supplementary file 5 [file Table5.docx]

**Table S5.** Per-residue energy decomposition of VNI.

| **Total Energy Decomposition: VNI** | | | | | |
| --- | --- | --- | --- | --- | --- |
| **Residue** | **van der Waals** | **Electrostatic** | **Polar Solvation** | **Non-Polar Solv.** | **TOTAL** |
| LEU 42 | -0.5007 | 0.0194 | 0.1005 | -0.11351376 | -0.49431376 |
| LEU 43 | -0.3178 | -0.0208 | 0.1246 | -0.06313824 | -0.27713824 |
| LYS 45 | -0.0625 | 0.5841 | -0.4363 | -0.00477144 | 0.08052856 |
| VAL 72 | -0.9012 | 0.1821 | -0.4875 | -0.10530072 | -1.31190072 |
| TYR 73 | -1.6703 | -0.8104 | 0.7132 | -0.10799136 | -1.87549136 |
| LEU 76 | -1.4969 | -0.1473 | 0.0528 | -0.13440168 | -1.72580168 |
| THR 77 | -1.35 | -0.1576 | 0.3904 | -0.122148 | -1.239348 |
| PHE 81 | -1.1997 | 0.0054 | 0.0926 | -0.13663008 | -1.23833008 |
| VAL 86 | -0.5658 | -0.0184 | 0.0129 | -0.10760832 | -0.67890832 |
| TYR 87 | -1.3675 | -0.7544 | 1.3697 | -0.22161456 | -0.97381456 |
| PHE 180 | -1.0836 | 0.128 | 0.4105 | -0.11264832 | -0.65774832 |
| PHE 185 | -1.8906 | -0.2934 | 0.7247 | -0.27236376 | -1.73166376 |
| ALA 254 | -0.6127 | -0.2863 | 0.2308 | -0.0272772 | -0.6954772 |
| MET 257 | -0.9275 | 0.2171 | 0.059 | -0.04228056 | -0.69368056 |
| ALA 258 | -1.5539 | 0.3539 | -0.2654 | -0.14510952 | -1.61050952 |
| HIE 261 | -1.477 | -0.3022 | 0.8133 | -0.13005216 | -1.09595216 |
| SER 262 | -0.5055 | -0.7097 | 0.4068 | -0.02651112 | -0.83491112 |
| ILE 324 | -0.9789 | 0.1769 | -0.1309 | -0.1887156 | -1.1216156 |
| ILE 328 | -1.2932 | -0.11 | 0.0706 | -0.1591308 | -1.4917308 |
| ASN 349 | -0.1803 | 0.2471 | -0.0308 | -0.0730188 | -0.0370188 |
| CYS 414 | -0.8 | -1.0485 | 0.5907 | -0.14918616 | -1.40698616 |
| ILE 415 | -0.6565 | -0.0614 | 0.1461 | -0.08232696 | -0.65412696 |
| SER 453 | -0.6885 | -0.4017 | 0.3818 | -0.07017192 | -0.77857192 |
| LEU 454 | -2.3618 | 0.1343 | 0.1546 | -0.27689184 | -2.34979184 |
| PHE 455 | -1.4903 | -0.1913 | 0.2239 | -0.14936688 | -1.60706688 |
