# Supplementary material for: Computational analysis of Ayurvedic metabolites for potential treatment of drug-resistant Candida auris
Source: Front Cell Infect Microbiol. 2025 Mar 13;15:1537872. doi: 10.3389/fcimb.2025.1537872 (PMC11979702; doi:10.3389/fcimb.2025.1537872)
Supplement: Supplementary file 6 [file Table6.docx]

**Table S6.** Bioavailability Score Analysis of top selected metabolites by SwissADME webserver.

| **Sr. No.** | **Compounds** | **XLOGP3** | **Molecular size** | **Polarity** | **Solubility** | **Saturation** | **Flexibility** |
| --- | --- | --- | --- | --- | --- | --- | --- |
| 1 | 4-Hydroxybenzoate | 1.58 | 137.11 g/mol | 60.36 Å² | -2.06 | 0.00 | 1 |
| 2 | Methylcoumarate | 2.26 | 178.18 g/mol | 46.53 Å² | -2.51 | 0.10 | 3 |
| 3 | 2,6-Dihydroxy-4-methoxyacetophenone | 1.42 | 182.17 g/mol | 66.76 Å² | -2.07 | 0.22 | 2 |
| 4 | trans-p-coumaric acid | 1.46 | 164.16 g/mol | 57.53 Å² | -2.02 | 0.00 | 2 |
| 5 | Isoliensinine | 6.38 | 610.74 g/mol | 83.86 Å² | -7.45 | 0.35 | 9 |
| 6 | Neferine | 6.70 | 624.77 g/mol | 72.86 Å² | -7.66 | 0.37 | 10 |
| 7 | Eudesmic acid | 1.45 | 212.20 g/mol | 64.99 Å² | -2.10 | 0.30 | 4 |
| 8 | Liensinine | 6.38 | 610.74 g/mol | 83.86 Å² | -7.45 | 0.35 | 9 |
| 9 | Scoparone | 1.71 | 206.19 g/mol | 48.67 Å² | -2.56 | 0.18 | 2 |
| 10 | (R)-N-(1’-methoxycarbonyl-2’-phenylethyl)-4-hydroxybenzamide | 2.22 | 299.32 g/mol | 75.63 Å² | -3.04 | 0.18 | 7 |
