# Supplementary material for: Computational analysis of Ayurvedic metabolites for potential treatment of drug-resistant Candida auris
Source: Front Cell Infect Microbiol. 2025 Mar 13;15:1537872. doi: 10.3389/fcimb.2025.1537872 (PMC11979702; doi:10.3389/fcimb.2025.1537872)
Supplement: Supplementary file 7 [file Table7.docx]

**Table S7.** Medicinal chemistry Analysis of top selected metabolites by SwissADME webserver.

| **Sr. No.** | **Metabolites** | **MEDICINAL CHEMISTRY** | | | |
| --- | --- | --- | --- | --- | --- |
|  |  | **PAINS Alerts** | **Brenk Alerts** | **Lead-likeness** | **Synthetic accessibility** |
| 1 | 4-Hydroxybenzoate | 0 | 0 | No;  MW<250 | 1.00 |
| 2 | Methylcoumarate | 0 | 1 | No;  MW<250 | 1.84 |
| 3 | 2,6-Dihydroxy-4-Methoxyacetophenone | 0 | 0 | No;  MW<250 | 1.31 |
| 4 | Trans-p-coumaric acid | 0 | 1 | No;  MW<250 | 1.61 |
| 5 | Isoliensinine | 0 | 0 | No; MW>350, Rotors>7, XLOGP3>3.5 | 5.09 |
| 6 | Neferine | 0 | 0 | No; MW>350, Rotors>7, XLOGP3>3.5 | 5.22 |
| 7 | Eudesmic acid | 0 | 0 | No;  MW<250 | 1.83 |
| 8 | Liensinine | 0 | 0 | No; MW>350, Rotors>7, XLOGP3>3.5 | 5.09 |
| 9 | Scoparone | 0 | 1 | No;  MW<250 | 2.77 |
| 10 | (R)-N-(1’-methoxycarbonyl-2’-phenylethyl)-4-hydroxybenzamide | 0 | 0 | Yes | 2.37 |
