# Supplementary material for: Computational analysis of Ayurvedic metabolites for potential treatment of drug-resistant Candida auris
Source: Front Cell Infect Microbiol. 2025 Mar 13;15:1537872. doi: 10.3389/fcimb.2025.1537872 (PMC11979702; doi:10.3389/fcimb.2025.1537872)
Supplement: Supplementary file 8 [file Table8.docx]

**Table S8.** ADMET Analysis of top selected metabolites by ADMETlab 2.0.

|  | **4-Hydroxybenzoate** | **Methylcoumarate** | **2,6-Dihydroxy-4-methoxyacetophenone** | **Trans-p-coumaric acid** | **Isoliensinine** | **Neferine** | **Eudesmic acid** | **Liensinine** | **Scoparone** | **(R)-N-(1’-methoxycarbonyl-2’-phenylethyl)-4-hydroxybenzamide**  **(MCPHB)** |
| --- | --- | --- | --- | --- | --- | --- | --- | --- | --- | --- |
| **Absorption** | | | | | | | | | | |
| **Caco-2 Permeability** | -4.936 | -4.527 | -4.707 | -4.768 | -5.601 | -5.654 | -4.843 | -5.598 | -4.656 | -4.632 |
| **MDCK Permeability** | 1.6e-05 | 1.7e-05 | 1.1e-05 | 7.3e-06 | 1.4e-05 | 2.2e-05 | 1.3e-05 | 1.6e-05 | 3.1e-05 | 1.6e-05 |
| **Pgp-inhibitor** | 0.001 | 0.0 | 0.001 | 0.0 | 0.999 | 1.0 | 0.002 | 0.997 | 0.019 | 0.001 |
| **Pgp-substrate** | 0.0 | 0.225 | 0.003 | 0.012 | 0.012 | 0.016 | 0.001 | 0.011 | 0.128 | 0.005 |
| **HIA** | 0.005 | 0.008 | 0.011 | 0.011 | 0.005 | 0.004 | 0.008 | 0.005 | 0.005 | 0.016 |
| **F20%** | 0.002 | 0.003 | 0.039 | 0.17 | 0.891 | 0.877 | 0.007 | 0.9 | 0.008 | 0.019 |
| **F30%** | 0.005 | 0.96 | 0.273 | 0.979 | 0.961 | 0.955 | 0.015 | 0.968 | 0.988 | 0.641 |
| **Distribution** | | | | | | | | | | |
| **PPB** | 69.502% | 62.054% | 91.372% | 67.862% | 92.142% | 88.403% | 31.820% | 88.689% | 66.483% | 61.248% |
| **VD** | 0.207 | 0.338 | 0.836 | 0.260 | 1.310 | 1.364 | 0.392 | 1.345 | 0.882 | 0.556 |
| **BBB Penetration** | 0.527 | 0.19 | 0.104 | 0.085 | 0.257 | 0.411 | 0.756 | 0.174 | 0.498 | 0.268 |
| **Fu** | 21.457% | 19.255% | 10.820% | 17.822% | 6.955% | 6.356% | 45.980% | 7.347% | 25.660% | 30.685% |
| **Metabolism** | | | | | | | | | | |
| **CYP1A2 inhibitor** | 0.038 | 0.982 | 0.958 | 0.087 | 0.089 | 0.047 | 0.04 | 0.057 | 0.967 | 0.709 |
| **CYP1A2 substrate** | 0.054 | 0.21 | 0.905 | 0.055 | 0.978 | 0.981 | 0.941 | 0.97 | 0.968 | 0.117 |
| **CYP2C19inhibitor** | 0.042 | 0.554 | 0.22 | 0.038 | 0.066 | 0.062 | 0.03 | 0.059 | 0.319 | 0.717 |
| **CYP2C19substrate** | 0.05 | 0.08 | 0.115 | 0.047 | 0.963 | 0.981 | 0.082 | 0.964 | 0.624 | 0.066 |
| **CYP2C9 inhibitor** | 0.018 | 0.239 | 0.296 | 0.08 | 0.02 | 0.015 | 0.017 | 0.017 | 0.034 | 0.733 |
| **CYP2C9 substrate** | 0.168 | 0.961 | 0.892 | 0.837 | 0.62 | 0.624 | 0.242 | 0.614 | 0.855 | 0.837 |
| **CYP2D6 inhibitor** | 0.008 | 0.327 | 0.569 | 0.065 | 0.028 | 0.027 | 0.009 | 0.032 | 0.178 | 0.102 |
| **CYP2D6 substrate** | 0.079 | 0.733 | 0.667 | 0.223 | 0.971 | 0.974 | 0.153 | 0.967 | 0.906 | 0.574 |
| **CYP3A4 inhibitor** | 0.024 | 0.657 | 0.535 | 0.09 | 0.069 | 0.102 | 0.011 | 0.057 | 0.085 | 0.436 |
| **CYP3A4 substrate** | 0.063 | 0.226 | 0.187 | 0.044 | 0.951 | 0.957 | 0.114 | 0.952 | 0.475 | 0.288 |
| **Excretion** | | | | | | | | | | |
| **CL** | 2.970 | 11.476 | 11.465 | 5.919 | 12.065 | 10.671 | 6.750 | 12.244 | 11.015 | 7.163 |
| **T1/2** | 0.921 | 0.889 | 0.777 | 0.898 | 0.521 | 0.432 | 0.932 | 0.712 | 0.814 | 0.830 |
| **Toxicity** | | | | | | | | | | |
| **hERG Blockers** | 0.066 | 0.031 | 0.041 | 0.01 | 0.967 | 0.983 | 0.058 | 0.948 | 0.203 | 0.164 |
| **H-HT** | 0.874 | 0.162 | 0.046 | 0.727 | 0.095 | 0.094 | 0.213 | 0.078 | 0.368 | 0.287 |
| **DILI** | 0.872 | 0.302 | 0.788 | 0.806 | 0.302 | 0.683 | 0.856 | 0.286 | 0.873 | 0.744 |
| **AMES Toxicity** | 0.048 | 0.258 | 0.315 | 0.036 | 0.097 | 0.082 | 0.009 | 0.082 | 0.117 | 0.036 |
| **Rat Oral Acute Toxicity** | 0.849 | 0.039 | 0.176 | 0.081 | 0.19 | 0.201 | 0.017 | 0.195 | 0.072 | 0.048 |
| **FDAMDD** | 0.012 | 0.026 | 0.314 | 0.012 | 0.947 | 0.938 | 0.019 | 0.942 | 0.151 | 0.045 |
| **Skin Sensitization** | 0.299 | 0.928 | 0.642 | 0.868 | 0.919 | 0.726 | 0.157 | 0.903 | 0.322 | 0.324 |
| **Carcinogencity** | 0.191 | 0.536 | 0.056 | 0.276 | 0.033 | 0.029 | 0.042 | 0.035 | 0.496 | 0.124 |
| **Eye Corrosion** | 0.048 | 0.672 | 0.532 | 0.913 | 0.003 | 0.003 | 0.062 | 0.003 | 0.222 | 0.003 |
| **Eye Irritation** | 0.99 | 0.977 | 0.983 | 0.993 | 0.004 | 0.004 | 0.948 | 0.004 | 0.898 | 0.043 |
| **Respiratory Toxicity** | 0.174 | 0.188 | 0.819 | 0.225 | 0.402 | 0.546 | 0.04 | 0.442 | 0.069 | 0.041 |
| **Environmental Toxicity** | | | | | | | | | | |
| **Bioconcentration Factors** | 0.348 | 0.664 | 0.600 | 0.441 | 1.149 | 1.258 | 0.427 | 1.342 | 1.407 | 0.530 |
| **IGC50** | 2.690 | 3.846 | 3.453 | 3.302 | 5.391 | 5.341 | 2.072 | 5.384 | 3.310 | 3.449 |
| **LC50FM** | 2.966 | 4.324 | 4.244 | 3.839 | 6.748 | 6.973 | 2.761 | 6.751 | 3.648 | 3.694 |
| **LC50DM** | 3.278 | 5.411 | 4.410 | 4.010 | 7.258 | 7.394 | 2.816 | 7.214 | 5.198 | 4.895 |
| **Tox21 Pathway** | | | | | | | | | | |
| **NR-AR** | 0.029 | 0.103 | 0.013 | 0.611 | 0.59 | 0.664 | 0.082 | 0.665 | 0.14 | 0.019 |
| **NR-AR-LBD** | 0.014 | 0.872 | 0.015 | 0.721 | 0.012 | 0.013 | 0.042 | 0.012 | 0.028 | 0.052 |
| **NR-AhR** | 0.107 | 0.253 | 0.835 | 0.126 | 0.467 | 0.35 | 0.033 | 0.456 | 0.328 | 0.448 |
| **NR-Aromatase** | 0.004 | 0.141 | 0.008 | 0.022 | 0.481 | 0.497 | 0.043 | 0.474 | 0.182 | 0.045 |
| **NR-ER** | 0.112 | 0.902 | 0.221 | 0.62 | 0.192 | 0.158 | 0.048 | 0.217 | 0.1 | 0.454 |
| **NR-ER-LBD** | 0.007 | 0.822 | 0.267 | 0.391 | 0.128 | 0.089 | 0.007 | 0.089 | 0.01 | 0.037 |
| **NR-PPAR-gamma** | 0.005 | 0.717 | 0.146 | 0.092 | 0.007 | 0.007 | 0.007 | 0.008 | 0.004 | 0.085 |
| **SR-ARE** | 0.027 | 0.903 | 0.113 | 0.788 | 0.546 | 0.624 | 0.038 | 0.559 | 0.079 | .0.129 |
| **SR-ATAD5** | 0.019 | 0.953 | 0.071 | 0.798 | 0.074 | 0.076 | 0.115 | 0.113 | 0.272 | 0.695 |
| **SR-HSE** | 0.119 | 0.491 | -0.426 | 0.065 | 0.015 | 0.004 | 0.098 | 0.022 | 0.019 | 0.04 |
| **SR-MMP** | 0.019 | 0.441 | 0.263 | 0.153 | 0.739 | 0.663 | 0.015 | 0.745 | 0.034 | 0.294 |
| **SR-p53** | 0.013 | 0.91 | 0.076 | 0.495 | 0.813 | 0.773 | 0.013 | 0.834 | 0.61 | 0.335 |
| **Toxicophore Rules Alerts** | | | | | | | | | | |
| **Acute Toxicity Rule** | 0 | 0 | 0 | 0 | 0 | 0 | 0 | 0 | 0 | 0 |
| **Genotoxic Carcinogenicity Rule** | 0 | 1 | 0 | 0 | 0 | 0 | 0 | 0 | 1 | 0 |
| **NonGenotoxic Carcinogenicity Rule** | 0 | 1 | 0 | 1 | 0 | 0 | 0 | 0 | 0 | 0 |
| **Skin Sensitization Rule** | 0 | 3 | 5 | 2 | 4 | 1 | 2 | 1 | 1 | 1 |
| **Aquatic Toxicity Rule** | 0 | 3 | 0 | 2 | 0 | 0 | 0 | 0 | 0 | 0 |
| **NonBiodegradable Rule** | 0 | 0 | 1 | 0 | 0 | 0 | 0 | 0 | 0 | 0 |
| **SureChEMBL Rule** | 0 | 0 | 0 | 0 | 0 | 0 | 0 | 0 | 0 | 0 |
| **FAF-Drugs4 Rule** | 0 | 2 | 1 | 2 | 1 | 1 | 0 | 1 | 1 | 1 |
