# Supplementary material for: Computational analysis of Ayurvedic metabolites for potential treatment of drug-resistant Candida auris
Source: Front Cell Infect Microbiol. 2025 Mar 13;15:1537872. doi: 10.3389/fcimb.2025.1537872 (PMC11979702; doi:10.3389/fcimb.2025.1537872)
Supplement: Supplementary file 9 [file Table9.docx]

**Table S9.** Target prediction analysis of top metabolites by Swiss target prediction tool.

| **Sr. No** | **Target** | **Common Name** | **Target class** | **Probability** |
| --- | --- | --- | --- | --- |
| **1** | **4-hydroxybenzoate** | | | |
|  | Carbonic anhydrase II | CA2 | Lyase | 80.0% |
|  | Carbonic anhydrase VII | CA7 | Lyase | 80.0% |
|  | Carbonic anhydrase I | CA1 | Lyase | 80.0% |
|  | Carbonic anhydrase III | CA3 | Lyase | 80.0% |
|  | Carbonic anhydrase VI | CA6 | Lyase | 80.0% |
|  | Carbonic anhydrase XII | CA12 | Lyase | 80.0% |
|  | Carbonic anhydrase XIV | CA14 | Lyase | 80.0% |
|  | Carbonic anhydrase IX | CA9 | Lyase | 80.0% |
|  | Carbonic anhydrase IV | CA4 | Lyase | 80.0% |
|  | Carbonic anhydrase VB | CA5B | Lyase | 80.0% |
|  | Carbonic anhydrase VA | CA5A | Lyase | 80.0% |
|  | Carbonic anhydrase XIII | CA13 | Lyase | 80.0% |
| **2** | **Methylcoumarate** | | | |
|  | Carbonic anhydrase II | CA2 | Lyase | 66.7% |
|  | Carbonic anhydrase VII | CA7 | Lyase | 66.7% |
|  | Carbonic anhydrase I | CA1 | Lyase | 66.7% |
|  | Carbonic anhydrase XII | CA12 | Lyase | 66.7% |
|  | Carbonic anhydrase XIV | CA14 | Lyase | 66.7% |
|  | Carbonic anhydrase IX | CA9 | Lyase | 66.7% |
|  | Carbonic anhydrase IV | CA4 | Lyase | 66.7% |
|  | Carbonic anhydrase VB | CA5B | Lyase | 66.7% |
|  | Carbonic anhydrase VA | CA5A | Lyase | 66.7% |
|  | Carbonic anhydrase VI | CA6 | Lyase | 66.7% |
| **3** | **2,6-Dihydroxy-4-methoxyacetophenone** | | | |
|  | Serine/threonine-protein kinase/endoribonuclease IRE1 | ERN1 | Enzyme | 13.3% |
|  | Cannabinoid receptor 1 | CNR1 | Family A G protein-coupled receptor | 13.3% |
|  | Cannabinoid receptor 2 | CNR2 | Family A G protein-coupled receptor | 13.3% |
|  | Carbonic anhydrase II | CA2 | Lyase | 40.0% |
|  | Carbonic anhydrase I | CA1 | Lyase | 40.0% |
|  | Carbonic anhydrase VII | CA7 | Lyase | 40.0% |
|  | Carbonic anhydrase XII | CA12 | Lyase | 40.0% |
|  | Carbonic anhydrase XIV | CA14 | Lyase | 40.0% |
|  | Carbonic anhydrase IX | CA9 | Lyase | 40.0% |
| **4** | **trans-p-coumaric acid** | | | |
|  | Aldose reductase | AKR1B1 | Enzyme | 13.3% |
|  | Carbonic anhydrase II | CA2 | Lyase | 73.3% |
|  | Carbonic anhydrase VII | CA7 | Lyase | 73.3% |
|  | Carbonic anhydrase I | CA1 | Lyase | 73.3% |
|  | Carbonic anhydrase III | CA3 | Lyase | 73.3% |
|  | Carbonic anhydrase VI | CA6 | Lyase | 73.3% |
|  | Carbonic anhydrase XII | CA12 | Lyase | 73.3% |
|  | Carbonic anhydrase XIV | CA14 | Lyase | 73.3% |
|  | Carbonic anhydrase IX | CA9 | Lyase | 73.3% |
|  | Carbonic anhydrase IV | CA4 | Lyase | 73.3% |
|  | Carbonic anhydrase VB | CA5B | Lyase | 73.3% |
|  | Carbonic anhydrase VA | CA5A | Lyase | 73.3% |
| **5** | **Isoliensinine** | | | |
|  | Dopamine transporter | SLC6A3 | Electrochemical transporter | 26.7% |
|  | Dopamine D2 receptor | DRD2 | Family A G protein-coupled receptor | 60.0% |
|  | Multidrug and toxin extrusion protein 1 | SLC47A1 | Electrochemical transporter | 26.7% |
|  | Dopamine D1 receptor | DRD1 | Family A G protein-coupled receptor | 60.0% |
|  | Serotonin 1a (5-HT1a) receptor | HTR1A | Family A G protein-coupled receptor | 60.0% |
| **6** | **Neferine** | | | |
|  | Dopamine D2 receptor | DRD2 | Family A G protein-coupled receptor | 46.7% |
|  | Dopamine transporter | SLC6A3 | Electrochemical transporter | 13.3% |
|  | Multidrug and toxin extrusion protein 1 | SLC47A1 | Electrochemical transporter | 13.3% |
|  | Neuronal acetylcholine receptor; alpha3/beta4 | CHRNA3 CHRNB4 | Ligand-gated ion channel | 26.7% |
|  | Serotonin 1a (5-HT1a) receptor | HTR1A | Family A G protein-coupled receptor | 46.7% |
| **7** | **Eudesmic acid / 3,4,5-Trimethoxybenzoic acid** | | | |
|  | Carbonic anhydrase II | CA2 | Lyase | 73.3% |
|  | Carbonic anhydrase VII | CA7 | Lyase | 73.3% |
|  | Carbonic anhydrase I | CA1 | Lyase | 73.3% |
|  | Carbonic anhydrase XII | CA12 | Lyase | 73.3% |
|  | Carbonic anhydrase XIV | CA14 | Lyase | 73.3% |
|  | Carbonic anhydrase IX | CA9 | Lyase | 73.3% |
|  | Carbonic anhydrase IV | CA4 | Lyase | 73.3% |
|  | Carbonic anhydrase VA | CA5A | Lyase | 73.3% |
|  | Carbonic anhydrase III | CA3 | Lyase | 73.3% |
|  | Carbonic anhydrase VI | CA6 | Lyase | 73.3% |
|  | Carbonic anhydrase XIII | CA13 | Lyase | 73.3% |
| **8** | **Liensinine** | | | |
|  | Dopamine D2 receptor | DRD2 | Family A G protein-coupled receptor | 53.3% |
|  | Dopamine transporter | SLC6A3 | Electrochemical transporter | 20.0% |
|  | Dopamine D1 receptor | DRD1 | Family A G protein-coupled receptor | 53.3% |
|  | Alpha-1d adrenergic receptor | ADRA1D | Family A G protein-coupled receptor | 53.3% |
|  | Multidrug and toxin extrusion protein 1 | SLC47A1 | Electrochemical transporter | 20.0% |
| **9** | **Scoparone** | | | |
|  | Carbonic anhydrase XII | CA12 | Lyase | 73.3% |
|  | Carbonic anhydrase IX | CA9 | Lyase | 73.3% |
|  | Carbonic anhydrase XIII | CA13 | Lyase | 73.3% |
|  | Carbonic anhydrase VII | CA7 | Lyase | 73.3% |
|  | Carbonic anhydrase XIV | CA14 | Lyase | 73.3% |
|  | Carbonic anhydrase I | CA1 | Lyase | 73.3% |
|  | Carbonic anhydrase VI | CA6 | Lyase | 73.3% |
|  | Carbonic anhydrase VB | CA5B | Lyase | 73.3% |
|  | Carbonic anhydrase VA | CA5A | Lyase | 73.3% |
|  | Carbonic anhydrase IV | CA4 | Lyase | 73.3% |
|  | Carbonic anhydrase II | CA2 | Lyase | 73.3% |
| **10** | **(R)-N-(1’-methoxycarbonyl-2’-phenylethyl)-4-hydroxybenzamide** | | | |
|  | C-C chemokine receptor type 3 | CCR3 | Family A G protein-coupled receptor | 6.7% |
|  | Cystinyl aminopeptidase | LNPEP | Protease | 26.7% |
|  | Matrix metalloproteinase 2 | MMP2 | Protease | 26.7% |
|  | Intercellular adhesion molecule (ICAM-1), Integrin alpha-L/beta-2 | ITGAL ICAM1 ITGB2 | Membrane receptor | 20.0% |
|  | Protein-tyrosine phosphatase 1B | PTPN1 | Phosphatase | 6.7% |
