# Supplementary figures and images for: Computational analysis of Ayurvedic metabolites for potential treatment of drug-resistant Candida auris
Source: Front Cell Infect Microbiol. 2025 Mar 13;15:1537872. doi: 10.3389/fcimb.2025.1537872 (PMC11979702; doi:10.3389/fcimb.2025.1537872)

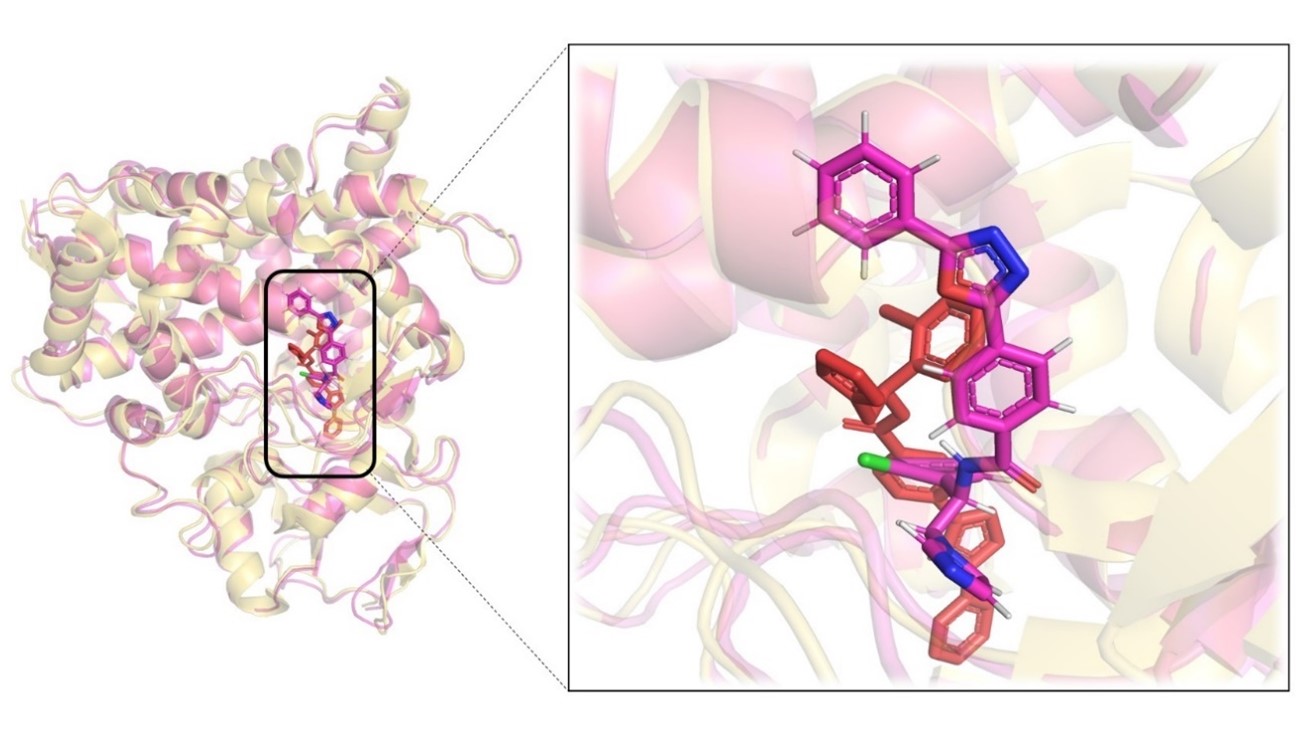

Supplement: Supplementary Figure 1 — Superimposing the docked complex (in red) onto the co-crystallized complex (in gold) within the active site using PyMOL (RMSD = 0.980 A0). [file Image1.jpg]

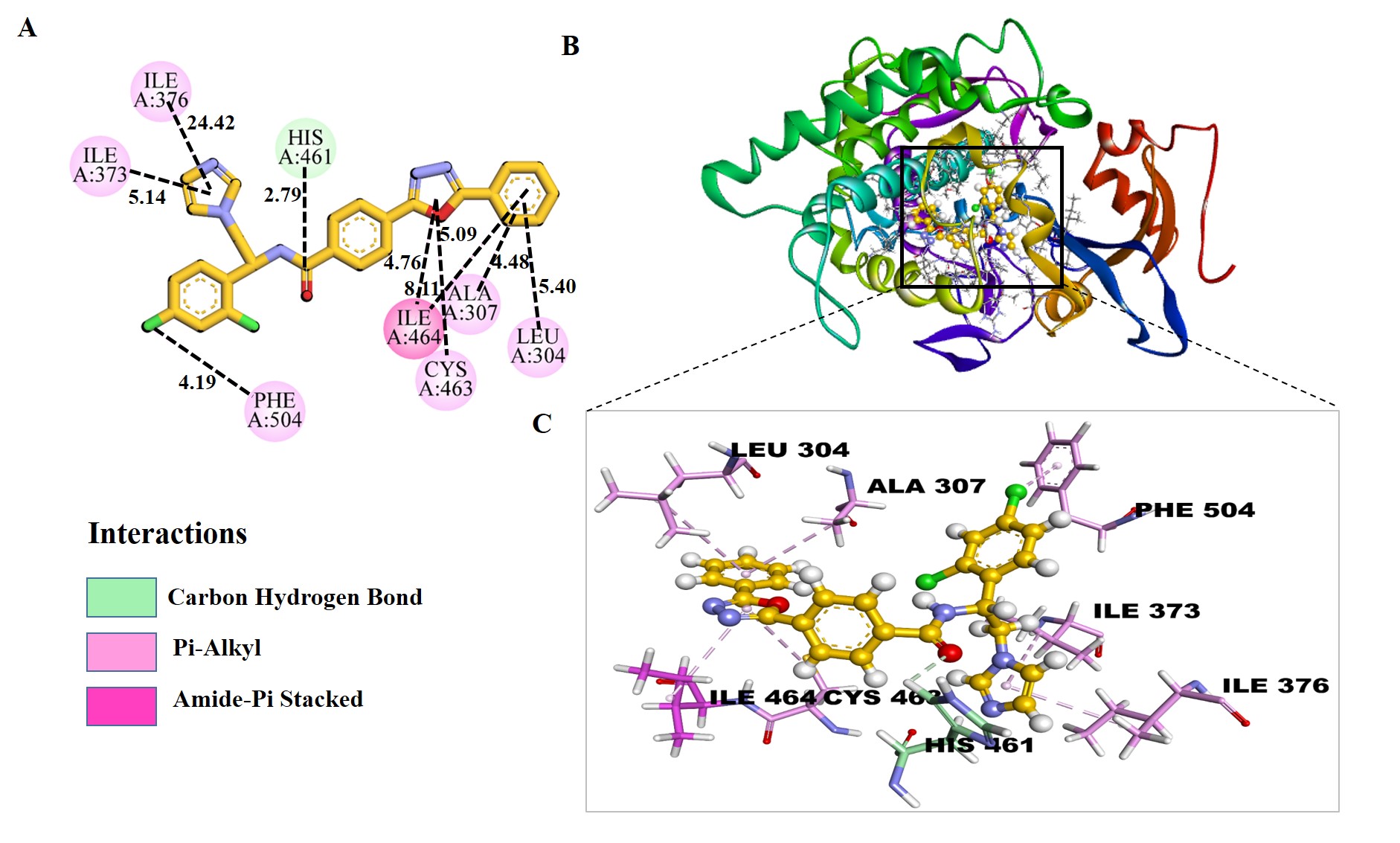

Supplement: Supplementary Figure 2 — Protein-Ligand Interaction analysis of metabolite VNI; 2D schematic interactions with bond distance (A); 3D interactions with amino acids residues (B) and 3D conformation (C) in the binding pocket of the target protein. [file Image2.jpg]

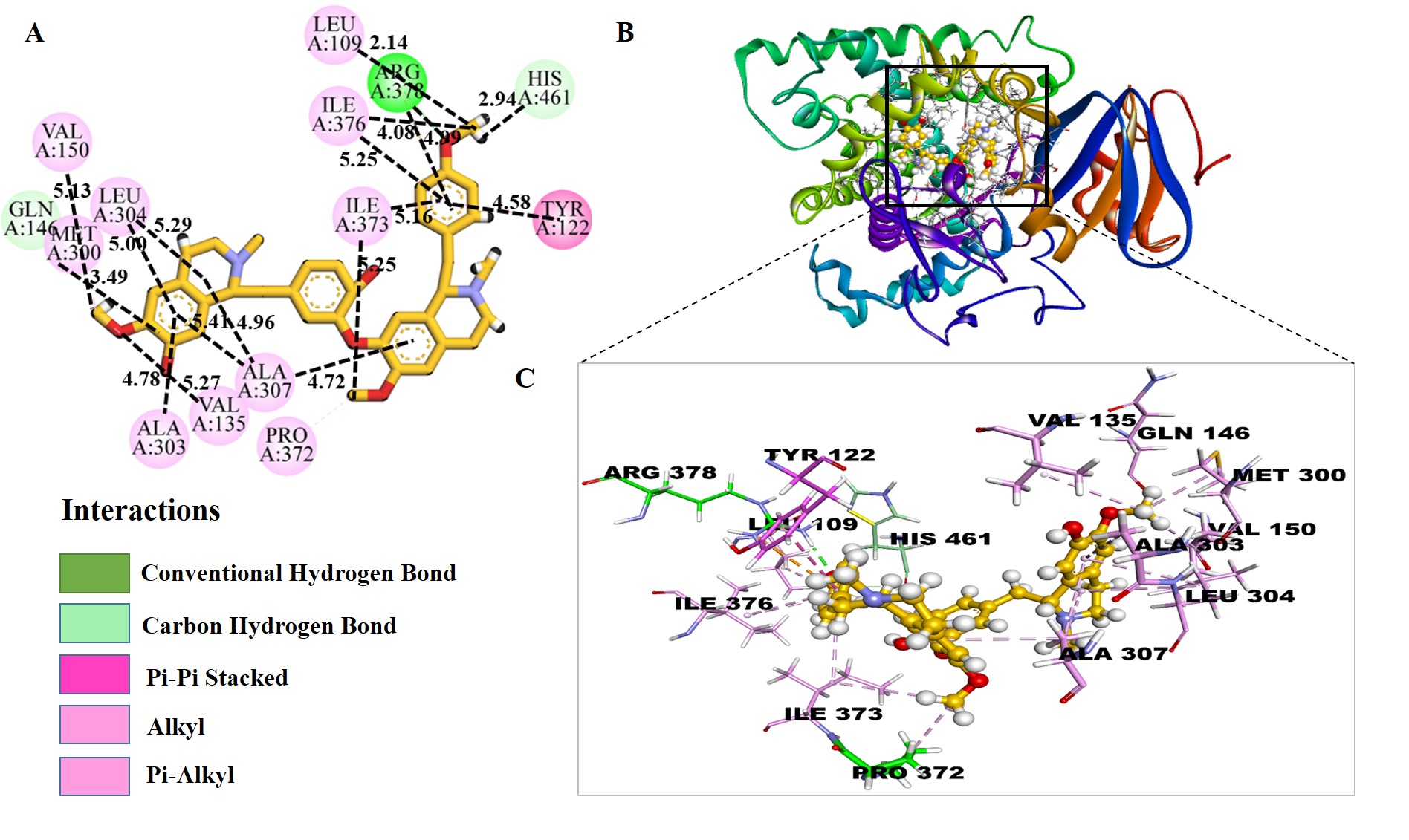

Supplement: Supplementary Figure 3 — Protein-Ligand Interaction analysis of metabolite Isoliensinine; 2D schematic interactions with bond distance (A); 3D interactions with amino acids residues (B) and 3D conformation (C) in the binding pocket of the target protein. [file Image3.jpg]

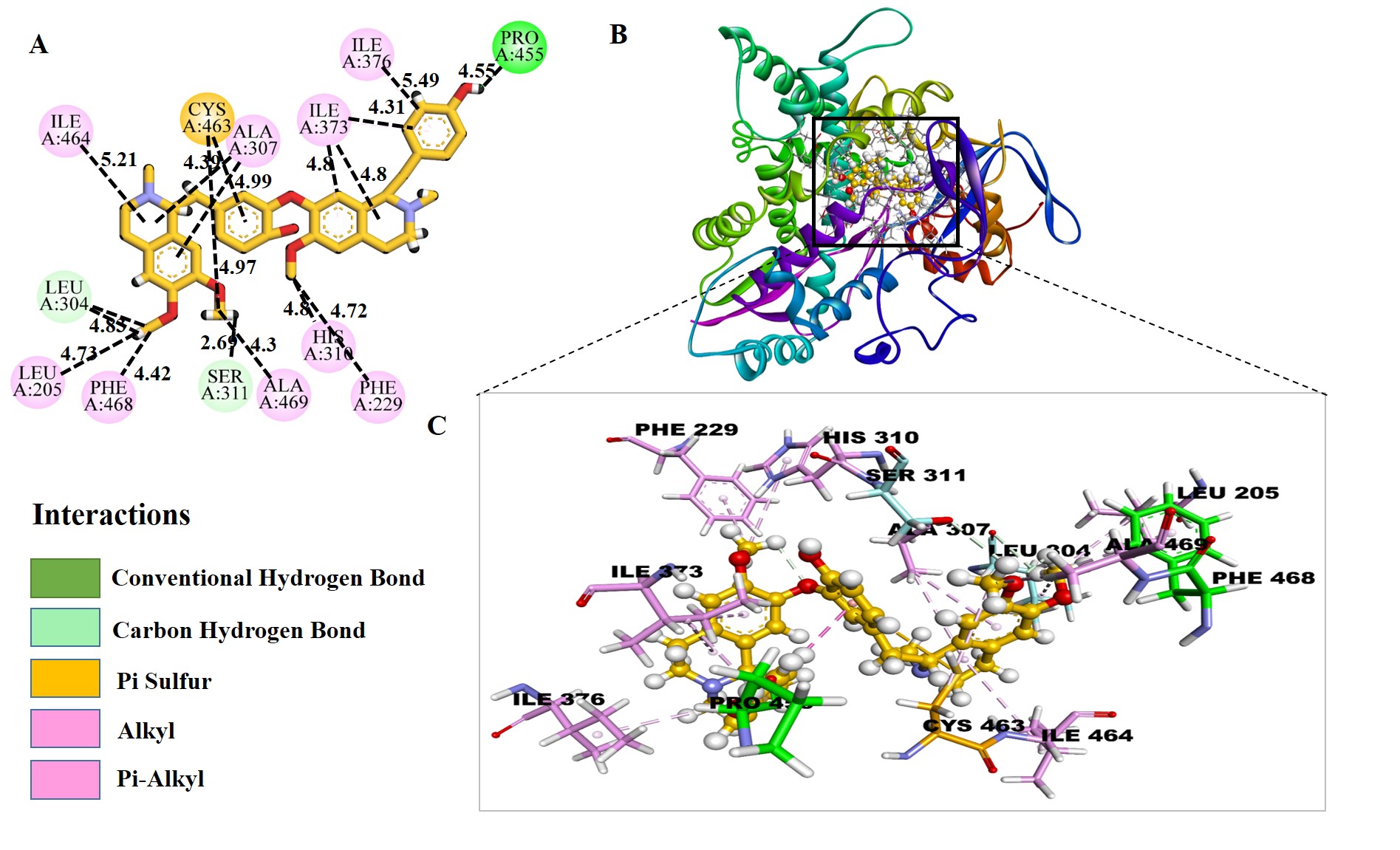

Supplement: Supplementary Figure 4 — Protein-Ligand Interaction analysis of metabolite Liensinine; 2D schematic interactions with bond distance (A); 3D interactions with amino acids residues (B); and 3D conformation (C) in the binding pocket of the target protein. [file Image4.jpg]

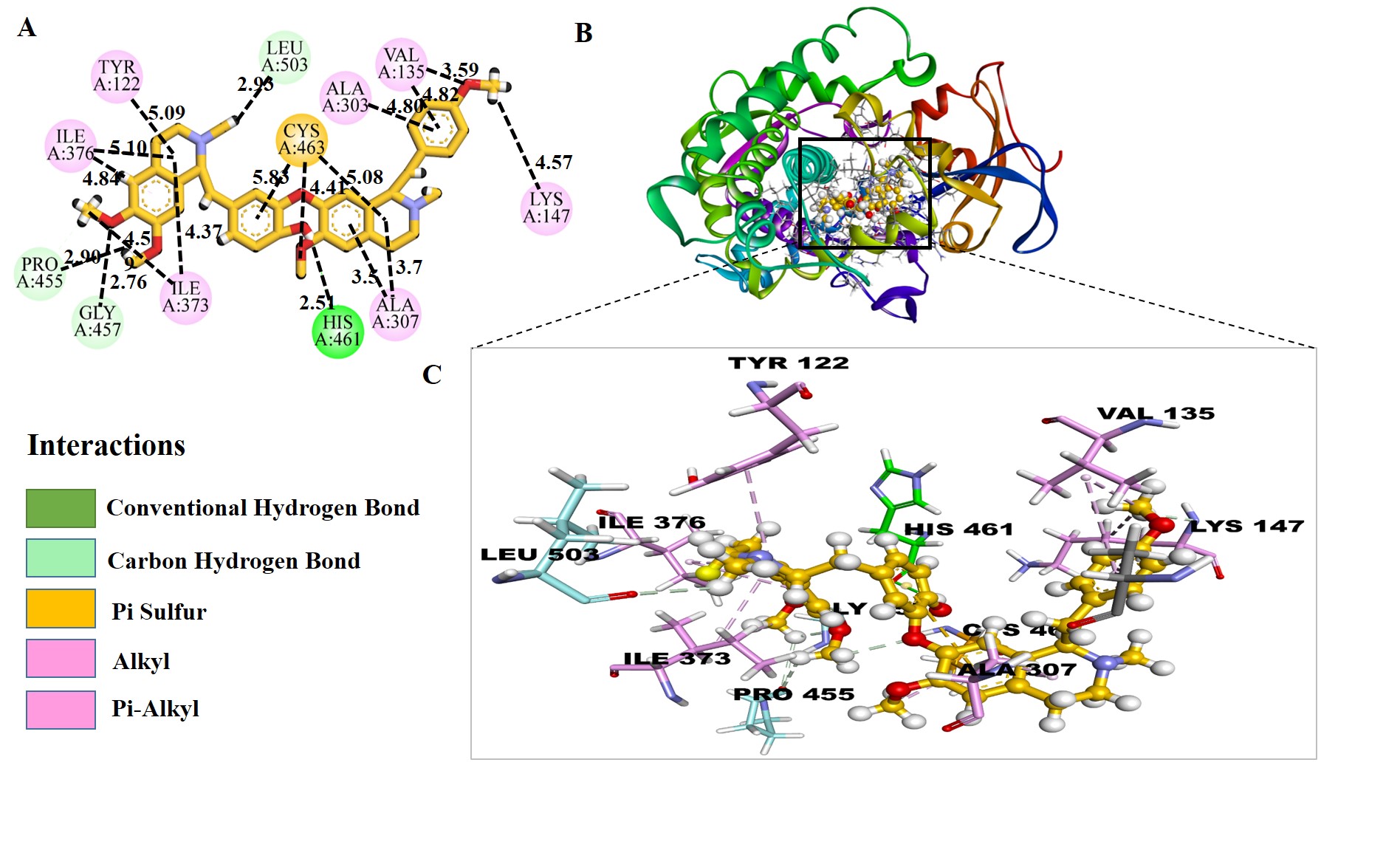

Supplement: Supplementary Figure 5 — Protein-Ligand Interaction analysis of metabolite Neferine; 2D schematic interactions with bond distance (A); 3D interactions with amino acids residues (B); and 3D conformation (C) in the binding pocket of the target protein. [file Image5.jpg]

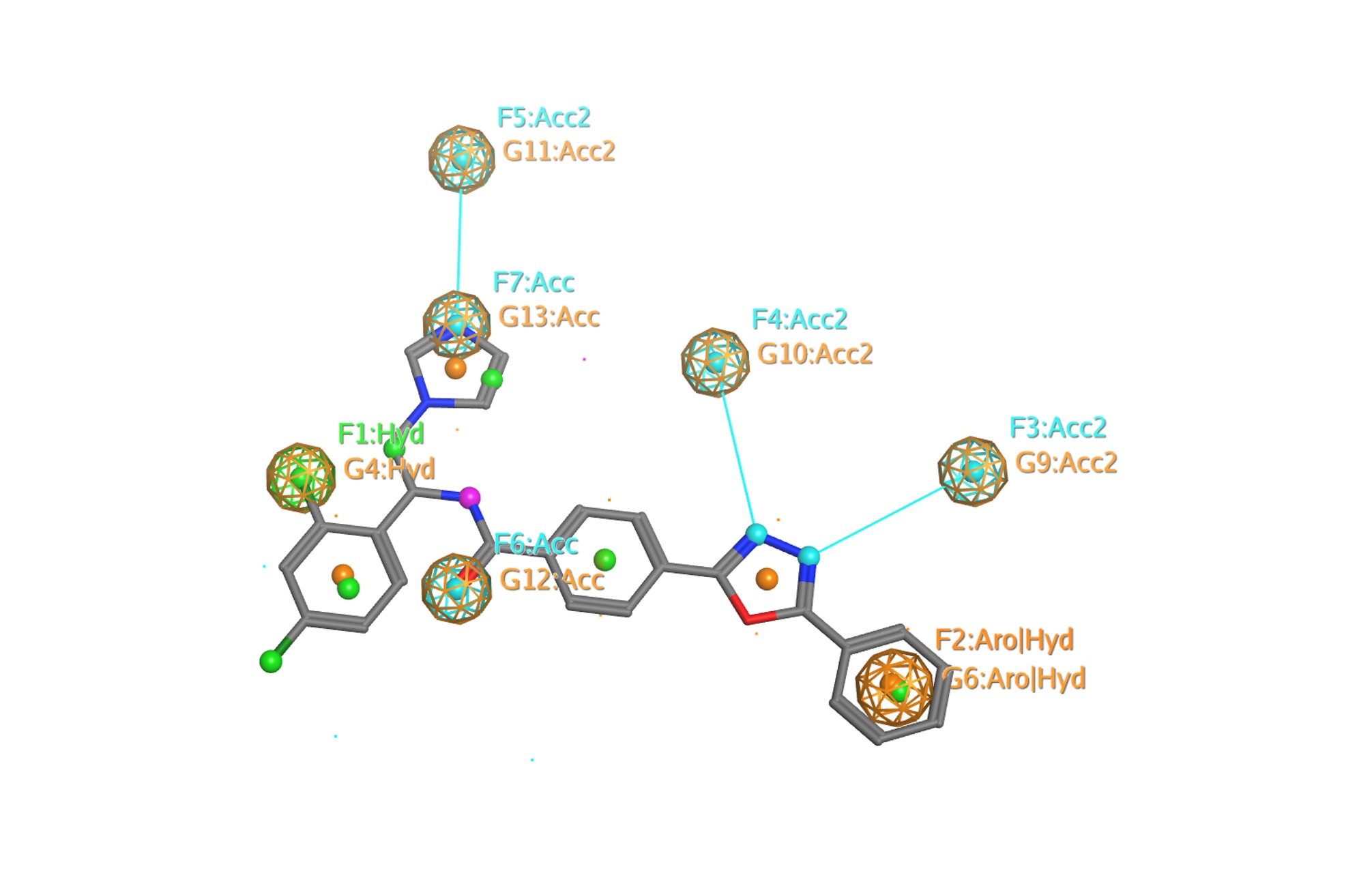

Supplement: Supplementary Figure 6 — Essential pharmacophoric features of the co-crystallized ligand and the standard compound, VNI, for the inhibition of target protein. [file Image6.jpg]

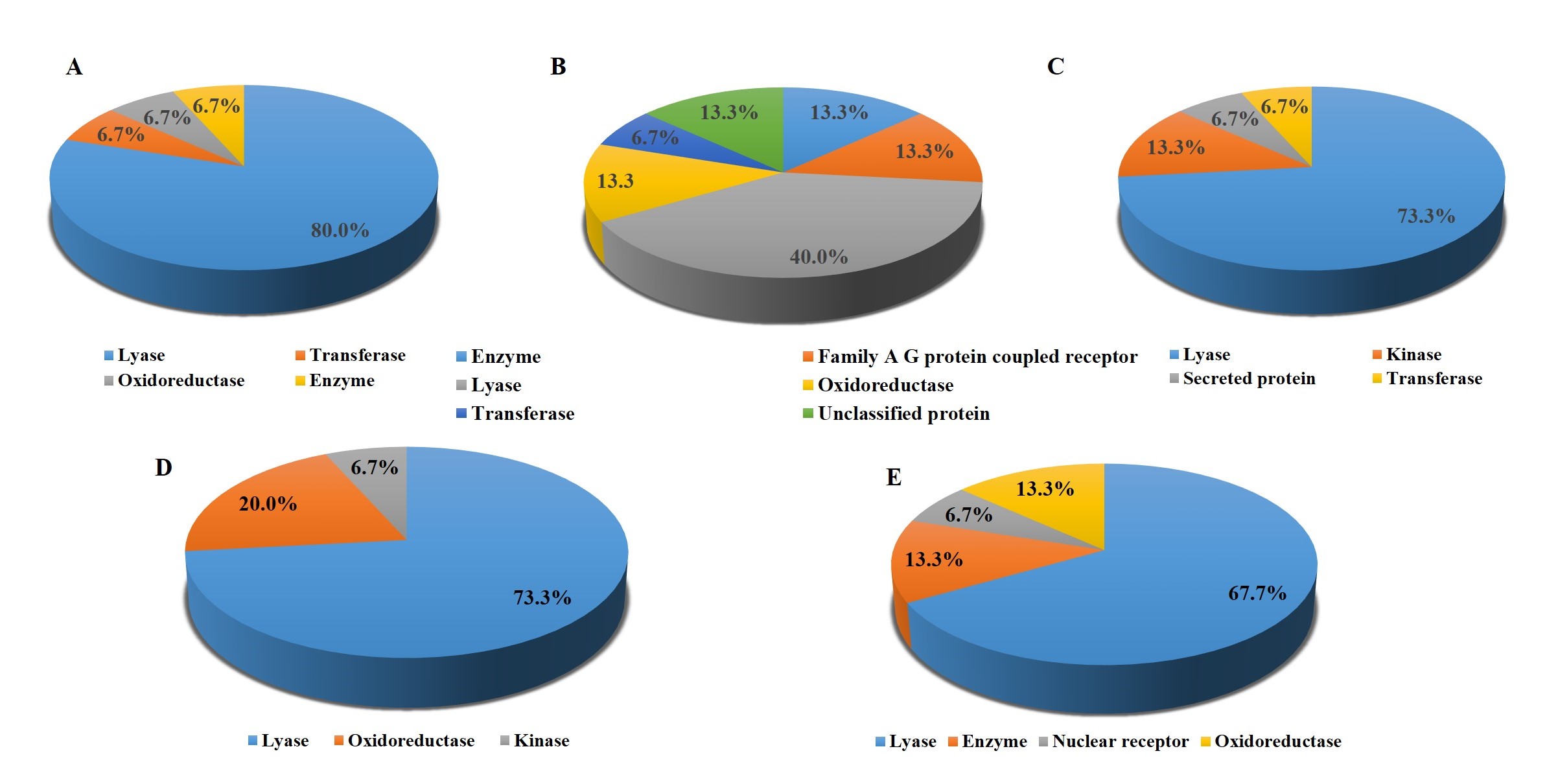

Supplement: Supplementary Figure 7 — Target prediction of molecules (A) 4-hydroxybenzoate, (B) trans-p-coumaric, (C) eudesmic acid/3,4,5-Trimethoxybenzoic acid, (D) scoparone, and (E) methylcoumarate with different receptor proteins shown in a pie chart diagram. [file Image7.jpg]
